# Shielding Si Armed with CoSi_2_ Nanoplates and Dual‐Carbon Shells: 3D Porous Microspheres as High‐Performance Anodes for Li‐Ion Batteries

**DOI:** 10.1002/advs.202508213

**Published:** 2025-07-29

**Authors:** Jae Seob Lee, Hyun Seon Ahn, Jung Yeon Kim, Rakesh Saroha, Beom Su Jo, Ji Hun Baek, Yun Chan Kang, Gi Dae Park, Dae Soo Jung, Dong‐Won Kang, Chungyeon Cho, Jung Sang Cho

**Affiliations:** ^1^ Department of Engineering Chemistry Chungbuk National University Cheongju Chungbuk 28644 Republic of Korea; ^2^ Department of Materials Science and Engineering Korea University Seoul 02841 Republic of Korea; ^3^ Department of Materials Science and Engineering Ajou University Suwon‐si Gyeonggi‐do 16499 Republic of Korea; ^4^ Department of Advanced Materials Engineering Chungbuk National University Cheongju Chungbuk 28644 Republic of Korea; ^5^ Energy Storage Materials Center Korea Institute of Ceramic Engineering and Technology Jinju Gyeongnam 52851 Republic of Korea; ^6^ School of Energy Systems Engineering Chung‐Ang University Dongjak‐Gu Seoul 06974 Republic of Korea; ^7^ Department of Biomedical Materials Science Jeonbuk Advanced Bio‐convergence Academy Wonkwang University Iksan Jeonbuk 54538 Republic of Korea; ^8^ Biomedical Research Institute Chungbuk National University Hospital Cheongju Chungbuk 28644 Republic of Korea; ^9^ Advanced Energy Research Institute Chungbuk National University Cheongju Chungbuk 28644 Republic of Korea

**Keywords:** full‐cell, lithium‐ion batteries, metal silicide, nitrogen‐doped graphitic carbon, polydopamine‐derived carbon shell, silicon anodes

## Abstract

In this study, a novel multi‐core–dual‐shell strategy is employed to synthesize 3D porous microspheres. These microspheres consist of Si armed with CoSi_2_ nanoplates multi‐cores encapsulated within dual protective shells of metallic‐Co nanocrystals embedded nitrogen‐doped graphitic carbon (NGC) and polydopamine‐derived carbon (PDA‐C), denoted as Si@CoSi_2_‐Co/NGC@PDA‐C, through a multistep synthesis process involving facile spray pyrolysis and post‐heat‐treatment. The Si as an active material is surrounded by an inactive buffer material of CoSi_2_ with intrinsic low bulk resistivity and high chemical stability, enhancing the electrical interconnectivity and mechanical integrity of the nanostructure. The metallic‐Co embedded porous NGC shell facilitates rapid electron transfer by providing primary transport pathways due to its high conductivity, while improving structural robustness by partially restraining the volume expansion of Si. Finally, an additional PDA‐derived C protective shell provides secondary transport pathways while mitigating volume expansion, preventing complete detachment from the current collector or pulverization. Correspondingly, the Si@CoSi_2_‐Co/NGC@PDA‐C anode exhibits considerable rate capability (up to 10 A g^−1^) and remarkable cycling stability (88% capacity retention after 600 cycles; average capacity loss of 0.02% per cycle at 1.0 A g^−1^). Moreover, full‐cells paired with a Li(Ni_0.8_Co_0.1_Mn_0.1_)O_2_ cathode are evaluated to confirm the practical viability of the nanostructures.

## Graphical abstract

The lithium‐ion battery anode is designed utilizing 3D porous microspheres comprising Si armed with CoSi_2_ multi‐cores encapsulated within dual protective shells of metallic‐Co embedded nitrogen‐doped graphitic carbon (NGC) and polydopamine‐derived carbon (PDA‐C). By employing a combination of CoSi_2_, metallic‐Co embedded NGC, and PDA‐C, challenges including the low electronic conductivity and substantial volume expansion of Si are overcome, resulting in outstanding performances.

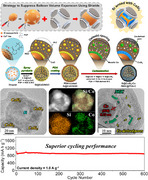

## 1 Introduction

Conventional graphite has long been the preferred choice for anodes in commercial lithium‐ion batteries (LIBs) due to its overwhelming electrochemical performance, including stable cycling, minimal initial capacity loss, and low volumetric expansion (<17%) during charge–discharge processes.^[^ ^1^, ^2^ ^]^ However, the relatively low theoretical discharge capacity of conventional graphite anodes (372 mAh g^−1^) and the occurrence of severe lithium (Li) deposition during redox reactions have prompted researchers to explore alternative materials.^[^ ^3^, ^4^, ^5^ ^]^ Li‐alloying electrochemical reactions have demonstrated tremendous possibilities due to their comparable discharge potential to Li/Li^+^ and high discharge capacity.^[^ ^6^ ^]^ Among the various alloy candidates, silicon (Si) has garnered substantial interest as a promising anode material for next‐generation commercial LIBs. Si offers several advantages, including a high volumetric (2190 mAh cm^−3^) and gravimetric (3579 mAh g^−1^) capacity when fully lithiated (Li_15_Si_4_), a moderate discharge potential (0.37 V vs Li/Li^+^), natural abundance, and nontoxicity.^[^ ^7^, ^8^, ^9^, ^10^, ^11^ ^]^ However, its commercial viability is mainly hindered by its low electronic conductivity (10^−3^ S cm^−1^), inferior Li‐ion diffusivity (≈10^−14^ cm^2^ s^−1^), and substantial volume variation (≈360%) during charge and discharge processes.^[^ ^12^, ^13^, ^14^, ^15^, ^16^ ^]^ The repeated volume changes result in severe pulverization of the electrode material, leading to low Coulombic efficiency (CE).^[^ ^17^ ^]^ Additionally, the mechanical stress and strain induced by the volume fluctuations cause extensive damage to the solid electrolyte interphase (SEI), resulting in high cell impedance and poor electrochemical activity due to deposition of insulating products.

To mitigate the pulverization of the Si anodes caused by significant volume changes, various strategies have been explored. These includes size‐controlled Si anodes with the nanometer scale,^[^ ^18^, ^19^ ^]^ porous Si‐based nanostructures,^[^ ^20^, ^21^ ^]^ thin films with high mechanical integrity,^[^ ^22^, ^23^ ^]^ composite with carbon,^[^ ^24^, ^25^ ^]^ and diverse morphological designs such as core–shell structures,^[^ ^26^ ^]^ nano‐microwires,^[^ ^27^ ^]^ nanoplates,^[^ ^28^ ^]^ and hollow nanospheres,^[^ ^29^ ^]^ etc. Among these, the core–shell strategy has gained particular attention due to its dual advantages: it effectively enhances electrochemical performance while maintaining cost‐efficiency, making it promising for commercialization. Core–shell structures typically involve a Si‐core surrounded by multifunctional outer shells or vice versa. For instance, Hu et al. prepared a double carbon‐coated silicon/carbon/metal–organic framework multi‐core mesoporous composite anode, which exhibits an initial discharge capacity of ≈2600 mAh g^−1^ at 0.1 A g^−1^ with reasonable cycling performance (300 cycles at 1.0 A g^−1^).^[^ ^30^ ^]^ Conversely, Cui et al. synthesized a core–shell TiC/C/Si structure, where the outer Si shell serves as the active component, while the highly conductive and robust TiC/C core nanowire arrays robust the mechanical properties.^[^ ^31^ ^]^ This core–shell composite nanostructure achieved a high discharge capacity of ≈3000 mAh g^−1^ at 0.84 A g^−1^ with moderate cycling stability (100 cycles at 0.84 A g^−1^). However, conventional core–shell structures also face several key limitations. First, the carbon shell can suffer from mechanical rupture due to the large volume expansion of Si during cycling, leading to structural failure and electrical isolation.^[^ ^19^ ^]^ Second, many carbon coatings, especially amorphous or polymer‐derived types, possess low intrinsic conductivity, which restricts high‐rate performance.^[^ ^32^ ^]^ Third, the interfacial adhesion between the Si core and the shell is often insufficient, which can lead to delamination and unstable SEI formation over prolonged cycling.^[^ ^33^ ^]^ These issues significantly hinder long‐term cycling stability and electrochemical kinetics. To overcome these intrinsic limitations of conventional core–shell designs, we introduce Si nanocrystals armed with silicon‐based cobalt disilicide (CoSi_2_) inactive nanoplates (Si@CoSi_2_ multi‐cores) to construct a multi‐core dual‐shell structure. CoSi_2_ offers unique advantages as an inactive protective material: 1) its low bulk resistivity (10–25 µΩ cm) enhances electrical interconnection; 2) its high thermal and chemical stability minimizes reactivity with Li at room temperature; and 3) its robust mechanical properties ensure structural stability during cycling.^[^ ^5^, ^34^, ^35^ ^]^ These attributes make CoSi_2_ an ideal candidate for reinforcing the mechanical and electrochemical properties of Si anodes. To further enhance the cycling stability and electrochemical performance of the anodes, the Si@CoSi_2_ multi‐cores were encapsulated within dual protective shells: an inner nitrogen‐doped graphitic carbon (NGC) layer, formed via an in situ catalytic process utilizing Co‐species, and an outer polydopamine‐derived nitrogen‐doped carbon (PDA‐C) layer, added through an ex situ coating process. This dual‐shell configuration addresses the drawbacks of traditional core–shell systems by offering: 1) the NGC shell serves as the primary transport pathway, facilitating the direct transfer of electron from the active Si nanocrystals, whereas the PDA‐C coating, acting as the secondary pathway, further promotes electron transfer to the current collector; 2) the nitrogen content in the coating material enhances the overall conductivity of the anode, ensuring rapid redox reaction; 3) the dual shells protect the Si particles from pulverization, maintaining structural integrity and promoting the formation of a stable solid electrolyte interphase layer, which is critical for consistent cycling stability.

To validate this innovative design, we synthesized 3D porous microspheres comprising Si@CoSi_2_ multi‐cores encapsulated within dual protective shells of metallic‐Co nanocrystals embedded highly conductive NGC and PDA‐C (Si@CoSi_2_‐Co/NGC@PDA‐C) using a cost‐effective, scalable aerosol‐assisted spray pyrolysis technique followed by a solution‐based PDA coating and carbonization. This process yielded a highly robust anode material with exceptional performance metrics: a high discharge capacity of 2181 mAh g^−1^ at 0.1 A g^−1^, excellent high‐rate capability (up to 10 A g^−1^), and impressive cycling stability (88% capacity retention after 600 cycles at 1.0 A g^−1^ and 70% after 1000 cycles at 3.0 A g^−1^). Additionally, full‐cell tests paired with a Li(Ni_0.8_Co_0.1_Mn_0.1_)O_2_ cathode demonstrated the practical feasibility of the proposed design for commercial applications. This study provides a transformative approach to addressing the intrinsic challenges of Si anodes. The rationally designed Si@CoSi_2_‐Co/NGC@PDA‐C hybrid nanostructure combines innovative material engineering with scalable fabrication methods, paving the way for next‐generation high‐performance Li‐ion battery anodes.

## 2 Results and Discussion

To comprehensively understand the formation process, the thorough morphology and crystal structure analysis of the synthesized nanostructure at each stage of the synthesis process were conducted. First, the as‐received Si nanopowder was analyzed for microstructural and phase analysis, as shown in Figure S1 (Supporting Information). The powder exhibits spherical‐type morphology (Figure S1a, Supporting Information) with a particle size distribution in the range of 50–100 nm. Additionally, the X‐ray diffraction (XRD) pattern (Figure S1b, Supporting Information) revealed the phase purity of the powder with high and intense peaks associated with Si only. Using Si powder, the 3D microspheres comprising Si@CoO multi‐cores enveloped within the NGC shell (denoted as Si@CoO/NGC microspheres) were prepared via aerosol‐assisted spray pyrolysis (Scheme S1a, Supporting Information). Briefly, an aqueous precursor solution containing Co(NO_3_)_2_ (0.15 m), Si nanopowder, and polyvinylpyrrolidone (PVP) as the carbon source was ultrasonically atomized. The resulting aerosol droplets were carried by nitrogen gas flowing at 10 L min^−1^ into a quartz tube reactor maintained at a temperature of 700 °C, where the droplets underwent thermal decomposition to yield the composite microspheres. The physical characteristics of composite microspheres are presented in Figure S2 (Supporting Information). In Figure S2a (Supporting Information), the field‐emission scanning electron microscopy (FE‐SEM) image depicts the 3D spherical morphology of the as‐sprayed powder with an average diameter of 2.5 µm. Additionally, the compact surface of the microspheres clearly indicates the effective confinement of all constituent elements. Further insights are gained from the cross‐sectional image (Figure S2b, Supporting Information), which corroborates that the inner part of the microsphere predominantly comprises Si nanocrystals and Co‐species, while the proportion of PVP‐derived carbon framework gradually increases toward the external parts. The XRD pattern in Figure S2c (Supporting Information) exhibits sharp peaks attributed to the Si, whereas the low‐intensity broad peaks indicate the presence of the CoO phase. The CoO peaks appear, although the process atmosphere is N_2_, due to the reaction of Co^2+^ cations with the O‐containing species present in both the Co‐salt and PVP molecules. The FE‐SEM and XRD results suggest the formation of biphasic Si@CoO multi‐cores, formed due to interaction between Co^2+^ in CoO and O^δ‐^ species present in SiO*_x_* thin film formed over the Si surface. The Raman spectrum in Figure S2d (Supporting Information) reveals the existence of D‐ and G‐bands at 1350 and 1574 cm^−1^, respectively. The relative intensity ratio (RIR; *I*_D_/*I*_G_) of 0.87 suggests that the carbonaceous species within the microspheres primarily exhibit a graphitic nature. This graphitic characteristic is attributed to the catalytic influence of the Co‐species within the microspheres.^[^ ^36^, ^37^, ^38^ ^]^ Notably, the N‐rich organic units present in PVP formed the NGC shell of the powder. Furthermore, the peaks centered at 291 and 510 cm^−1^ are attributed to the Si─Si bonds, whereas the peak situated at 674 cm^−1^ corresponds to the Co─O bonds.^[^ ^39^, ^40^ ^]^ Overall, the above results demonstrate the formation of a PVP‐derived NGC shell comprising Si@CoO multi‐cores. The as‐sprayed Si@CoO/NGC microspheres were the precursor powder for the ex situ PDA‐C coating process.

### 2.1 Optimization of the PDA Coating and Carbonization Conditions

**Figure 1:**
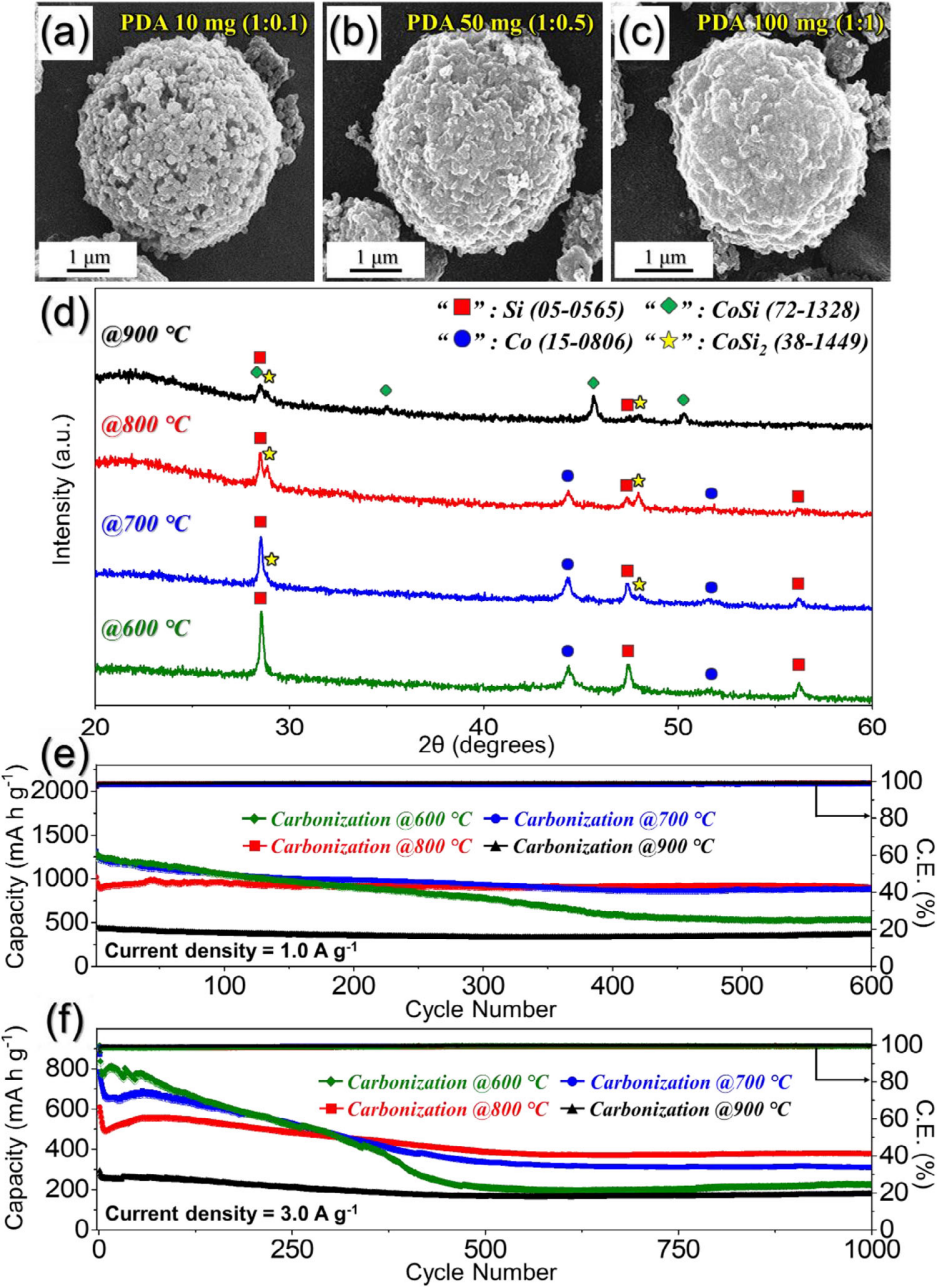
a–c) FE‐SEM images of Si@CoSi_2_‐Co/NGC@PDA‐C microspheres with different mass ratio of PDA obtained after carbonization at 800 °C: a) PDA 10 mg (1:0.1), b) PDA 50 mg (1:0.5), and c) PDA 100 mg (1:1); d) XRD patterns and e,f) electrochemical properties of Si@CoSi_2_‐Co/NGC@PDA‐C microspheres obtained after various carbonization temperature (600–900 °C): e) cycle performances at a current density of 1.0 A g^−1^, and f) cycle performances at a current density of 3.0 A g^−1^.

To ascertain the optimal coating concentration of PDA, different weight ratios between the as‐sprayed Si@CoO/NGC microspheres and dopamine hydrochloride, i.e., 1:0.1, 1:0.5, and 1:1, were taken into consideration. After the coating and carbonization at 800 °C in N_2_ atmosphere, preliminary electrochemical tests were performed to select the appropriate PDA amount. **Figures** **1**a–c and S3 (Supporting Information) display the morphological changes in the as‐sprayed powder coated with different ratios of the PDA amount, along with the rate and cycling performance tests. The FE‐SEM images in Figure 1a–c reveal that the microspheres retained their spherical shape after the PDA coating procedure. However, the external surface of the microsphere exhibited noticeable variations. For instance, when 10 mg (1:0.1 weight ratio) of PDA was used, the microspheres displayed a less dense and compact outer surface (Figure 1a). Conversely, with 100 mg (1:1 weight ratio) of PDA, the outer surface became significantly denser and more compact (Figure 1c). This densification is primarily attributed to the substantial deposition of the PDA‐induced carbon during carbonization. These observations match well with the carbon content determined by the elemental analysis (EA) technique presented in Table S1 (Supporting Information). As observed, the carbon and nitrogen contents increase monotonically with an increase in the PDA amount. To assess the influence of different PDA‐C amounts as a protective shell on electrochemical performance, a series of rate and cycling tests was conducted. The results from the rate performance tests (Figure S3a, Supporting Information) firmly demonstrated that the microspheres treated with 50 mg (1:0.5 weight ratio) of PDA exhibit relatively high discharge capacities, especially at current densities ranging from 0.5 to 3.0 A g^−1^, surpassing the performance of both 10 and 100 mg PDA treated powders. Similarly, the results from the cycling performance tests (Figure S3b, Supporting Information) revealed high discharge capacity values and exceptional stability for the microspheres treated with 50 mg of PDA compared to those treated with 10 and 100 mg of PDA when cycled at a current density of 3.0 A g^−1^. Notably, all anodes exhibited an initial decline in discharge capacity during approximately the first 10 cycles upon the sudden application of the high current density of 3.0 A g^−1^. This initial capacity decrease arises primarily from rapid and incomplete lithiation‐induced structural stresses and localized silicon particle pulverization due to insufficient lithium‐ion diffusion time, causing temporary loss of electrical contact and active material isolation.^[^ ^41^, ^42^, ^43^ ^]^ With continued cycling, however, gradual structural rearrangement occurs, including optimized SEI formation, redistribution and mitigation of internal stresses, and electrochemical reactivation of previously inactive silicon regions. These phenomena collectively result in progressive recovery and subsequent capacity enhancement up to approximately the 50th cycle, confirming a typical electrochemical conditioning or activation process commonly observed in high‐performance Si‐based electrodes under aggressive cycling conditions.^[^ ^44^, ^45^ ^]^ Taken together, these findings indicate that microspheres treated with 50 mg of PDA not only exhibit inherently stable electrochemical performance but also undergo beneficial activation and stabilization processes, leading to enhanced capacity retention even under harsh cycling conditions.

The optimized 50 mg of PDA‐coated Si@CoO/NGC powder finally underwent a carbonization process in N_2_ atmosphere. A series of temperature values ranging from 600–900 °C was selected to optimize the carbo‐reduction sintering condition, favoring the stable Li‐ion storage. Initially, FE‐SEM micrographs and XRD patterns were obtained to analyze the morphological and phase variations as a function of sintering temperature, as shown in Figure S4 (Supporting Information) and Figure 1d. The FE‐SEM micrographs (Figure S4, Supporting Information) reveal that the shape of the samples remained unaltered after sintering at various temperatures. The XRD patterns in Figure 1d indicate that at a low sintering temperature of 600 °C, sharp and intense peaks corresponding to the Si and metallic‐Co phases were observed. The metallic‐Co phase is formed by the carbothermic reduction of CoO species during the carbonization process. However, as the temperature increased to 700 °C, low‐intensity peaks corresponding to the CoSi_2_ phase (2*θ* = 28.8° and 48.0°) began to appear, suggesting possible surface interface reactions between the primary Si particles and secondary metallic‐Co nanoparticles. The CoSi_2_ peaks appeared at the expense of the Si peaks, as evidenced by the reduced intensity of the latter. With a further increase in sintering temperature to 800 °C, the peaks intensities corresponding to CoSi_2_ strengthened, suggesting more interfacial reactions between the Si and Co phases. However, at 900 °C, the intensity corresponding to the Si phase decreased drastically, while the peaks attributed to the metallic‐Co phase disappeared completely. Additionally, a new phase corresponding to the CoSi emerged with considerable peak intensities. Notably, the CoSi phase is extremely unattractive owing to its high resistivity values (80–150 µΩ cm) compared to the CoSi_2_ phase (10–25 µΩ cm), which resulted in lower electrical interconnectivity among Si nanocrystals.^[^ ^46^ ^]^ Based on the XRD results, it appears that a sintering temperature of 800 °C is the optimal value for obtaining a well‐maintained crystal structure composed of Si and considerable proportions of the CoSi_2_ phase, formed due to interfacial reactions between the Si and metallic‐Co nanoparticles. Henceforth, the sample is referred to as “Si@CoSi_2_‐Co/NGC@PDA‐C” microspheres. To determine the effect of the sintering temperature‐derived multiphase crystal structure on electrochemical performance, preliminary cycling property tests were performed at 1.0 and 3.0 A g^−1^ (Figure 1e,f). As anticipated from the XRD results, Si@CoSi_2_‐Co/NGC@PDA‐C microspheres prepared at a carbonization temperature of 800 °C exhibit the highest discharge capacity values and prolonged cycling stability compared to samples prepared at other carbonization temperatures. At a current density of 1.0 A g^−1^ (Figure 1e), the microspheres carbonized at 600 and 700 °C exhibited high initial discharge capacities due to the larger amount of active silicon, and the 700 °C sample, in particular, showed comparable values to the 600 °C sample owing to the additional benefit of enhanced electronic conductivity from partial CoSi_2_ formation. In contrast, the 800 and 900 °C samples exhibited progressively lower initial capacities as the temperature increased, which is due to the increasing formation of electrochemically inactive CoSi_2_ phases that reduce the amount of available active Si. However, in terms of cycling stability, the sample treated at 800 °C outperformed all others. For instance, it achieved a discharge capacity of 900 mAh g^−1^ at the end of the 600th cycle with a capacity retention of ≈88% and an average capacity decay rate of just 0.02% per cycle. Even at a high current density of 3.0 A g^−1^ (Figure 1f), although the initial capacity was lower than the 600 and 700 °C samples, the 800 °C sample retained a high capacity of 380 mAh g^−1^ after 1000 cycles, corresponding to a 70% retention rate. In contrast, the 600 °C sample, despite its initially high capacity, exhibited rapid capacity fading due to insufficient structural stability, while the 900 °C sample demonstrated excellent cycling stability but consistently low capacity throughout the cycles due to excessive CoSi and CoSi_2_ formation and significant loss of active Si. These results are well synchronized with the XRD findings and indicate that 800 °C is the optimized carbonization temperature, providing a favorable balance between the retention of active Si and the formation of a sufficient amount of CoSi_2_ to ensure structural integrity and enhanced kinetically favored redox processes. Additionally, based on the optimized PDA coating and carbonization condition, a comparative study was conducted by substituting the metal precursor with Ni and Fe under identical synthesis conditions. The resulting materials were characterized and electrochemically evaluated, as detailed in Figure S5 (Supporting Information).

### 2.2 Physical Characterizations of the Si@CoSi_2_‐Co/NGC@PDA‐C Microspheres

**Figure 2:**
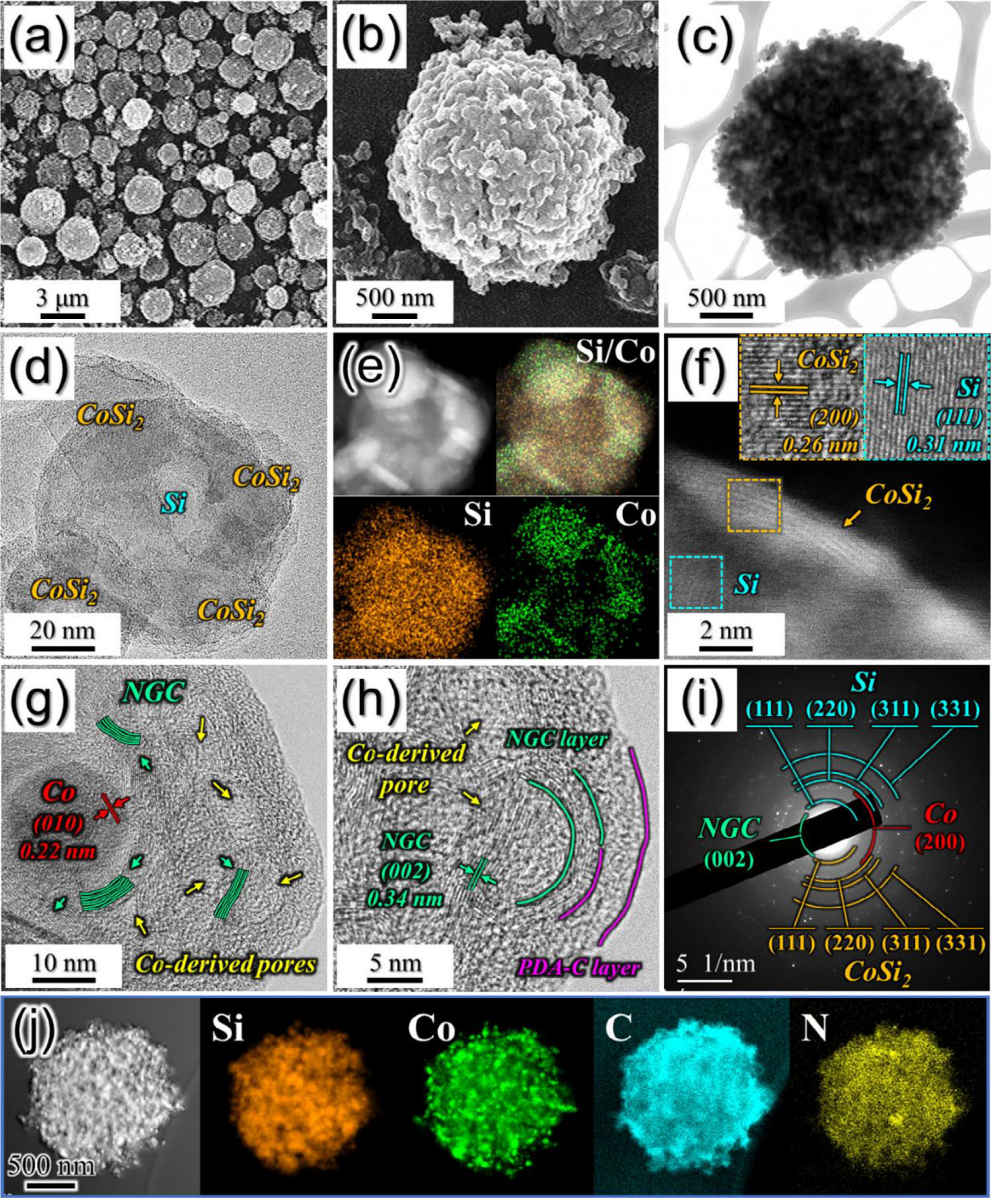
Morphologies, SAED pattern, Raman spectrum, and elemental mapping images of Si@CoSi_2_‐Co/NGC@PDA‐C microspheres; a,b) FE‐SEM images, c,d) TEM images, e) STEM‐elemental mapping images, f) STEM and HR‐TEM images, g,h) TEM images, i) SAED pattern, and j) STEM‐elemental mapping images.

To substantiate the electrochemical performance improvements from the architectural enhancements, a thorough morphological and crystal structure analysis was conducted on the optimized Si@CoSi_2_‐Co/NGC@PDA‐C microspheres. The FE‐SEM micrographs presented in **Figure** **2**a,b demonstrate that the spherical morphology of the resulting powder remains unaltered, even after undergoing a multistep process, underscoring the structural resilience of the nanostructure. Additionally, the formation of a non‐aggregated and uniformly sized microsphere (average *ϕ* = 2.5 µm) is also apparent. A high‐magnification FE‐SEM image (Figure 2b) reveals a predominance of closely packed Si nanocrystals as primary particles surrounded by protective shells of NGC and PDA‐C. Moreover, the voids arising from the interparticle separation are apparently observed due to the shrinkage of the carbon framework during the carbonization process. The TEM image in Figure 2c aligns seamlessly with the FE‐SEM results, further confirming the formation of micron‐sized porous spheres composed of Si and a carbonaceous shell. Furthermore, the TEM image in Figure 2d and the corresponding scanning transmission electron microscopy (STEM) elemental mapping images in Figure 2e unequivocally illustrate a central core composed of Si and enveloped by CoSi_2_ nanoplates. The Si serves as the primary active material, while the CoSi_2_ functions as an inactive and protective buffer material. The CoSi_2_ nanoplates enhance electrical interconnection due to their low bulk resistivity and augment the structural stability and mechanical integrity of the nanostructure owing to their robust thermal and chemical stability. The STEM image in Figure 2f reveals a CoSi_2_ nanoplate with thickness of ≈2 nm on the surface of the Si, and the corresponding HR‐TEM images (Inset of Figure 2f) disclose distinct lattice fringes with clear spacing measurements of 0.26 and 0.31 nm, corresponding to the (200) and (111) diffraction planes of Si and CoSi_2_, respectively. Additionally, the high‐magnification TEM image in Figure 2g unveils the presence of metallic‐Co with a lattice spacing of 0.22 nm corresponding to the (010) plane snugly encapsulated within the NGC shell. The well‐embedded metallic‐Co nanoparticles with intrinsic electrical conductivity of ≈10^5^ S cm^−1^ further reduce the overall electrical resistance of the nanostructure.^[^ ^47^ ^]^ The genesis of the GC is facilitated by the catalytic nature of the metallic‐Co nanoparticles, while the N‐doping arises from the presence of N‐rich organic units found in PVP. Moreover, ≈7 nm pores from the Ostwald ripening of metallic‐Co nanoparticles during the heating process were identified in the NGC shell in Figure 2g. In the more magnified TEM image showing the surface of the microsphere (Figure 2h), a uniform PDA‐C shell with a thickness of ≈5 nm becomes discernible, resulting from the PDA‐derived coating followed by carbonization. These dual protective shells of metallic‐Co embedded porous NGC and PDA‐C serve the dual purpose of facilitating rapid electron transfer from the active materials and safeguarding against the pulverization of Si particles, thus preserving the structural integrity of the anode. Additionally, these protective shells endorse the formation of a stable SEI layer on the surface of Si particles, which is integral to enhanced cycling stability, as elaborated upon later. The selected area electron diffraction (SAED) pattern in Figure 2i impeccably complements these TEM results, displaying well‐resolved diffraction rings attributable to Si, CoSi_2_, Co, and NGC. Collectively, the STEM, HR‐TEM, and SAED results firmly attest to the formation of the multi‐core dual‐shell structure comprising Si@CoSi_2_ multi‐cores and dual protective shells of metallic‐Co nanocrystals (as predicted by XRD in Figure 1d), ensconced within porous NGC and PDA‐C. Furthermore, the STEM‐elemental dot mapping images in Figure 2j clearly illustrate the uniform distribution of Si, Co, C, and N elements, validating the formation of an N‐doped carbon framework comprising Si@CoSi_2_ multi‐cores along with metallic‐Co nanoparticles. Additional high‐resolution STEM‐elemental dot mapping images have been provided in Figure S6 (Supporting Information). The carbon and nitrogen contents within the nanostructure were estimated to be ≈14.3 and 1.3 wt.%, respectively, through the EA analysis in Table S2 (Supporting Information). Rietveld refinement was performed further to quantify the specific contents of the Si, CoSi_2_, and metallic‐Co phase in the prepared nanostructure, and the results are presented in Figure S7 (Supporting Information). The refinement was carried out by considering cubic crystal structures for the Si (*Fd3m* space group), CoSi_2_ (*Fm3m* space group), and metallic‐Co (*Fm3m* space group) phases. As observed, a goodness‐of‐fit (χ^2^) value around 2.5 indicates reliable fitting of the XRD data fitting. Moreover, a phase fraction value of ≈60, 22, and 18 mol% corresponding to Si, CoSi_2_, and metallic‐Co, respectively, aligns with the XRD results in Figure 1d. Based on the EA (Table S2, Supporting Information) and Rietveld refinement (Figure S7, Supporting Information) analysis results, the calculated contents of Si, CoSi_2_, metallic‐Co, and N‐doped carbonaceous materials in Si@CoSi_2_‐Co/NGC@PDA‐C microspheres are 27, 41, 16, and 16 wt.%, respectively.

**Figure 3:**
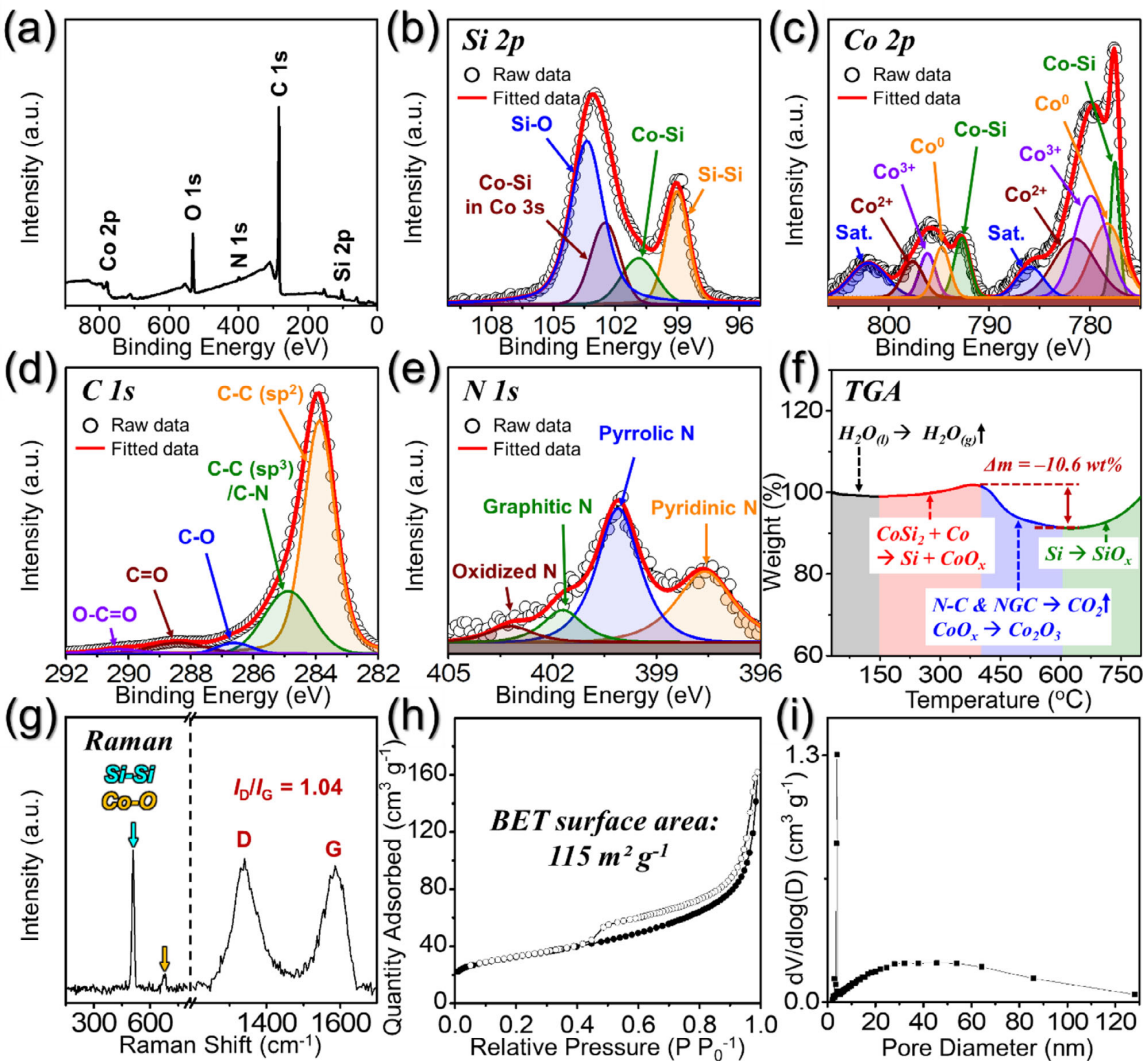
a) Survey spectrum, b) Si 2*p* XPS spectrum, c) Co 2*p* XPS spectrum, d) C 1*s* XPS spectrum, e) N 1*s* XPS spectrum, f) TGA curve, g) Raman spectrum, h) N_2_ adsorption‐desorption isotherms, and i) BJH desorption pore‐size distribution curve of Si@CoSi_2_‐Co/NGC@PDA‐C microsphere.

X‐ray photoelectron spectroscopy (XPS) was employed to analyze the chemical environment and bonding states of the different elements in Si@CoSi_2_‐Co/NGC@PDA‐C microspheres. The survey spectrum in **Figure** **3**a reveals the presence of photoelectron signals corresponding to the atomic orbitals of Si 2*p*, Co 2*p*, C 1*s*, and N 1*s*. The low intensity of Si 2*p* peaks in Si@CoSi_2_‐Co/NGC@PDA‐C microspheres suggests that the Si nanoparticles are well surrounded by the CoSi_2_ nanoplates and the N‐doped carbon shell.^[^ ^48^ ^]^ In addition, the high‐resolution Si 2*p* core‐level XPS spectrum shown in Figure 3b depicts a peak at 98.9 eV, which corresponds to Si─Si chemical bonds and is attributed to the presence of Si in Si@CoSi_2_ multi‐cores.^[^ ^49^, ^50^ ^]^ Fitted peaks observed at 100.8 and 102.5 eV were assigned to Co─Si bonding in Si 2*p* and Co 3*s* atomic orbitals, respectively.^[^ ^49^, ^50^ ^]^ The presence of Co─Si bonds strongly confirms the formation of the CoSi_2_ phase within the nanostructure. Furthermore, the deconvoluted peak at 103.3 eV is assigned to the SiO*_x_* layer, which is formed due to the surface oxidation of the Si nanoparticle.^[^ ^51^ ^]^ The deconvoluted Co 2*p* XPS spectrum in Figure 3c displays well‐resolved and closely spaced peak pairs corresponding to different cobalt species, including metallic‐Co (Co^0^; 778.3/794.7 eV), Co^2+^ (781.5/796.7 eV), and Co^3+^ (779.9/796.1 eV) species.^[^ ^52^, ^53^ ^]^ Additionally, the peak pair centered at 777.5/792.7 eV is associated with Co─Si bonds, further confirming the interaction between Co 2*p* and Si 2*p* orbitals, leading to the formation of CoSi_2_ phase, as depicted by the XRD in Figure 1d.^[^ ^51^ ^]^ Moreover, the two fitted peak pairs located at 785.9/802.0 eV could be assigned to the satellite peaks (marked *“Sat.”*), resulting from electron transitions from bonding to anti‐bonding orbitals.^[^ ^40^ ^]^ The C 1*s* XPS spectra in Figure 3d exhibit five fitted profiles associated with different bonding orbitals, namely, C─C (sp^2^) (283.8 eV), C─C (sp^3^)/C─N (284.8 eV), C─O (286.5 eV), C═O (288.4 eV), and O─C═O (290.3 eV).^[^ ^54^, ^55^, ^56^, ^57^, ^58^ ^]^ Notably, the highest intensity for the C─C (sp^2^) peak strongly validates the presence of a highly conductive graphitic carbon shell formed through the catalytic influence of metallic‐Co in the nanostructure.^[^ ^59^, ^60^, ^61^ ^]^ Additionally, the appearance of the C─C (sp^3^)/C─N fitted peak confirms the presence of N‐doping within the carbon shell.^[^ ^62^, ^63^, ^64^ ^]^ This observation is consistent with the deconvoluted N 1*s* XPS spectrum shown in Figure 3e, which exhibits four fitted photoelectron peaks associated with different nitrogen environments, including pyridinic N (397.6 eV), pyrrolic N (400.0 eV), graphitic N (401.6 eV), and oxidized N (403.2 eV).^[^ ^65^, ^66^ ^]^ The high‐resolution C 1*s* and N 1*s* spectra firmly validate the formation of an N‐doped graphitic carbon shell within the nanostructure characterized by an abundance of surface defects primarily induced by high levels of N‐doping. The observations are well supported by the EA results in Table S2 (Supporting Information). The carbon content in the Si@CoSi_2_‐Co/NGC@PDA‐C microspheres was also quantified using TG analysis (Figure 3f) and was ≈10.6 wt.%, which differs slightly different from the EA results in Table S2 (Supporting Information). This difference is due to the weight gain from the oxidation of the Co‐species offsetting the weight loss from the carbon combustion in Si@CoSi_2_‐Co/NGC@PDA‐C microspheres. Moreover, the crystalline attributes of the graphitic carbon shell within the microspheres were further investigated using Raman spectroscopy, as depicted in Figure 3g. The characteristics D‐ and G‐band signatures manifest at 1340 and 1585 cm^−1^, respectively, with a relative intensity ratio (*I_D_/I_G_*) value of 1.04, affirming the graphitic nature of the carbonaceous species in the nanostructure.^[^ ^37^, ^38^ ^]^ However, a higher *I_D_/I_G_* value of Si@CoSi_2_‐Co/NGC@PDA‐C microspheres compared to the as‐sprayed powder (Figure S2d, Supporting Information) is mainly due to the PDA‐derived N‐doped carbon shell and the formation of Co‐derived pores. Additionally, the peaks situated at 510 cm^−1^ and 680 cm^−1^ also represent Si─Si and Co─O bonds, respectively.^[^ ^39^, ^40^ ^]^ The appearance of the Co─O bond is due to the surface oxidation of metallic‐Co nanoparticles exposed to air. Furthermore, the Si@CoSi_2_‐Co/NGC@PDA‐C microspheres exhibit a high BET surface area of 115 m^2^ g^−1^ (Figure 3h), authenticating the presence of a porous structure. The porosity is primarily induced by mesopores in the form of voids and micropores resulting from N‐doping‐induced defects, as evidenced by the Barrett–Joyner–Halenda (BJH) pore size distribution curve shown in Figure 3i. Additionally, the sharp peak located at ≈4 nm is attributed to the tensile‐strength effect,^[^ ^67^ ^]^ and pore distribution of <10 nm is observed originating from the Ostwald ripening of metallic‐Co nanoparticles, which is well synchronized with the TEM results (Figure 2g). The porous structure facilitates the smooth diffusion of charged species by reducing the effective diffusion length while simultaneously accommodating the volume fluctuation, thereby improving overall electrochemical performance.

**Scheme 1:**
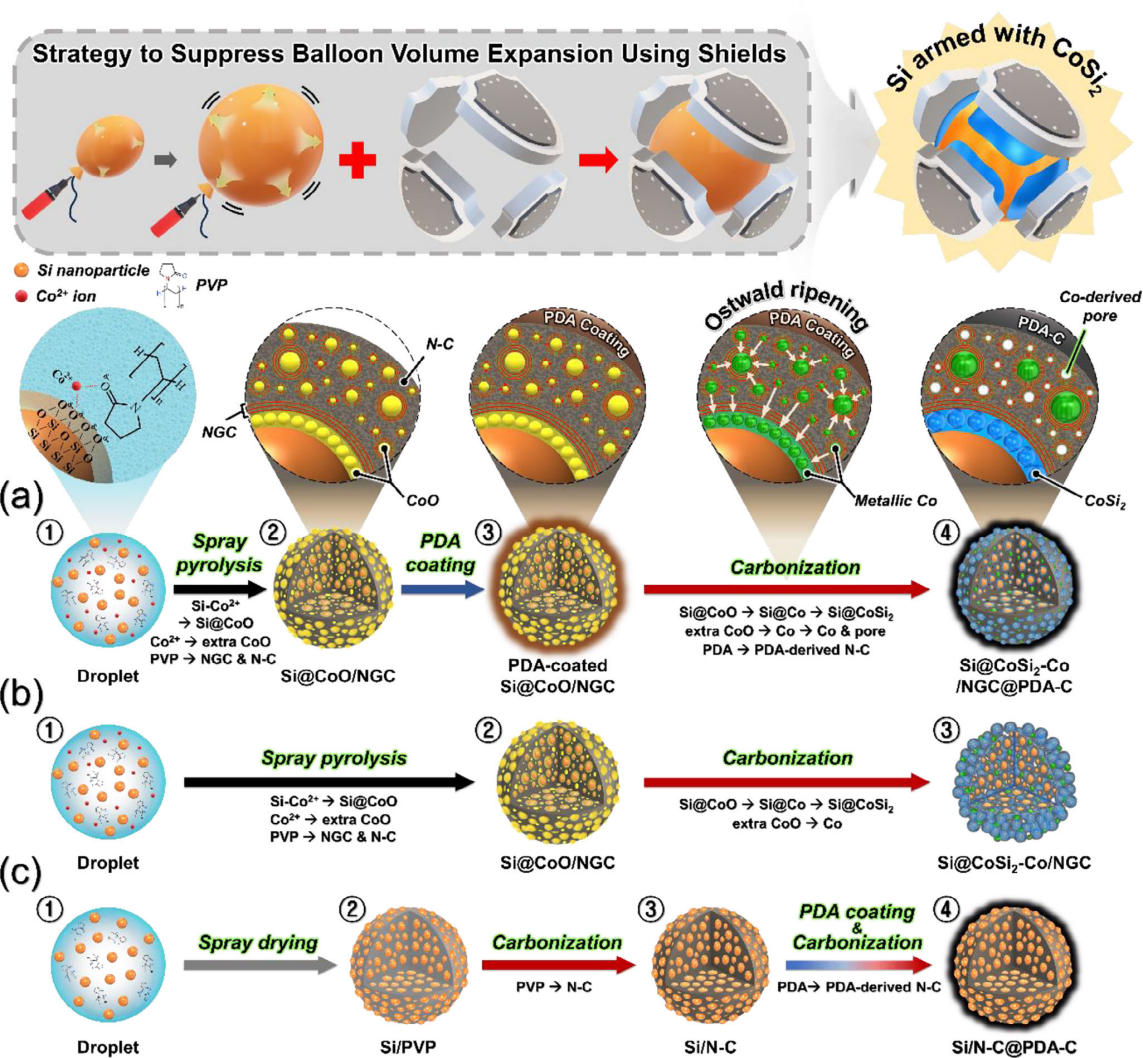
Schematic representation of the formation mechanism of a) Si@CoSi_2_‐Co/NGC@PDA‐C, b) Si@CoSi_2_‐Co/NGC, and c) Si/N‐C@PDA‐C microspheres.

Based on the aforementioned characterization results and discussion of the nanostructures obtained at each process, the detailed formation mechanism of Si@CoSi_2_‐Co/NGC@PDA‐C microspheres is delineated in **Scheme** **1**a. The aqueous droplet, generated by the nebulizer, comprises a homogeneously dispersed mixture of Si nanoparticles, Co‐salt, and PVP as carbon sources. The uniformity of the spray solution is notably due to interactions among the charged species in Si (O^δ‒^ in the form of SiO*_x_* layer on the surface), Co‐salt (Co^2+^ ions), and PVP (amide ring), as depicted in Scheme 1a‐①. To investigate the interaction of the various components in the spray solution in detail, digital images of the solutions at different time intervals are vividly shown in Figure S8 (Supporting Information). As observed, adding Co‐salt into the solution containing well‐dispersed Si nanoparticles induced phase sedimentation of the solution after 12 h. However, after adding amphiphilic PVP to the Si/Co‐salt dispersion, a uniform colloidal suspension was obtained mainly due to the formation of Si─Co^2+^–PVP complexes, resulting in a sedimentation‐free homogeneous spray solution. During spray pyrolysis with Si/Co‐salt/PVP solution, the Co^2+^ ions surrounding the Si nanocrystals transformed to CoO species, thus forming biphasic Si@CoO multi‐cores. Simultaneously, the PVP was converted into NGC and nitrogen‐doped carbonaceous (N─C) species depending on the proximity of Co‐species, which act as catalytic sites. Notably, the reddish upper part in the solution containing only Si and Co‐salt after 12 h (Figure S8, Supporting Information) is configured with an excess of Co^2+^ ions. These excess Co^2+^ ions become embedded within the PVP shell and transition to CoO nanoparticles. Therefore, the microspheres comprising Si@CoO multi‐cores and additional CoO nanocrystals well embedded within the NGC shell (Si@CoO/NGC) are successfully synthesized by the spray pyrolysis process (Scheme 1a‐②). Furthermore, the as‐sprayed Si@CoO/NGC microspheres undergo a solution‐based PDA‐coating process followed by carbonization at a temperature of 800 °C for 5 h in N_2_ atmosphere (Scheme 1a‐③ and ④). During carbonization, the extra CoO species were further reduced to Co‐nuclei by carbothermic reduction, which subsequently underwent Ostwald ripening to form larger metallic‐Co nanoparticles within the NGC shell. This transformation from small Co‐nuclei to larger nanoparticles resulted in Co‐induced pores within the carbon shell. The metallic‐Co embedded porous NGC shell provides numerous conductive paths for rapid electron transfer and enhances the electrolyte wettability of microspheres, facilitating the smooth diffusion of charged species. Additionally, the CoO surrounding the Si nanoparticles is converted into inactive CoSi_2_ nanoplates via interfacial reactions. The inactive CoSi_2_ is believed to enhance electrical interconnection, structural stability, and mechanical integrity of the nanostructure. Furthermore, the PDA coating transformed into a uniform nitrogen‐doped PDA‐C. The carbonization step eventually resulted in the formation of a dual shell‐protected Si@CoSi_2_‐Co/NGC@PDA‐C microspheres. Additionally, two comparison samples were prepared to further validate the structural integrity of Si@CoSi_2_‐Co/NGC@PDA‐C microspheres. First, Scheme 1b illustrates the formation mechanism of microspheres without a PDA‐C protective shell, namely “Si@CoSi_2_‐Co/NGC” microspheres, using identical as‐sprayed powders followed by carbonization at 800 °C for 5 h in N_2_ atmosphere. In this case, the formation of a microsphere comprising sparsely connected Si@CoSi_2_ nanocrystals and metallic‐Co nanoparticles coated with trace amounts of NGCs was achieved. Second, Scheme 1c describes the detailed synthesis process of microspheres obtained without a CoSi_2_ nanoplate and metallic‐Co embedded porous NGC shell via the spray drying process (Scheme S1b, Supporting Information), followed by carbonization and finally PDA‐coating, trailed by an additional heat‐treatment in N_2_ atmosphere. The finally obtained sample is abbreviated as “Si/N‐C@PDA‐C” microspheres. As discussed above, Si@CoSi_2_‐Co/NGC microspheres without the PDA‐C protective shell and Si/N‐C@PDA‐C microspheres without both CoSi_2_ nanoplates and NGC shell are synthesized as a comparison sample. Additionally, the physical characterization of Si@CoSi_2_‐Co/NGC and Si/N‐C@PDA‐C microspheres is depicted in Figures S9 and S10 (Supporting Information), respectively, along with the relevant discussion.

### 2.3 Half‐Cell Electrochemical Performance of the Nanostructures

**Figure 4:**
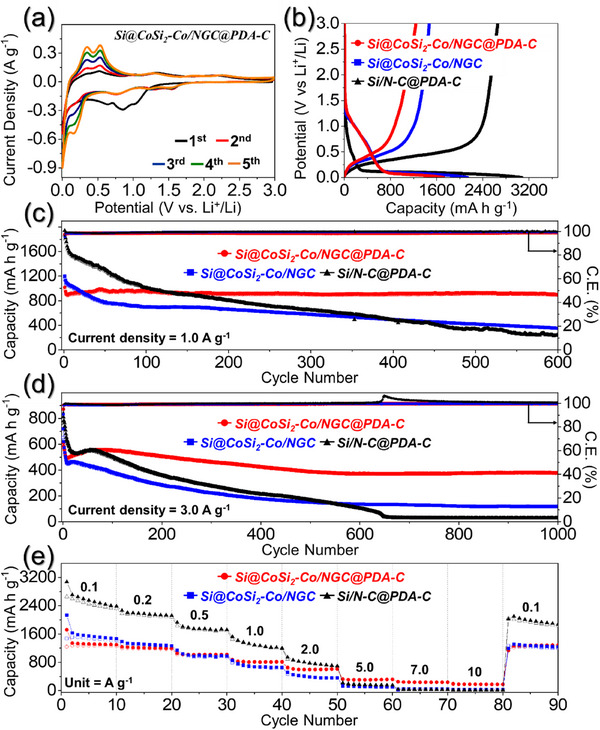
Electrochemical properties of Si@CoSi_2_‐Co/NGC@PDA‐C, Si@CoSi_2_‐Co/NGC, Si/N‐C@PDA‐C anodes; a) CV curves of Si@CoSi_2_‐Co/NGC@PDA‐C, b) initial discharge/charge curves for prepared microspheres at a constant current density of 0.1 A g^−1^, c) cycle performance at a current density of 1.0 A g^−1^, d) cycle performance at a current density of 3.0 A g^−1^, and e) rate capability at various current densities ranging from 0.1 to 10 A g^−1^.

To authenticate the improved nanostructure characteristics of Si@CoSi_2_‐Co/NGC@PDA‐C microspheres over Si@CoSi_2_‐Co/NGC and Si/N‐C@PDA‐C microspheres, Li‐ion storage properties were evaluated using CR2032 coin cell configurations. Initially, the cells utilizing the prepared anodes were subjected to cyclic voltammetry (CV) measurements at a voltage scan rate of 0.1 mV s^−1^ in the voltage range of 0.001–3.0 V (vs Li^+^/Li), as shown in **Figures** **4**a and S11 (Supporting Information). Five cycles were plotted for all anodes to examine the associated redox processes. During the first cathodic scan of Si@CoSi_2_‐Co/NGC@PDA‐C anode (Figure 4a), two peaks at 1.0 and 0.85 V emerged, accounting for the reduction of trace CoO species associated with surface oxidation of metallic‐Co nanocrystals.^[^ ^5^, ^50^ ^]^ Moreover, a peak located at 0.75 V corresponds to the formation of an SEI layer on the electrode surface.^[^ ^68^ ^]^ Similar trends were observed for Si@CoSi_2_‐Co/NGC microspheres (Figure S11a, Supporting Information), demonstrating the reduction of CoO species to metallic‐Co around 1.02 V. The appearance of a less distinct peak at 0.77 V is attributed to the formation of metallic‐Co and the SEI layer. However, it's worth noting that the peak intensity was moderately low, suggesting the formation of a thin and unstable SEI layer on the electrode surface. In contrast, the Si/N‐C@PDA‐C (Figure S11b, Supporting Information) anode did not show any peaks related to Co‐species due to their absence. However, Si/N‐C@PDA‐C without a conductive CoSi_2_ exhibits weak SEI formation at a peak of 0.77 V (marked as ^*^). This observation underscores the formation of a highly stable SEI layer through the synergistic effects between conductive CoSi_2_ and the metallic‐Co embedded porous NGC protective shell for Si@CoSi_2_‐Co/NGC@PDA‐C. Furthermore, all anodes exhibited a distinctive cathodic peak at around 0.16 V, which was attributed to the formation of an amorphous Li*_x_* Si phase.^[^ ^69^ ^]^ A sharp cathodic peak at 0.01 V was associated with the transition of the amorphous Li*_x_* Si phase into a crystalline Li_15_Si_4_ phase and the insertion of Li‐ions into the carbonaceous shells.^[^ ^50^ ^]^ During the first reverse scan, the broad oxidation region spanning from 0.14 to 0.60 V in all anodes indicates the transformation of crystalline Li_15_Si_4_ phases to an amorphous Si (Li_15_Si_4_ → Li*_x_* Si → Si) and the extraction of Li‐ions from the carbon shells. Moreover, the redox intensities increased monotonically during the initial five cycles for all anodes, mainly due to the gradually improved redox kinetics of Si during the Li‐alloying/dealloying processes.^[^ ^70^, ^71^ ^]^ Notably, the difference in redox peak intensities among the electrodes can be attributed to the relative Si content in each sample. Si@CoSi_2_‐Co/NGC, lacking a PDA‐C layer, contains a higher Si content than Si@CoSi_2_‐Co/NGC@PDA‐C, while Si/N‐C@PDA‐C includes no secondary Co‐based phase, resulting in the highest active Si content. Consequently, these two electrodes show stronger CV peak intensities compared to Si@CoSi_2_‐Co/NGC@PDA‐C, where the additional carbon coating and Co‐based phases reduce the relative Si proportion. Additionally, the low‐intensity redox pairs for Si@CoSi_2_‐Co/NGC@PDA‐C, which appeared in the voltage range of 0.8–2.3 V, are associated with the redox reactions corresponding to the trace level of CoO. Similar redox pairs also occur for Si@CoSi_2_‐Co/NGC microspheres during consecutive cycles. Notably, the CoSi_2_ phase is electrochemically inactive and does not contribute to the CV responses. Overall, the CV results demonstrate the formation of a stable SEI layer for the Si@CoSi_2_‐Co/NGC@PDA‐C anode and predict that the electrode performance can be assessed exclusively based on the Si phase and trivial CoO species within the ternary composite.

To validate the findings from the CV measurements, initial Galvanostatic discharge/charge profiles were obtained for the prepared microspheres, conducted at a current density of 0.1 A g^−1^, as depicted in Figure 4b. The discharge/charge plateaus corresponding to the different redox processes are consistent with the peak positions observed in the CV curves. For instance, corresponding to Si@CoSi_2_‐Co/NGC@PDA‐C and Si@CoSi_2_‐Co/NGC microspheres, the discharge voltage profile initially displayed a gradual decrease until ≈0.12 V. This decrease was due to SEI formation and reduction of surface oxidized CoO species to metallic‐Co and a relatively flat voltage plateau, which is related to the formation of Li_15_Si_4_ phase and the insertion of Li‐ions into the carbon shells. The charge voltage profile closely mirrored the discharge, indicating the transformation of Li_15_Si_4_ into an amorphous Si state and the extraction of Li‐ions. In contrast, the Si/N‐C@PDA‐C anode exhibited a steeper slope until 0.15 V, trailed by a long, distinct voltage plateau corresponding to the Li─Si alloying reaction. Similarly, during the reverse scan, the charge plateau corresponding to the dealloying reaction of Si was less steep and more extended, suggesting the Si content in the Si/N‐C@PDA‐C anode is higher than in the two samples due to the absence of Co‐species. The initial discharge/charge capacities for Si@CoSi_2_‐Co/NGC@PDA‐C, Si@CoSi_2_‐Co/NGC, and Si/N‐C@PDA‐C microspheres were 1725/1239, 2139/1475, and 3076/2660 mAh g^−1^, respectively, resulting in initial Coulombic efficiency (ICEs) of 72, 69, and 86%, respectively. The capacity values are proportional to the Si phase content, with the Si/N‐C@PDA‐C anode without Co‐species exhibiting the highest initial discharge capacity, followed by Si@CoSi_2_‐Co/NGC, and finally the Si@CoSi_2_‐Co/NGC@PDA‐C anode. The lower ICEs for Si@CoSi_2_‐Co/NGC@PDA‐C compared to Si/N‐C@PDA‐C microspheres can be attributed to the presence of surface oxidized CoO species, resulting in a relatively higher initial irreversible capacity loss. However, despite both Si@CoSi_2_‐Co/NGC@PDA‐C and Si@CoSi_2_‐Co/NGC containing surface oxidized CoO species, and although Si@CoSi_2_‐Co/NGC@PDA‐C has a higher specific surface area, the higher ICE of Si@CoSi_2_‐Co/NGC@PDA‐C compared to Si@CoSi_2_‐Co/NGC microspheres results from the presence of highly conductive carbonaceous dual‐shells comprising nanosized‐metallic‐Co embedded NGC and PDA‐C. Moreover, it is noteworthy that the Coulombic efficiency (CE) of Si@CoSi_2_‐Co/NGC@PDA‐C anode immediately increased to 98% after four cycles of activation and maintained high CE values (>99%) throughout the cycling process, indicating the formation of a stable SEI layer. Overall, both the CV measurements and the initial discharge/charge voltage profiles envisaged improved electrochemical performance derived from the enhanced nanostructure characteristics of Si@CoSi_2_‐Co/NGC@PDA‐C microspheres.

To validate the CV and initial voltage profile results, cycling stability tests were also conducted for Si@CoSi_2_‐Co/NGC@PDA‐C, Si@CoSi_2_‐Co/NGC, and Si/N‐C@PDA‐C microspheres. The cycling performance of different prepared anodes possessing different microstructural and crystal structure properties was investigated under current densities of 1.0 A g^−1^ (Figure 4c) and 3.0 A g^−1^ (Figure 4d). Notably, before evaluating the cycling properties at 1.0 A g^−1^, an activation process was performed at 0.1 and 0.5 A g^−1^ for two consecutive cycles. As observed, the Si@CoSi_2_‐Co/NGC@PDA‐C anode demonstrated remarkable stability over 600 cycles, delivering a reversible discharge capacity of 900 mAh g^−1^. This signifies an impressive capacity retention of 88% with an average decay rate of 0.02% per cycle. While the cell employing Si@CoSi_2_‐Co/NGC anode initially exhibited relatively high discharge capacity values during the first few tens of cycles, the capacity steadily declined as cycling continued. By the end of the 600th cycle, the cell's discharge capacity had dwindled to 354 mAh g^−1^, representing a capacity retention of just 29% (with an average decay rate of 0.11% per cycle). Similar trends were observed for the Si/N‐C@PDA‐C anode, which exhibited a high discharge capacity of 1942 mAh g^−1^ during the first cycle, steadily decreasing through 600 cycles. Subsequently, the cell delivered a low discharge capacity of just 241 mAh g^−1^ (12% retention) at the end of the 600th cycle. It's worth noting that the high initial discharge capacity of the Si/N‐C@PDA‐C anode is due to the high active material content in the form of Si. The excellent cycling performance of the cell with Si@CoSi_2_‐Co/NGC@PDA‐C anode can be attributed to a synergistic effect between the multicomponents. For instance, the inactive CoSi_2_ acts as a buffer material with low bulk resistivity and high chemical stability, enhancing the electrical interconnection and overall mechanical integrity of the nanostructure. Furthermore, the metallic‐Co embedded porous NGC protective shell ensures rapid electron transfer by providing primary transport pathways due to its high conductivity characteristics, while improving the structural integrity of the nanostructure by partially restraining the volume expansion of the Si. Finally, an additional PDA‐C shell on the surface of the microspheres provides secondary transport pathways for charged species while mitigating the volume expansion in the nanostructure, thus preventing complete detachment or pulverization. This enhances the structural integrity of the electrode, resulting in improved electrochemical performance and long‐term stable cycling. Similar trends were observed for all anodes when cycling was performed at a higher current density of 3.0 A g^−1^ (Figure 4d). The cell equipped with a Si@CoSi_2_‐Co/NGC@PDA‐C anode exhibited a high reversible discharge capacity of 380 mAh g^−1^ (70% capacity retention with an average capacity loss of 0.03% per cycle) at the end of the 1000th cycle. In comparison, the Si@CoSi_2_‐Co/NGC (119 mAh g^−1^, 16% retention, and 0.08% decay rate) and Si/N‐C@PDA‐C (27 mAh g^−1^, 3% retention, 0.09% decay rate) anodes showed lower retention and high‐capacity decay rates. Furthermore, the Si@CoSi_2_‐Co/NGC@PDA‐C anode demonstrated a high CE of 99.5%, indicating highly reversible redox processes within the anode. Overall, the cycling results validate that the rationally designed nanostructure with precisely coated protective shells forms a robust conductive architecture capable of withstanding the fundamental challenges faced by Si‐based anodes. Such architecture provides remarkable cycling stability and supports reversible electrochemical processes.

To further confirm the structural merits, the rate performances of the prepared microspheres were assessed at different current densities ranging from 0.1 to 10 A g^−1^, as presented in Figure 4e. As is evident, the cell featuring the Si/N‐C@PDA‐C anode exhibited exceptionally high discharge capacities at low (≤0.5 A g^−1^) and moderate current densities (1.0 and 2.0 A g^−1^) due to the high active material in the Si anode. However, the discharge capacities dropped significantly at higher current densities (> 2.0 A g^−1^), suggesting sluggish Li‐ion kinetics. In contrast, the Si@CoSi_2_‐Co/NGC@PDA‐C and Si@CoSi_2_‐Co/NGC microspheres delivered relatively low discharge capacities up to 2.0 A g^−1^ but achieved considerable values at higher current densities (5.0, 7.0, and 10 A g^−1^). Specifically, the discharge capacity values for the Si@CoSi_2_‐Co/NGC@PDA‐C anode were 1299, 1200, 1021, 817, 611, 315, 236, and 177 mAh g^−1^ at current densities of 0.1, 0.2, 0.5, 1.0, 2.0, 5.0, 7.0, and 10 A g^−1^, respectively. In contrast, the capacity values for the Si@CoSi_2_‐Co/NGC and Si/N‐C@PDA‐C anodes were 1468/2386, 1267/2120, 974/1720, 657/1201, 364/697, 103/153, 44/27, and 27/13 mAh g^−1^, respectively, at the same current density values. These results firmly validate that the structural advantages of the Si@CoSi_2_‐Co/NGC@PDA‐C microspheres maintain the robustness of the architecture, resulting in kinetically favored redox kinetics by allowing easy penetration of the electrolyte and improving the diffusion rate of Li‐ions. Notably, the electrochemical performance achieved with the Si@CoSi_2_‐Co/NGC@PDA‐C anode is even better or comparable to other Si/carbon and Si/Me*_x_* Si*_y_* (Me = Co, Ti, Fe, Cu) composite‐based anode materials, as summarized in Table S3 (Supporting Information).

**Figure 5:**
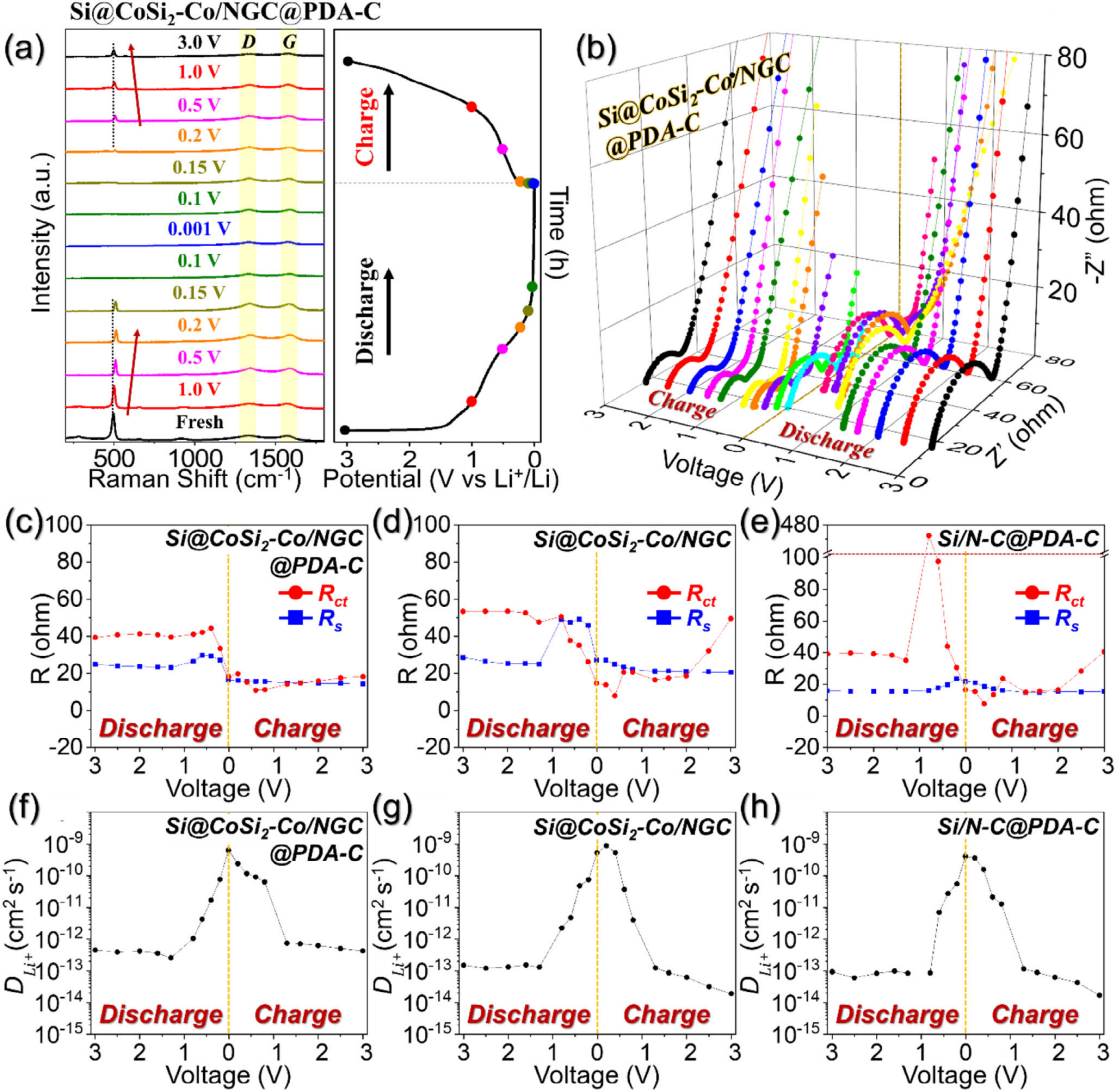
a) Ex situ Raman spectra and b) 3D plots of in situ EIS profiles over a voltage range of 3.0–0.001 V for the Si@CoSi_2_‐Co/NGC@PDA‐C anode during the initial cycle at 0.1 A g^−1^; c–e) evolution of charge‐transfer resistance (*R*_ct_) and solution resistance (*R*_s_) during lithiation/delithiation for each sample; f–h) corresponding Li‐ion diffusion coefficient ($D_{Li^{+}}$) profiles calculated from in situ EIS for each sample.

To elucidate the synergistic structural advantages and underlying Li‐ion storage kinetics of the designed multi‐core–dual‐shell architecture, comprehensive ex situ Raman and in situ EIS analysis were conducted during the initial discharge/charge cycle (**Figure** **5**). The ex situ Raman spectra obtained at various voltage intervals clearly illustrate the structural evolution of Si within the Si@CoSi_2_‐Co/NGC@PDA‐C anode (Figure 5a). Specifically, in the fresh electrode, distinct vibrational peaks corresponding to carbonaceous D‐ and G‐bands (1344 and 1585 cm^−1^, respectively) and the crystalline Si─Si bond at 498 cm^−1^ were observed. Upon initial lithiation down to 0.2 V, the Si─Si peak gradually shifted toward lower frequencies (red shift), indicating progressive lithiation‐induced bond weakening and transition to amorphous Si. Eventually, complete amorphization and formation of Li─Si alloy phases was confirmed by the disappearance of the Si─Si peak below 0.1 V. During subsequent charging, this peak reappeared at ≈0.2 V and gradually shifted back (blue shift) toward its initial position at 498 cm^−1^ at 3.0 V, verifying partial recrystallization and de‐alloying processes and confirming highly reversible Li‐ion storage behavior. Similar reversible lithiation/delithiation trends were also observed for Si@CoSi_2_‐Co/NGC anode, while notably delayed reappearance and weaker Si─Si peaks for the Si/N‐C@PDA‐C anode confirmed sluggish de‐lithiation kinetics, likely due to the absence of the stabilizing conductive CoSi_2_ phase (Figure S12a,b, Supporting Information). Complementary in situ EIS measurements further illuminated the electrochemical kinetics differences among the anodes (Figure 5b–h; Figure S12c,d, Supporting Information). The Si@CoSi_2_‐Co/NGC@PDA‐C anode maintained stable and low values of charge transfer resistance (*R*_ct_) and solution resistance (*R*_s_) down to ≈1.0 V during initial discharge. Below this voltage, *R*_s_ gradually increased due to the electrolyte decomposition and subsequent SEI formation, while concurrently, *R*_ct_ values decreased owing to improved interfacial contact facilitated by the forming SEI. Importantly, the Li‐ion diffusion coefficient ($D_{Li^{+}}$) continuously increased below 1.0 V due to enhanced lithium diffusion within the amorphous Li─Si phases formed upon lithiation. During the charge, $D_{Li^{+}}$ gradually reverted to its initial values, accompanied by stable *R*_ct_ and *R*_s_, emphasizing robust SEI stability and highly reversible electrochemical reactions. By contrast, Si@CoSi_2_‐Co/NGC showed significant *R*_s_ increases, primarily driven by electrolyte parasitic reactions with exposed metallic‐Co species as shown in the TEM image (Figure S9e, Supporting Information). Moreover, the Si/N‐C@PDA‐C anode displayed dramatic *R*_ct_ growth (reaching ≈475 Ω) around 1.0 V during charging, directly resulting from extensive amorphization‐induced Si exposure that intensified electrolyte interactions, exacerbating interfacial resistance. Thus, the CoSi_2_ nanoplate shells critically functioned as effective protective buffers, significantly suppressing excessive SEI formation and limiting *R*_ct_ build‐up. These combined ex situ Raman and in situ EIS findings decisively demonstrate the synergistic structural stability and enhanced Li‐ion transport kinetics uniquely provided by the CoSi_2_–dual carbon shell architecture of the Si@CoSi_2_‐Co/NGC@PDA‐C anode.

To further elucidate the mechanism governing Li‐ion reaction kinetics within the Si@CoSi_2_‐Co/NGC@PDA‐C anode material, the CV curves were acquired across a range of sweep rates, spanning from 0.1 to 1.2 mV s^−1^, as depicted in Figures S13 and S14 (Supporting Information). Diffusion‐ and capacitive‐controlled reactions were differentiated by plotting the peak current (*i*) values obtained during the electrochemical reactions against the voltage sweep rates (*v*) using the following relation:^[^ ^72^ ^]^

(1) $$i=av^{b}$$

(2) $$\log\left(i\right)=blog\left(v\right)+\log\left(a\right)$$

The extent to which diffusion‐ and capacitive‐controlled processes contributed to the reactions is assessed by the parameters *a* and *b*. Specifically, a value of *b* approaching 1.0 indicates that the redox process is predominantly capacitive or surface‐controlled, while a value of *b* tending toward 0.5 suggests a primarily diffusion‐controlled process. The *b*‐values were computed by establishing the relationship between the log (*i*) versus log (*v*) for the peaks displayed in Figure S13b,f,j (Supporting Information), corresponding to the primary redox reactions. In Si@CoSi_2_‐Co/NGC@PDA‐C anode, the *b*‐values for peaks 1 and 2 were 0.71 and 0.77, respectively (Figure S13b, Supporting Information). These values fall between 0.5 and 1.0, indicating a balance between diffusion‐ and capacitive‐controlled electrochemical processes. Notably, the presence of the PDA‐C shell induces higher porosity inside the nanostructure, ultimately leading to considerable diffusion and capacitive reactions. Likewise, Si/N‐C@PDA‐C displays similar *b*‐values (Figure S13j, Supporting Information), thus confirming that similar redox and surface processes occur inside the cell. In contrast, the reaction kinetics observed for Si@CoSi_2_‐Co/NGC (Figure S13e,f, Supporting Information) displayed the lowest *b*‐values, signifying a strong preference for the diffusion‐controlled phenomenon. This observation is attributed to the insufficiency of the carbonaceous shell, which resulted in the lowest surface area, thus favoring a more significant diffusion process. Although the trivial surface reaction still occurs over the microsphere surface, resulting in capacitive processes, it is to a lower extent than the other two samples. To quantitatively assess the contribution of diffusion‐ and capacitive‐controlled processes, each process was isolated using the area under the total charge storage curve, as described by the following equation:^[^ ^40^ ^]^

(3) $$i=k_{1}v+k_{2}v^{1/2}$$

In this equation, the first term on the right side (*k*_1_ *v*) represents the current generated by the capacitive reaction, while the second term (*k*_2_ *v* ^1/2^) signifies the current resulting from diffusion‐controlled reactions. The proportions of capacitive reactions relative to the total current for Si@CoSi_2_‐Co/NGC@PDA‐C, Si@CoSi_2_‐Co/NGC, and Si/N‐C@PDA‐C microspheres at a scan rate of 0.1 mV s^−1^ were found to be 38, 25, and 39%, respectively (Figure S13c,g,k, Supporting Information). These results align with the *b*‐values discussed above. The measurement of capacitive contribution at different scan rates ranging from 0.1 to 1.2 mV s^−1^ is depicted in Figure S13d,h,l (Supporting Information). The higher percentage of capacitive reaction for Si@CoSi_2_‐Co/NGC@PDA‐C and Si/N‐C@PDA‐C anodes firmly supports better Li‐ion reaction kinetics than Si@CoSi_2_‐Co/NGC. Furthermore, the Randles–Sevick equation (mentioned below) was used to provide deeper insights into the electrochemical kinetics of diffusion‐controlled reactions within the cells, and the results are summarized in Figure S14 (Supporting Information).^[^ ^62^ ^]^

(4) $$I_{p}=2.69\times 10^{5}n^{1.5}AD_{Li^{+}}^{0.5}C_{\mathrm{Li}}\nu^{0.5}$$

where *I_p_* represents the redox peak current, *n* is the number of electrons involved (*n* = 3.75), *A* is the electrode surface area (cm^2^), *C_Li_* is the concentration (mol L^−1^) of Li‐ion, and *v* is the voltage scan rate (V s^−1^). The *I_p_* versus *v^0.5^* curves in Figure S14d (Supporting Information) revealed that Si@CoSi_2_‐Co/NGC@PDA‐C exhibits the highest diffusion characteristics compared to Si@CoSi_2_‐Co/NGC (Figure S14e, Supporting Information) and Si/N‐C@PDA‐C (Figure S14f, Supporting Information). The average $D_{Li^{+}}$ values present in Figure S14g (Supporting Information) vividly indicate that the $D_{Li^{+}}$ values of 4.8 × 10^−10^ cm^2^ s^−1^ for Si@CoSi_2_‐Co/NGC@PDA‐C anode are 1.2 and 1.5 times higher than the Si@CoSi_2_‐Co/NGC (3.8 × 10^−10^ cm^2^ s^−1^) and Si/N‐C@PDA‐C (3.1 × 10^−10^ cm^2^ s^−1^), respectively. These results again validate that the synergy structure between various components in Si@CoSi_2_‐Co/NGC@PDA‐C anode guarantees kinetically favored reaction kinetics, which substantially improved the electrochemical performance.

**Figure 6:**
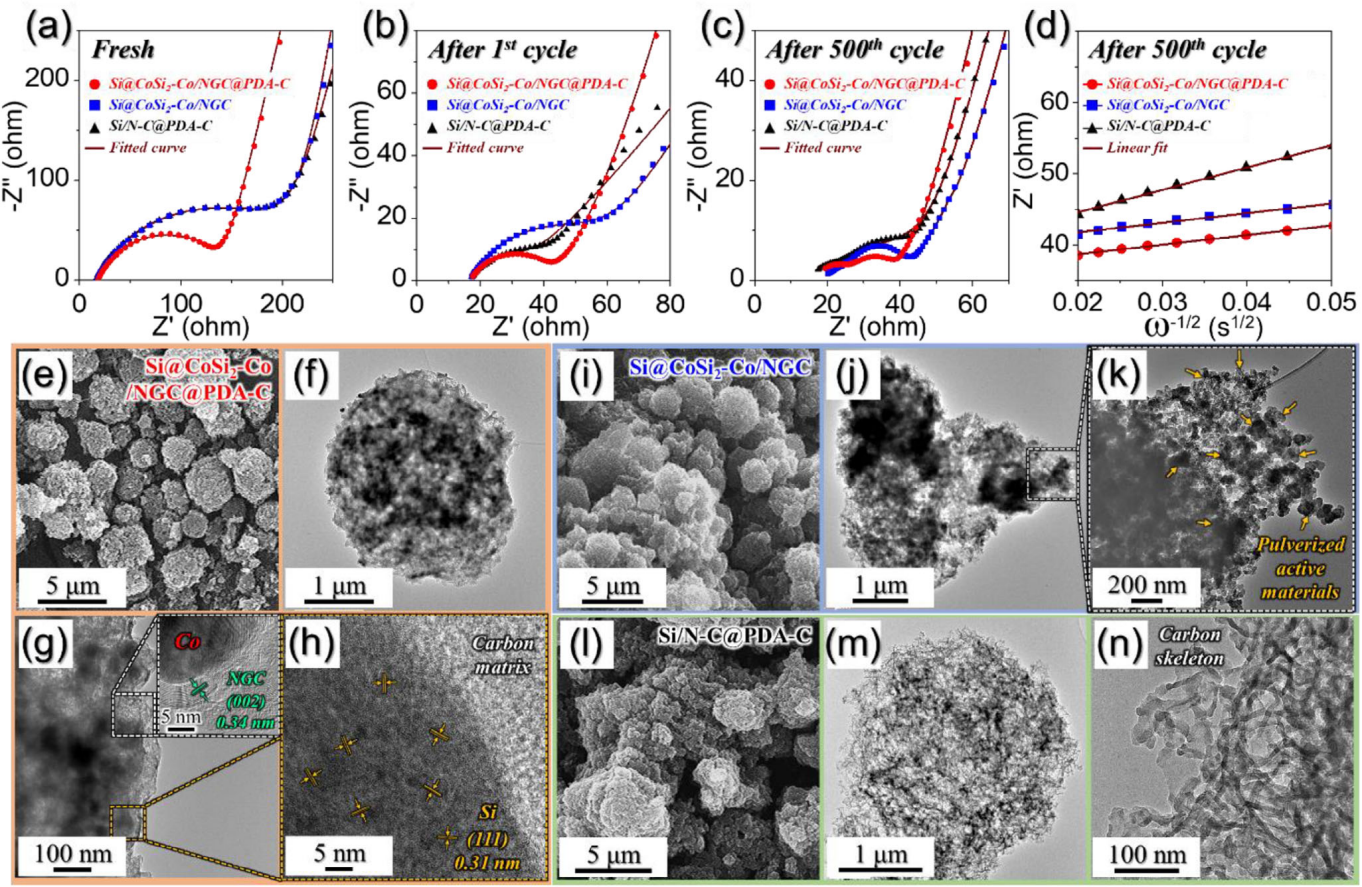
Nyquist impedance plots of the Si@CoSi_2_‐Co/NGC@PDA‐C, Si@CoSi_2_‐Co/NGC, Si/N‐C@PDA‐C anodes after various cycle number at 3.0 A g^−1^; a) before cycling, b) after 1st cycle, c) after 500th cycle, d) relationships between the real part of the impedance (*Z*_re_) and *ω* ^−1/2^ of the samples after 500th cycle; and e, i, l) FE‐SEM and f–h,j,k,m,n) TEM images of cycled e–h) Si@CoSi_2_‐Co/NGC@PDA‐C, i–k) Si@CoSi_2_‐Co/NGC, and l–n) Si/N‐C@PDA‐C anodes after 500th cycle.

To validate the exceptional Li‐ion transport properties of Si@CoSi_2_‐Co/NGC@PDA‐C microspheres, EIS measurements were systematically conducted and compared among various anodes, as illustrated in **Figure** **6**. The Nyquist plots were obtained for both fresh and cycled cells at the fully charged state. Additionally, the obtained Nyquist plots were subjected to the fitting procedure employing the deconvolution Randle‐type equivalent circuit illustrated in Figure S15 (Supporting Information), and the resultant fitted parameters are summarized in Table S4 (Supporting Information). In the plots, the experimental data are presented as scatter points, while the corresponding fitted curves are overlaid as wine‐colored solid lines to clearly differentiate the modeled response. For the Nyquist plots of the fresh cells, as depicted in Figure 6a, a striking uniformity in the *R*_s_ values (15–18 Ω) was observed across all the prepared anodes. This consistency suggests a relatively similar electrode–electrolyte interface environment for the different samples. Moreover, the *R*_ct_ values for Si@CoSi_2_‐Co/NGC@PDA‐C, Si@CoSi_2_‐Co/NGC, and Si/N‐C@PDA‐C microspheres were 137, 240, and 243 Ω, respectively. The particularly lowest *R*_ct_ value of Si@CoSi_2_‐Co/NGC@PDA‐C microspheres, compared to the other samples, can be attributed mainly to the conductive CoSi_2_ nanoplates and highly conductive carbonaceous dual shells comprising metallic‐Co embedded porous NGC and PDA‐C. Notably, the *R*_ct_ values of Si@CoSi_2_‐Co/NGC and Si/N‐C@PDA‐C microspheres were found to be similar, suggesting a similar electrochemical environment inside the cells. Following the initial cycle, a noteworthy decline in *R*_ct_ values for all samples was observed (Figure 6b), mainly due to the activation of Si particles, which led to improved charge transfer kinetics and better diffusion processes. Furthermore, the Nyquist plots after the 500th cycle (Figure 6c) were also obtained and analyzed. As observed, all anodes display two semicircles corresponding to the resistance of a SEI layer (*R*_f_) (high‐frequency region) and the *R*_ct_ (mid‐frequency region). Moreover, the *R*_ct_ values of Si@CoSi_2_‐Co/NGC@PDA‐C, Si@CoSi_2_‐Co/NGC, and Si/N‐C@PDA‐C microspheres were 24, 38, and 44 Ω, respectively. It is worth noting that the lowest *R*_ct_ value for Si@CoSi_2_‐Co/NGC@PDA‐C microspheres compared to other samples is attributed to the synergistic effects between conductive CoSi_2_ nanoplates and dual carbon shells comprising metallic‐Co embedded porous NGC and PDA‐C. These factors significantly contribute to mitigating the stress induced by the volume expansion of the electroactive species, allowing the structural integrity to persist even after 500 cycles. For further in‐depth analysis of the Nyquist plots, the real part of the impedance (Z’) was plotted against ω^−1/2^ (ω  =  2π*f* is the angular frequency) for the low‐frequency region after the 1st and 500th cycle for all samples, as shown in Figure S16 (Supporting Information) and Figure 6d. The gentle slope observed in the low frequencies for Si@CoSi_2_‐Co/NGC@PDA‐C microspheres signifies higher Li‐ion diffusivity than that of Si@CoSi_2_‐Co/NGC and Si/N‐C@PDA‐C microspheres. This enhancement underscores the improved Li‐ion diffusion properties of Si@CoSi_2_‐Co/NGC@PDA‐C microspheres due to their unique structural and compositional characteristics. Notably, the similar slope profile of Si@CoSi_2_‐Co/NGC anode after 500 cycles shown in Figure 6d is possibly due to the partial pulverization of the sample, resulting in the formation of a thicker SEI layer during prolonged cycling. Li‐ion permeable SEI and the sample's pulverized state substantially improved Li‐ion diffusion processes inside the material. Similarly, the destruction of the spherical morphology for Si/N‐C@PDA‐C microspheres allows for an increase in diffusion characteristics during repeated cycling. To verify this and confirm structural integrity, FE‐SEM and TEM analysis were conducted on all cycled electrodes after 500 continuous cycles. FE‐SEM micrographs of the cycled Si@CoSi_2_‐Co/NGC@PDA‐C electrode (Figure 6e) revealed the preservation of the original spherical morphology without noticeable rupturing or fragmentation. Consistently, TEM observations (Figure 6f–h) confirmed the robust encapsulation of metallic‐Co nanoparticles and Si particles within the intact dual carbon shell, including the NGC layer. HR‐TEM image further indicated partial amorphization and polycrystallization of Si particles due to repetitive cycling; nonetheless, their effective encapsulation within the carbon matrix reinforced exceptional structural stability (Figure 6h). In contrast, FE‐SEM (Figure 6i) and corresponding TEM images (Figure 6j,k) of the cycled Si@CoSi_2_‐Co/NGC electrode revealed evident microsphere collapse and severe pulverization of internal active materials, indicating significant structural degradation. Similarly, the cycled Si/N‐C@PDA‐C electrode exhibited complete morphological rupture and pronounced particle aggregation in FE‐SEM (Figure 6l), which was corroborated by TEM analyses (Figure 6m,n), clearly revealing complete pulverization and detachment of Si particles, leaving only fragmented amorphous carbon skeletons. This severe particle pulverization resulted from uncontrolled volume expansion and subsequent excessive SEI formation and side reactions. These comprehensive structural analyses strongly affirm that the synergistic integration of robust CoSi_2_ nanoplates and dual‐carbon shell architecture in Si@CoSi_2_‐Co/NGC@PDA‐C microspheres not only enhances electrical interconnectivity but also improves mechanical resilience against volume expansion, thereby substantially boosting the overall electrochemical performance.

### 2.4 Full‐Cell Electrochemical Performance Test

**Figure 7:**
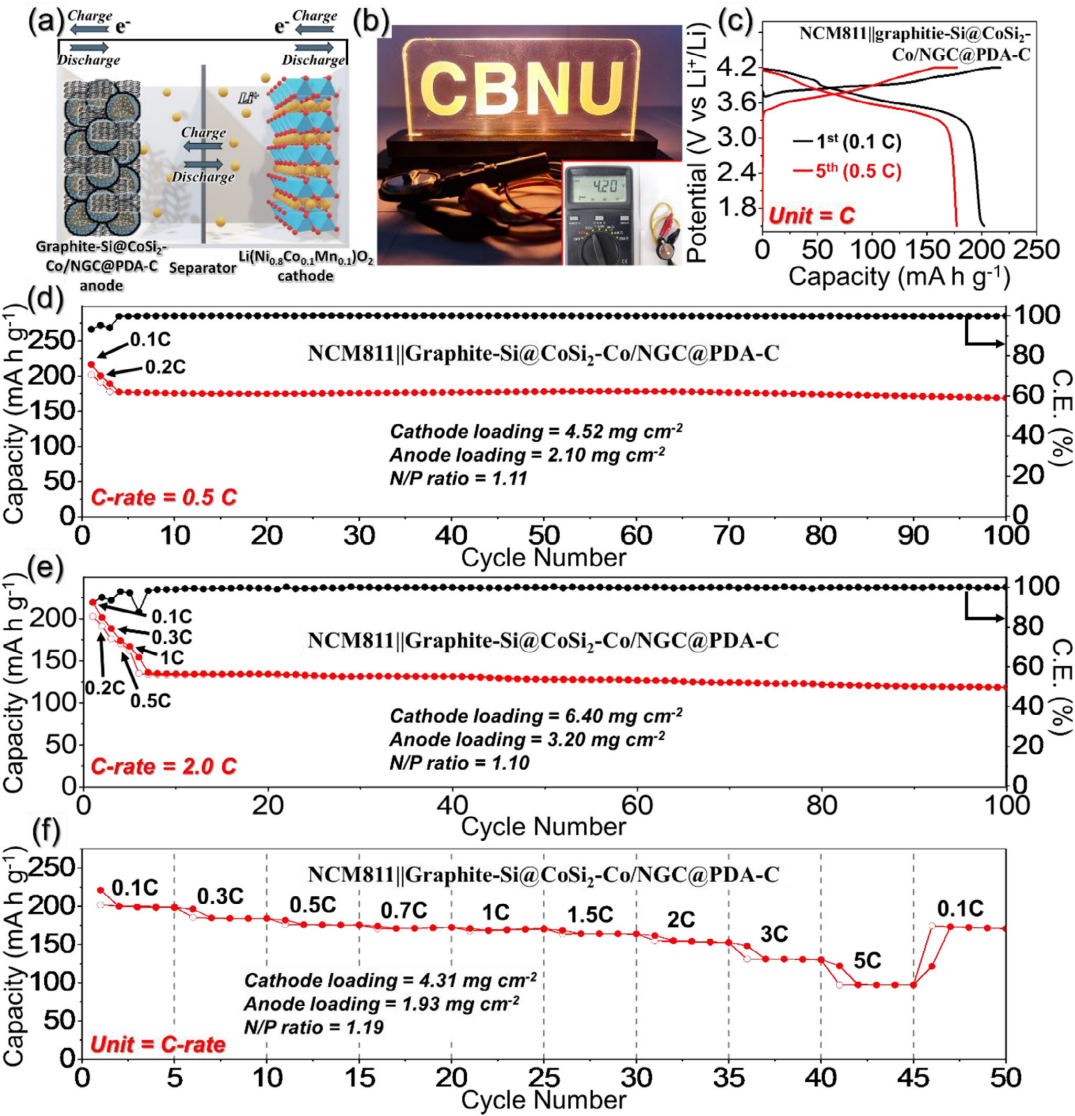
a) Schematic diagram of full‐cell consisting of Li(Ni_0.8_Co_0.1_Mn_0.1_)O_2_ (NCM811) cathode and graphite‐blended Si@CoSi_2_‐Co/NGC@PDA‐C anode, b) digital image of a light‐emitting diode (5 V, 10 mW) powered by one cell utilized after fully charging at 0.1C (1.0C = 180 mA g^−1^), c) initial Galvanostatic charge/discharge voltage profiles at current densities of 0.1C and 0.5C, d) cycling performance at 0.5C, e) cycling performance at 2.0C and f) rate performance.

The practical applicability of the Si@CoSi_2_‐Co/NGC@PDA‐C anode was evaluated through full‐cell electrochemical performance tests, pairing it with an Li(Ni_0.8_Co_0.1_Mn_0.1_)O_2_ (NCM811) cathode. Before proceeding with the full‐cell test, the cycling performance of the Si@CoSi_2_‐Co/NGC@PDA‐C anode was evaluated under constant‐current‐constant‐voltage (CC‐CV) conditions at a current density of 1.0 A g^−1^ (Figure S17a, Supporting Information). The anode delivered a high reversible capacity of 1002 mAh g^−1^ with a capacity retention of 80% after 150 cycles. To further address the commercial viability of the Si@CoSi_2_‐Co/NGC@PDA‐C, additional cycling tests were conducted under more practically relevant conditions. Specifically, the mass loading of the active material was increased to 1.3 and 1.5 mg cm^−2^, using an optimized electrode composition of active material: Super‐P:binder = 7:1.5:1.5. As shown in Figure S18 (Supporting Information), the anodes with the active material loading mass of 1.3 and 1.5 mg cm^−2^ achieved excellent discharge capacities of 1129 and 1097 mAh g^−1^, respectively, after 50 cycles at a current density of 1.0 A g^−1^. These values are highly comparable to the performance at the lower loading of 0.9 mg cm^−2^ (1125 mAh g^−1^) and showed consistent capacity retention of ≈85%, highlighting the exceptional scalability of the anode design. Furthermore, to evaluate the robustness of the Si@CoSi_2_‐Co/NGC@PDA‐C anode under harsh cycling conditions, additional electrochemical tests were conducted at elevated temperatures and high current densities (Figure S19, Supporting Information). Under a high temperature condition of 55 °C, the anode maintained a discharge capacity of 1393 mAh g^−1^ with 92% retention after 100 cycles at 1.0 A g^−1^ (Figure S19a, Supporting Information). Likewise, under high rate cycling at 3.0 and 5.0 A g^−1^, the anode exhibited excellent stability, delivering 818 and 547 mAh g^−1^ after 50 cycles with respective retentions of 83% and 75% (Figure S19b, Supporting Information). These results underscore the structural and electrochemical robustness of the dual‐shell microsphere design under thermally and kinetically demanding conditions. Additionally, the rate performance of the Si@CoSi_2_‐Co/NGC@PDA‐C anode under more practical CC‐CV conditions is evaluated. As shown in Figure S17b (Supporting Information), the electrode delivered outstanding discharge capacities of 1440, 1370, 1293, 1204, 1056, 710, 561, and 428 mAh g^−1^ at current densities of 0.1, 0.2, 0.5, 1.0, 2.0, 5.0, 7.0, and 10 A g^−1^, respectively. This excellent rate capability, especially the retention of 428 mAh g^−1^ at a high current density of 10 A g^−1^, clearly indicates the superior power characteristics of the material and its potential applicability for fast‐charging lithium‐ion batteries. Additionally, the physical and electrochemical characteristics of the commercial graphite used in the blended anodes were analyzed using FE‐SEM, XRD, cycling, and rate performance tests, as shown in Figure S20 (Supporting Information). To proceed with the full‐cell assembly, Si@CoSi_2_‐Co/NGC@PDA‐C microspheres were blended with commercial graphite in a 10:90 ratio. The blended anode tests were performed to reflect realistic and commercially viable electrode configurations. In current commercial LIB systems—particularly those used in 2nd generation electric vehicles (EVs)—silicon‐based anode materials are typically incorporated into graphite at weight ratios of ≈8:92. For next‐generation (3rd generation) EV technologies, industrial targets are moving toward higher Si contents, typically around 10–11 wt.%, to further enhance energy density while maintaining long‐term cycling stability. In alignment with these trends, the Si@CoSi_2_‐Co/NGC@PDA‐C: graphite blend ratio of 10:90 used in this study was selected to simulate near‐future commercial implementation conditions and to provide a more relevant benchmark for practical full‐cell performance. As shown in Figure S17c (Supporting Information), the graphite‐blended Si@CoSi_2_‐Co/NGC@PDA‐C anode exhibited an initial discharge capacity of 379 mAh g^−1^ at 0.5C (1.0C = 480 mA g^−1^) and retained a reversible discharge capacity of 334 mAh g^−1^ after 150 cycles. Furthermore, the blended anodes consistently outperformed commercial graphite in discharge capacity across various current densities (Figure S17d, Supporting Information). To assess the electrode swelling of graphite‐blended Si@CoSi_2_‐Co/NGC@PDA‐C anodes, electrode thickness was measured before and after initial lithiation and delithiation, with thickness variations calculated relative to the fresh electrode (Figure S21, Supporting Information). The fresh electrode exhibited an initial thickness of 40.4 µm (Figure S21a, Supporting Information). Upon lithiation, the electrode thickness increased to 45.6 µm, representing a thickness increase of 12.9% (Figure S21b, Supporting Information). Following delithiation, the electrode thickness recovered to 41.2 µm, just 2.0% thicker than the fresh electrode (Figure S21c, Supporting Information). These results underscore the structural benefits of the graphite‐blended Si@CoSi_2_‐Co/NGC@PDA‐C anodes, with their thickness variation falling within the desirable range of 10–13% typically observed for commercial graphite electrodes.^[^ ^73^, ^74^ ^]^ The thickness variations are visualized in Figure S21d (Supporting Information), further confirming the structural integrity of the blended anodes during cycling. Preliminary physical and electrochemical evaluations of the commercial NCM811 cathode were also conducted prior to integration into the full‐cell (Figure S22, Supporting Information). FE‐SEM analysis confirmed a non‐aggregated spherical morphology (Figure S22a,b, Supporting Information), while XRD results verified its rhombohedral crystal structure with an *R* $\bar{3}$ *m* space group (Figure S22c, Supporting Information). The half‐cell cycling performance demonstrated a discharge capacity of 164 mAh g^−1^ after 150 cycles at 0.5C (1.0C = 180 mA g^−1^) within a voltage range of 2.7–4.3 V (Figure S22d, Supporting Information). Additionally, the NCM811 cathode achieved discharge capacities of 195, 171, 153, 137, 112, 88, 69, and 54 mAh g^−1^ at current densities of 0.1C, 0.3C, 0.5C, 0.7C, 1.0C, 1.5C, 2.0C, and 3.0C, respectively (Figure S22e, Supporting Information). Based on these results, full‐cells were assembled using the graphite‐blended Si@CoSi_2_‐Co/NGC@PDA‐C anode and commercial NCM811 cathode to investigate the practical application potential of the synthesized materials (**Figure** **7**). The full‐cells operated within a voltage range of 1.5–4.2 V, with active material loading on the anode and cathode set at ≈2.1 and 4.52 mg cm^−2^, respectively, ensuring an N/P ratio of ≈1.1 for practical applications. A schematic diagram of the NCM811||graphite‐Si@CoSi_2_‐Co/NGC@PDA‐C full‐cell is provided in Figure 7a. Upon charging to 4.2 V at 0.1C (1.0C = 180 mA g^−1^), the full‐cell successfully powered a 5 V, 10 mW light‐emitting diode, demonstrating its potential for large‐scale energy storage (Figure 7b). The full‐cell exhibited initial charge/discharge capacities of 217/202 mAh g^−1^ at 0.1C and 178/177 mAh g^−1^ at 0.5C, with an average discharge voltage of ≈3.6 V (Figure 7c). After 100 cycles at 0.5C, the cell retained a discharge capacity of 169 mAh g^−1^, corresponding to a capacity retention of 96% with a high Coulombic efficiency of 99.95%, highlighting the durability and viability of the Si@CoSi_2_‐Co/NGC@PDA‐C anode (Figure 7d). Based on these results, the gravimetric energy density of the full‐cell was calculated to be ≈406 Wh kg^−1^, using a measured average discharge voltage of 3.5 V obtained from the discharge profiles shown in Figure 7d. Detailed parameters and calculation results are summarized in Supporting Information and Table S5 (Supporting Information), confirming the competitive energy output of the full‐cell under practical electrode loading conditions. To further assess its practical applicability, full‐cell tests were performed under a high‐loading configuration with cathode and anode mass loadings of 20.9 and 6.55 mg cm^−2^, respectively. Under these conditions, the full‐cell delivered a discharge capacity of 176 mAh g^−1^ at 0.1C after 45 cycles (Figure S23, Supporting Information), resulting in a higher calculated energy density of 469 Wh kg^−1^ (Table S5, Supporting Information). To assess fast‐charging capability, a full‐cell with electrode loadings of 6.4 and 3.2 mg cm^−2^ (cathode/anode) was cycled at 2.0C (Figure 7e). Impressively, the cell retained a reversible discharge capacity of 119 mAh g^−1^ after 100 cycles, corresponding to a capacity retention of 88%, thereby underscoring the fast‐charging suitability and structural integrity of the Si@CoSi_2_‐Co/NGC@PDA‐C anode even under aggressive cycling conditions. Additionally, the full‐cell demonstrated excellent rate capability, achieving discharge capacities of 199, 184, 176, 173, 171, 164, 152, 130, and 98 mAh g^−1^ at current densities of 0.1C, 0.3C, 0.5C, 0.7C, 1.0C, 1.5C, 2.0C, 3.0C, and 5.0C, respectively (Figure 7f). These results underscore the strong potential of the Si@CoSi_2_‐Co/NGC@PDA‐C anode for practical applications when paired with an NCM811 cathode. To further validate the intrinsic electrochemical performance of the Si@CoSi_2_‐Co/NGC@PDA‐C anode, a full‐cell configuration without graphite‐blend was additionally evaluated under more stringent conditions (Figure S24, Supporting Information). In this setup, the Si@CoSi_2_‐Co/NGC@PDA‐C anode was used as the sole active material with a mass loading of ≈1.0 mg cm^−2^, while the NCM811 cathode was loaded at ≈6.1 mg cm^−2^, maintaining an N/P ratio of 1.10. The resulting full‐cell exhibited excellent cycling performance, delivering a reversible discharge capacity of 179 mAh g^−1^ after 80 cycles at 0.5C with a high retention of 99% (Figure S24a, Supporting Information). Furthermore, the rate capability was well preserved, with discharge capacities of 185, 164, 151, 141, 132, 121, 109, 82, and 56 mAh g^−1^ at current densities ranging from 0.1C to 5.0C, respectively (Figure S24b, Supporting Information). These findings clearly demonstrate that even without graphite blending, the Si@CoSi_2_‐Co/NGC@PDA‐C anode retains its structural stability and high rate capability, highlighting its robustness and suitability for demanding full‐cell applications.

## 3 Conclusion

In summary, we successfully synthesized 3D porous microspheres incorporating Si nanocrystals armed with CoSi_2_ nanoplates as multi‐cores, encapsulated within dual protective shells composed of metallic‐Co nanocrystals embedded in highly conductive NGC and PDA‐C. This novel anode design addresses the key challenges of Si‐based lithium‐ion batteries by combining a straightforward and cost‐effective fabrication process—featuring aerosol‐assisted spray pyrolysis, optimized PDA coating, and carbonization—with advanced material engineering. In this synergistic structure, the Si functions as the active material, while the inactive buffer CoSi_2_ nanoplate enhances electrical interconnectivity and structural robustness within the nanostructure. Likewise, the dual protective shells of metallic‐Co embedded porous NGC and PDA‐C serve as the primary and secondary transport pathways, respectively. This configuration facilitates smooth electron transfer and effectively guards the Si particles against pulverization. Correspondingly, the Si@CoSi_2_‐Co/NGC@PDA‐C anode, developed through this synergistic approach, demonstrates exceptional electrochemical performance, including high‐rate capability (up to 10 A g^−1^) and remarkable capacity retention over extended cycling (88% after 600 cycles at 1.0 A g^−1^ and 70% after 1000 cycles at 3.0 A g^−1^). Therefore, it is anticipated that the rational design strategy elucidated in this study will significantly contribute to the practical development of Si‐based lithium battery anodes.

## 4 Experimental Section

### Sample Preparation—Synthesis of 3D Si@CoO/NGC Microspheres

The 3D Si@CoO/NGC microspheres as precursor powders were prepared via the aerosol‐assisted spray pyrolysis technique. Briefly, 0.15 m of Co(NO_3_)_2_·6H_2_O (SAMCHUN, Extra Pure, 97%, M_w_ = 291.02) was first dispersed in 200 mL of distilled water with continuous stirring. Subsequently, 2.0 g of PVP (DAEJUNG, M_w_ = 40000) was added to the solution as dispersant and carbon source. Finally, 4.0 g of as‐received Si nanopowders (Lioyang Tongrun Info Technology Co., Ltd., >99%, *ϕ* = 50–100 nm) were added to the above solution and stirred overnight to ensure homogeneous dispersion of Si nanoparticles. The resulting aqueous spray solution was atomized using an ultrasonic atomizer connected to a preheated quartz tube maintained at a temperature of 700 °C. Nitrogen (N_2_) gas was used as the carrier gas, flowing through the system at a rate of 10 L min^−1^. The atomizer generated homogeneously dispersed aqueous droplets, which were then fed through the preheated quartz tube. Finally, the as‐sprayed powder was carefully collected and stored for subsequent utilization.

### Sample Preparation—Synthesis of Si@CoSi_2_‐Co/NGC@PDA‐C and Si@CoSi_2_‐Co/NGC Microspheres

The as‐sprayed Si@CoO/NGC microspheres served as the precursor powder for the ex situ PDA‐C coating. The coating process was followed by carbonization, resulting in the eventual fabrication of Si@CoSi_2_‐Co/NGC@PDA‐C microspheres as a high‐performance lithium battery anode. Briefly, 100 mg of the as‐sprayed powder was dispersed in 100 mL of tris buffer solution (0.01 m, pH: 8.7) to prepare the PDA‐C‐coated microspheres. Subsequently, dopamine hydrochloride (Alfa Aesar, 99%, M_w_ = 189.64) weighing 50 mg was introduced into the above solution. The mixture was stirred for 16 h under ambient conditions. The PDA‐coated Si@CoO/NGC microspheres underwent multiple washing cycles with distilled water. These coated microspheres were then collected via centrifugation and subsequently dried in a hot air oven at 60 °C. The final step involved the microspheres in a carbonization process, which was carried out at a temperature of 800 °C for 5 h in a N_2_ atmosphere. This resulted in the successful synthesis of Si@CoSi_2_‐Co/NGC@PDA‐C microspheres. Moreover, for comparison purposes, the sample without a PDA‐C shell, namely “Si@CoSi_2_‐Co/NGC” microspheres, was also utilized using identical as‐sprayed powders followed by carbonization at 800 °C for 5 h in N_2_ atmosphere.

### Sample Preparation—Synthesis of Si/N‐C@PDA‐C Microspheres

To facilitate a meaningful comparison, a standard sample was also prepared without CoSi_2_ nanoplates and metallic‐Co embedded NGC shell via a spray drying process. It should be noted that the spray pyrolysis technique encountered trouble for this specific sample, as it failed to form aqueous droplets. The spray drying solution was prepared without the Co precursor; however, all other concentrations remained consistent with the previous procedure. The spray dryer was operated under specific conditions, maintaining an inlet/outlet temperature of 190/100 °C, a flow rate of 0.8 mL min^−1^, and automizing air pressure at 0.2 MPa. Following the spray drying process, the resultant sample underwent carbonization at 400 °C for 3 h in N_2_ atmosphere. Afterward, the sample was subjected to PDA‐C coating via the process discussed above, followed by an additional heat‐treatment at 400 °C for 3 h in N_2_ atmosphere. The finally obtained sample was abbreviated as “Si/N‐C@PDA‐C” microspheres.

### Characterization Techniques

The crystal structure of the synthesized microspheres, namely Si@CoSi_2_‐Co/NGC@PDA‐C, Si@CoSi_2_‐Co/NGC, and Si/N‐C@PDA‐C, was determined using a Bruker X‐ray diffraction (XRD) instrument (D2, 2nd generation) equipped with Cu Kα radiation (λ = 1.5418 Å). Morphological analyses were conducted using field‐emission scanning electron microscopy (FE‐SEM; Zeiss) and field‐emission transmission electron microscopy (FE‐TEM; JEM‐2100F, JEOL) at the Korea Basic Science Institute (Daegu). Scanning transmission electron microscopy (STEM) was further performed using a Cs‐corrected transmission electron microscope (JEM‐ARM200F, NEOARM, with STEM‐Cs) at the Center for Research Facilities (CRIEF), Chungbuk National University, to obtain high‐resolution structural and compositional insights. Thermogravimetric analysis (TGA) was performed in an air atmosphere from 25 to 800 °C at a ramp rate of 10 °C min^−1^. The bonding states and chemical environments of the different elements within the prepared powders were determined using X‐ray photoelectron spectroscopy (XPS; K‐Alpha, Thermo Scientific) with an Al Kα X‐ray source at Sunchon National University Center for Research Facilities. The specific surface area and pore size distribution of the synthesized powders were determined using N_2_ adsorption–desorption isotherms, with calculations based on the Brunauer–Emmett–Teller (BET) method. Elemental analysis (EA) was employed to quantify the carbon and nitrogen content of the samples. The crystalline characteristics of the carbonaceous products in the prepared microspheres were studied using Raman spectroscopy (LabRam, HR800, Horiba Jobin‐Yvon).

### Cell Assembly and Electrochemical Measurements

The electrochemical properties of the samples were measured using 2032‐type coin cells. The working electrodes, comprising active material, conductive carbon (Super‐P), and sodium carboxymethyl cellulose (CMC) as a binder in a mass ratio of 6:2:2, were prepared using a slurry casting method on a copper foil. The electrodes were subsequently dried overnight at 60 °C in a hot air oven. The circular electrodes (*ϕ* = 14 mm) with an active material mass loading of ≈0.9 mg cm^−2^ (active material mass = 1.38 mg) were punched and transferred inside the glovebox. Li metal and microporous polypropylene film were used as the counter electrode and separator, respectively. The electrolyte used was 1.15 m LiPF_6_ in a mixture of ethylene carbonate (EC), ethyl methyl carbonate (EMC), and dimethyl carbonate (DMC) with a volume ratio of 2:4:4. Additionally, vinylene carbonate (VC, 1.0 wt.%), lithium difluorophosphate (LiPO_2_F_2_, 1.0 wt.%), and fluoroethylene carbonate (FEC, 12.5 wt.%) were used as additives. To evaluate the full‐cell performance, the cathodes were prepared by casting a slurry of Li(Ni_0.8_Co_0.1_Mn_0.1_)O_2_ (NCM811) powder, Super‐P, and poly(vinylidene fluoride) at a weight ratio of 8:1:1 in 1‐methyl‐2‐pyrrolidinone (NMP) solvent onto an aluminum current collector. The coin cell was assembled at room temperature (25 °C) in an Ar‐filled glove box. The electrochemical performances of the samples were evaluated using cyclic voltammetry (CV), charge–discharge testing, and electrochemical impedance spectroscopy (EIS). The voltage window throughout the electrochemical tests was fixed at 0.001–3.0 V. The CV measurements of the samples were performed at a scan rate of 0.1 mV s^−1^. The charge–discharge testing of the samples was conducted at various current densities from 0.1 to 10 A g^−1^ using a WBCS3000 (WonATech) cycler. The EIS data of the samples were collected in the frequency range of 100 kHz–0.01 Hz using a signal amplitude of 10 mV. The electrochemical properties of the full‐cell, paired with a Si@CoSi_2_‐Co/NGC@PDA‐C anode and an NCM811 cathode, were evaluated at various current densities ranging from 0.1C to 5.0C (1.0C = 180 mA g^−1^) over the voltage range of 1.5–4.2 V, with the N/P ratio fixed at ≈1.1.

## Conflict of Interest

The authors declare no conflict of interest.

## Author Contributions

J.S.L., H.S.A., and J.Y.K. contributed equally to this work. J.S.L. did conceptualization, methodology, validation, formal analysis, investigation, data curation, wrote the original draft, and visualization. H.S.A. and J.Y.K. did methodology, validation, formal analysis, investigation, and data curation. R.S. did data curation, wrote the original draft, and visualization. B.S.J. did formal analysis, investigation, and data curation. J.H.B. did methodology. Y.C.K. gave advise on the research. G.D.P. did resources. D.S.J. did resources. D.‐W.K. did supervision, and resources. C.C. did supervision, and resources. J.S.C. did conceptualization, supervision, review and editing, project administration, and funding acquisition.

## Supporting information



Supporting Information

## Data Availability

The data that support the findings of this study are available from the corresponding author upon reasonable request.
