# Supplementary material for: Shielding Si Armed with CoSi2 Nanoplates and Dual‐Carbon Shells: 3D Porous Microspheres as High‐Performance Anodes for Li‐Ion Batteries
Source: Adv Sci (Weinh). 2025 Jul 29;12(40):e08213. doi: 10.1002/advs.202508213 (PMC12561417; doi:10.1002/advs.202508213)
Supplement: Supplementary file 1 — Supporting Information [file ADVS-12-e08213-s001.docx]

Supporting Information

Shielding Si armed with CoSi_2_ nanoplates and dual-carbon shells: three-dimensional porous microspheres as high-performance anodes for Li-ion batteries

*Jae Seob Lee, Hyun Seon Ahn, Jung Yeon Kim, Rakesh Saroha, Beom Su Jo, Ji Hun Baek, Yun Chan Kang, Gi Dae Park, Dae Soo Jung, Dong-Won Kang*, Chungyeon Cho*, and Jung Sang Cho**

J. S. Lee, H. S. Ahn, J. Y. Kim, R. Saroha, B. S. Jo, J. H. Baek, J. S. Cho

Department of Engineering Chemistry

Chungbuk National University

Chungbuk, 28644, Republic of Korea

E-mail: jscho@cbnu.ac.kr

J. S. Lee, Y, C Kang

Department of Materials Science and Engineering

Korea University

Seoul, 02841, Republic of Korea

R. Saroha

Department of Materials Science and Engineering

Ajou University

Suwon-si, Gyeonggi-do, 16499, Republic of Korea.

G. D. Park

Department of Advanced Materials Engineering

Chungbuk National University

Chungbuk, 28644, Republic of Korea

D. S. Jung

Energy Storage Materials Center

Korea Institute of Ceramic Engineering and Technology

Jinju, Gyeongnam 52851, Republic of Korea

D.-W. Kang

School of Energy Systems Engineering

Chung-Ang University

Dongjak-Gu, Seoul, 06974, Republic of Korea

E-mail: kangdwn@cau.ac.kr

C. Cho

Department of Biomedical Materials Science

Jeonbuk Advanced Bio-convergence Academy, Wonkwang University

Iksan, Jeonbuk 54538, Republic of Korea

E-mail: cncho37@wku.ac.kr

J. S. Cho

Biomedical Research Institute, Chungbuk National University Hospital, Chungbuk 28644, Republic of Korea

Advanced Energy Research Institute, Chungbuk National University, Cheongju, Chungbuk 28644, Republic of Korea

E-mail: jscho@cbnu.ac.kr

J. S. Lee, H. S. Ahn, and J. Y. Kim contributed equally to this work.

Keywords: lithium-ion batteries, silicon anodes, metal silicide, nitrogen-doped graphitic carbon, polydopamine-derived carbon shell, full-cell


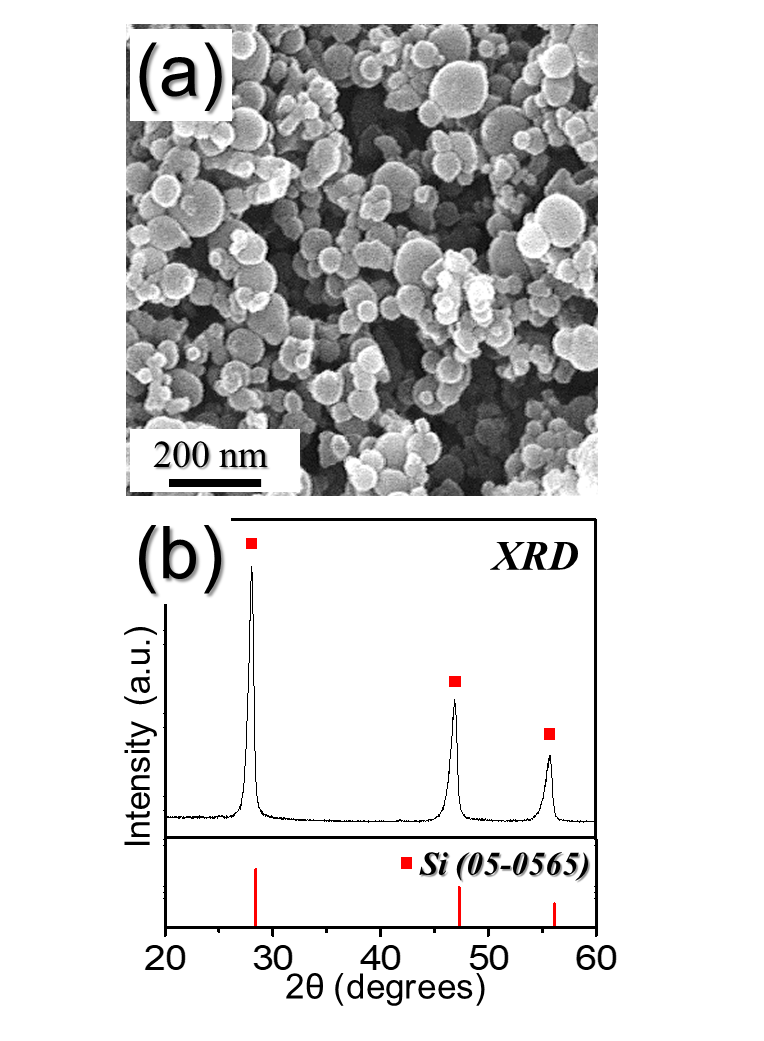


**Figure S1.** a) FE-SEM image and b) XRD pattern of commercial Si nanopowder used to prepare the spray solution.


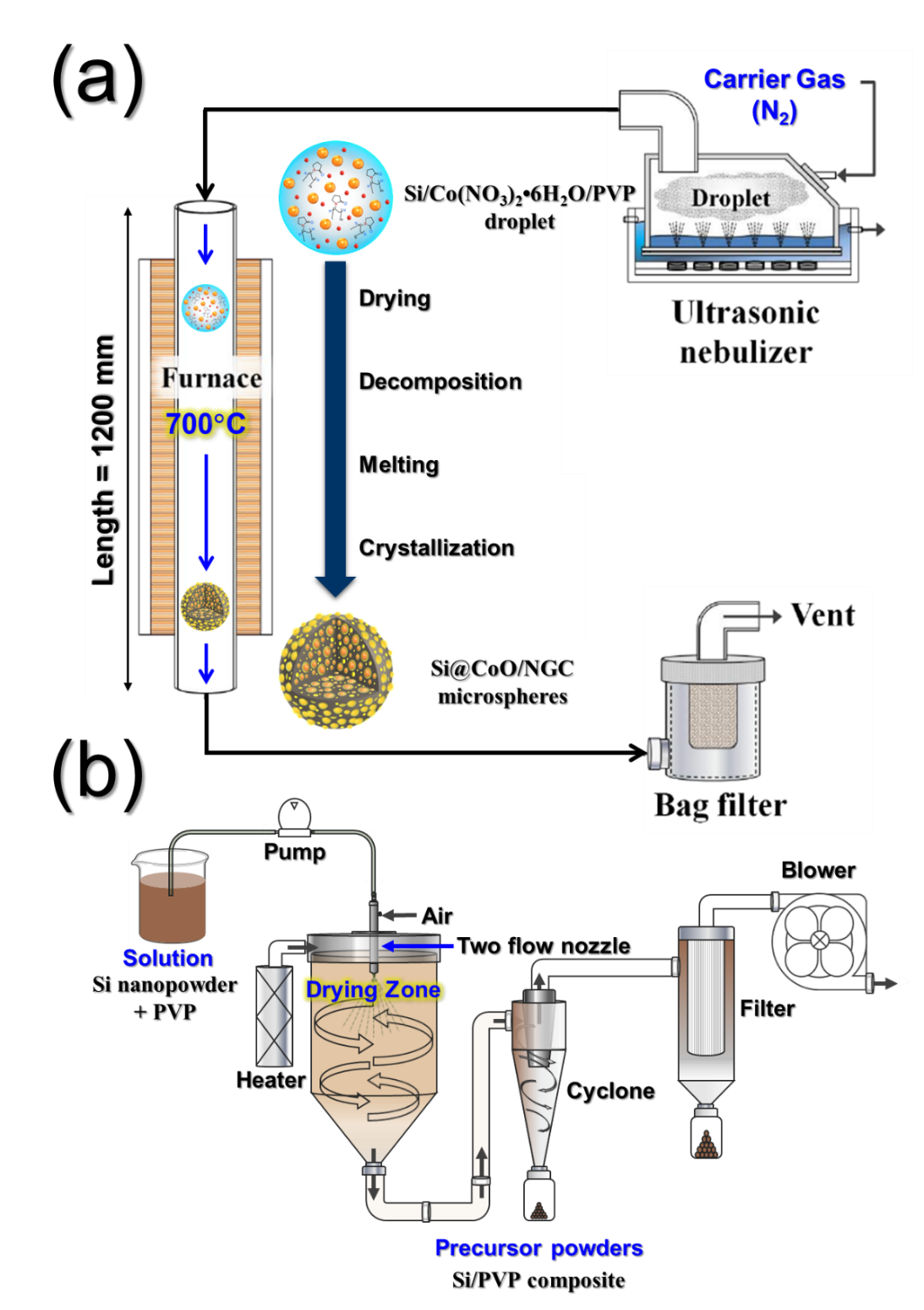


**Scheme S1.** a) Schematic diagram of the spray pyrolysis process for the preparation of Si@CoO/NGC composite microspheres, and b) schematic diagram of the spray drying process for the preparation Si/PVP composite microspheres.

**
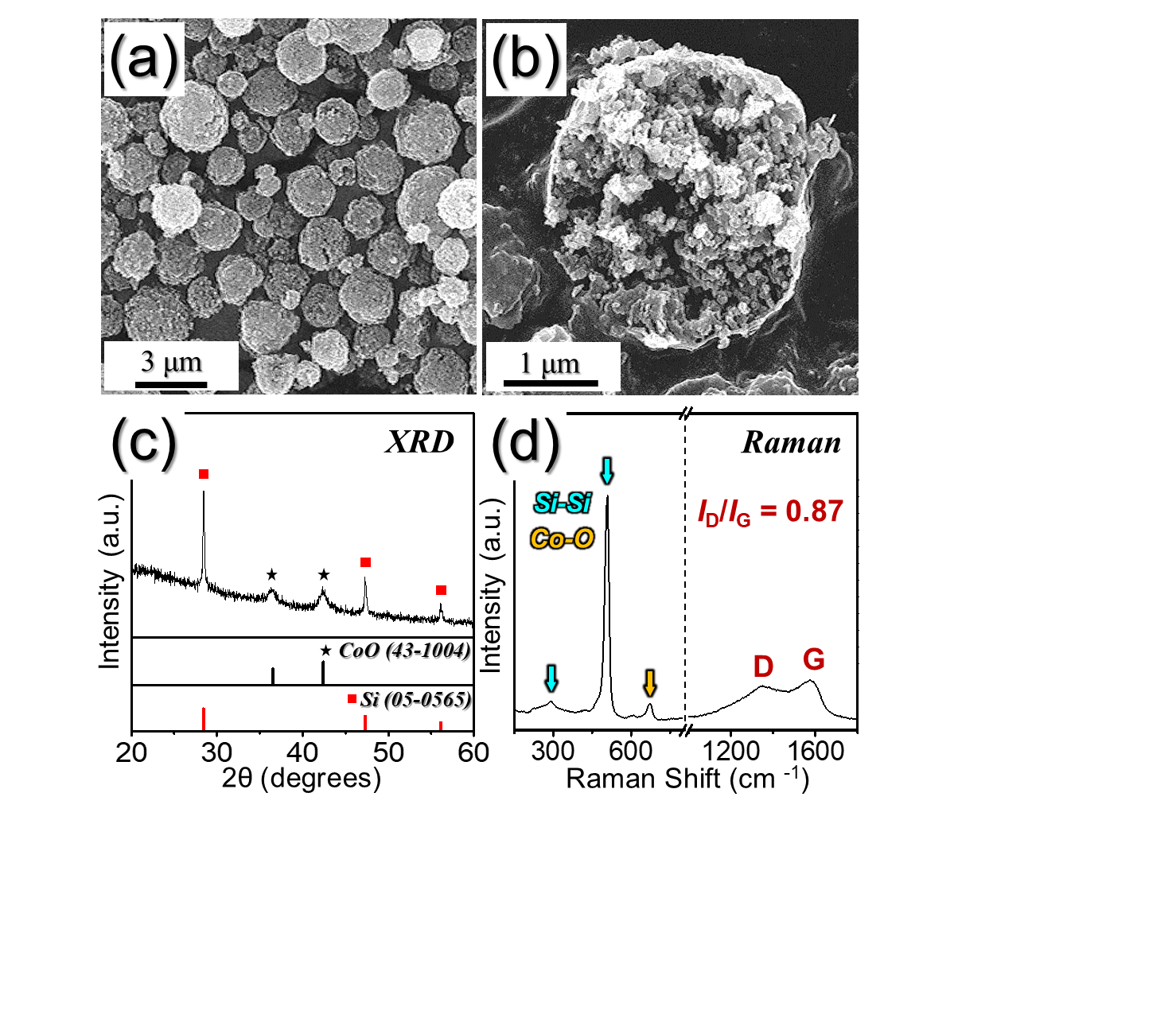
**

**Figure S2.** a) FE-SEM image, b) fractured micrograph of the microsphere, c) XRD pattern, and d) Raman spectrum of Si@CoO/NGC microspheres obtained after spray pyrolysis at 700 ℃.


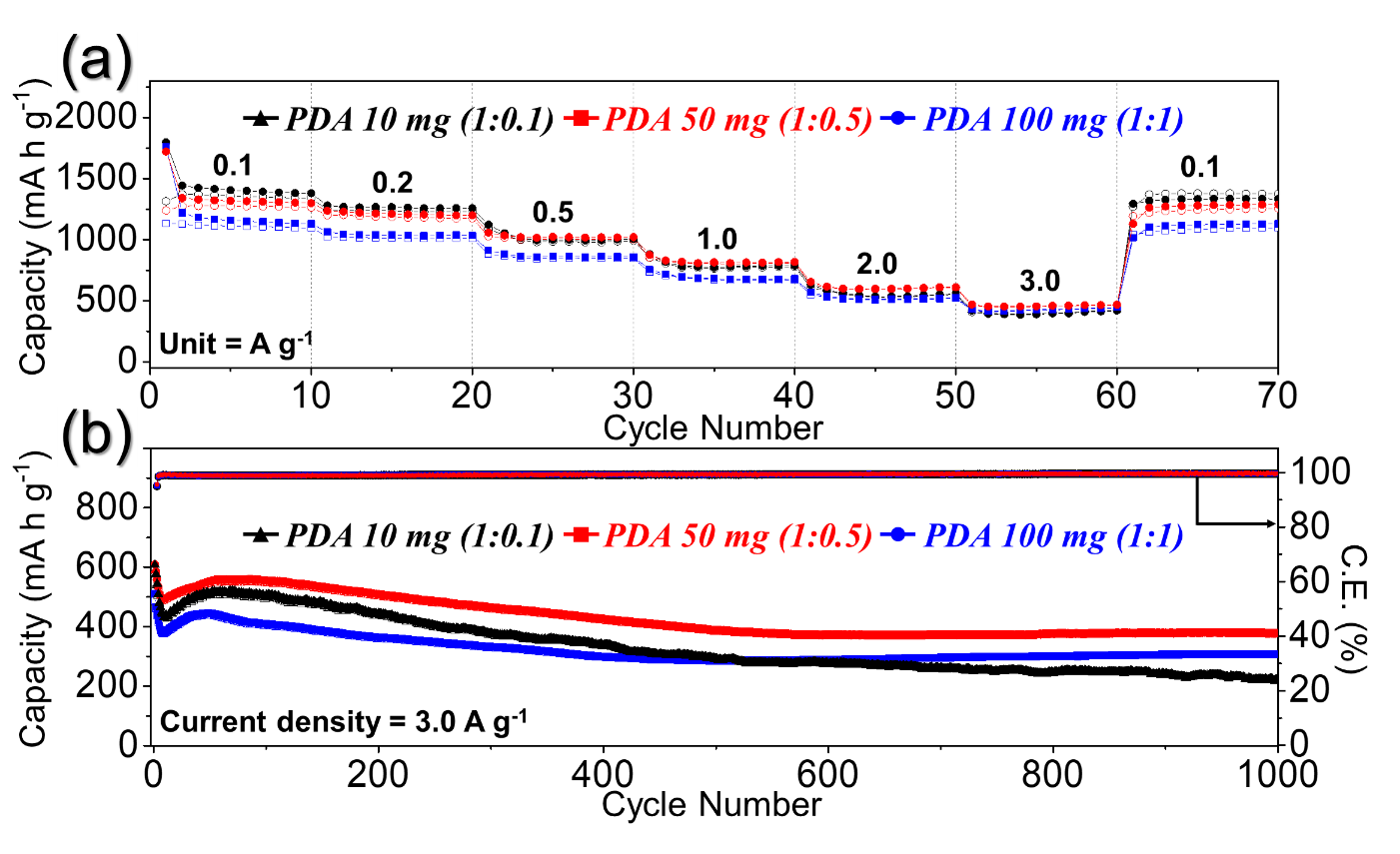


**Figure S3.** Electrochemical properties of Si@CoSi_2_-Co/NGC@PDA-C microspheres with different mass ratio of PDA obtained after carbonization at 800 ℃; a) rate capability at various current densities ranging from 0.1 to 3.0 A g^−1^ and b) cycle performances at a current density of 3.0 A g^−1^.

**Table S1.** Elemental analysis (EA) results of Si@CoSi_2_-Co/NGC@PDA-C microspheres prepared with different mass ratio of PDA.

| Sample | Carbon (wt%) | Nitrogen (wt%) |
| --- | --- | --- |
| PDA 10 mg (1:0.1) | 1.7 | 0.9 |
| PDA 50 mg (1:0.5) | 14.3 | 1.3 |
| PDA 100 mg (1:1) | 27.0 | 1.7 |


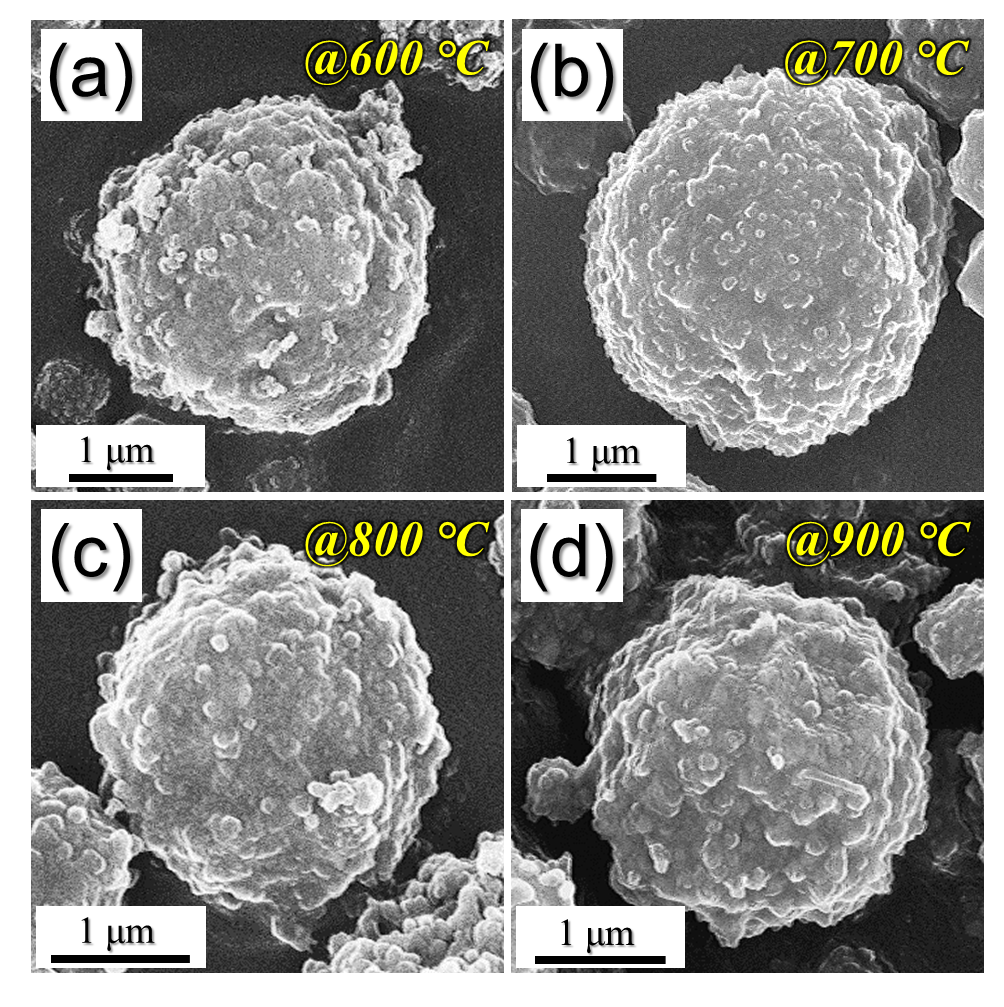


**Figure S4.** FE-SEM images of 50 mg of PDA-coated Si@CoO/NGC powder obtained after various carbonization temperature (600–900 ℃); a) 600 ℃, b) 700 ℃, c) 800 ℃, and d) 900 ℃.


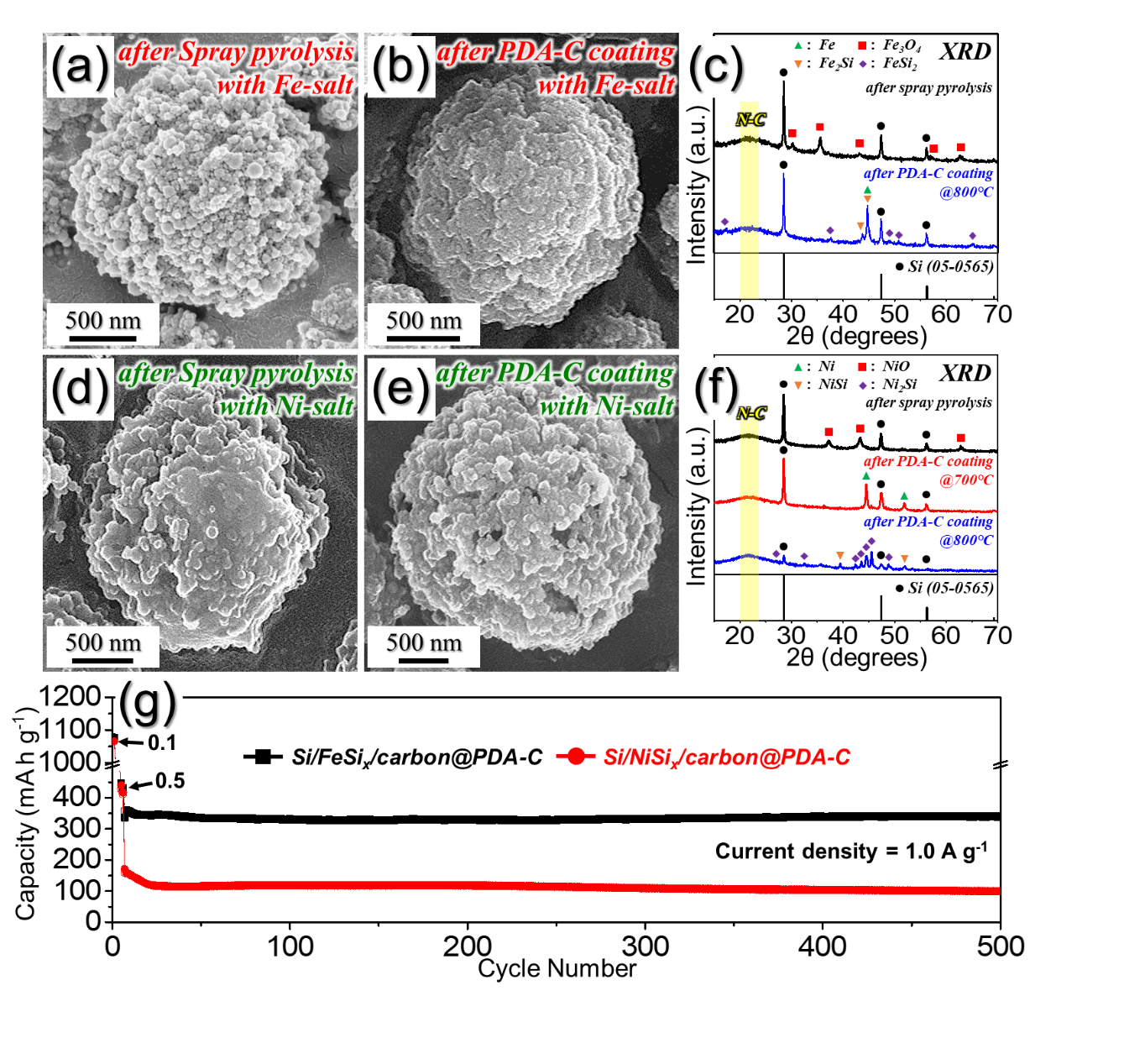


**Figure S5.** a) FE-SEM images of microspheres after spray pyrolysis with Fe-salt and b) Si/FeSi*_x_*/carbon@PDA-C obtained after PDA-C coating followed by annealing at 800 ℃, c) Corresponding XRD patterns, d) FE-SEM images of microspheres after spray pyrolysis with Ni-salt and e) Si/NiSi*_x_*/carbon@PDA-C obtained after PDA-C coating followed by annealing at 800 ℃, f) Corresponding XRD patterns; g) cycling performance of Si/FeSi*_x_*/carbon@PDA-C and Si/NiSi*_x_*/carbon@PDA-C anodes at 1.0 A g^−1^.

To further investigate the influence of different transition metals on the structural and electrochemical characteristics of the silicon-based multi-core dual-shell microspheres, microspheres with Fe- and Ni-species were synthesized by replacing the Co precursor with Fe and Fe precursors under identical synthesis conditions. These transition metals—Co, Fe, and Ni—were selected due to their ability to form stable and conductive silicide phases concurrently with conductive GC shells upon heat treatment. FE-SEM confirmed similar spherical morphologies (Figure S5b and e), while XRD patterns revealed Fe_2_Si, FeSi_2_, and metallic-Fe phases in Fe-based microspheres and NiSi and Ni_2_Si phases in Ni-based microspheres (Figure S5c and f). Electrochemical tests at 1.0 A g^−1^ revealed significantly lower capacities for Si/FeSi*_x_*/carbon@PDA-C (341 mA h g^−1^ after 500 cycles) due to high Fe_2_Si resistivity (0.93–3.9 × 10^4^ Ω cm).^[S14]^ The Si/NiSi*_x_*/carbon@PDA-C anode, despite the low resistivity of Ni_2_Si (~21 µΩ cm), exhibited an even lower capacity of 100 mA h g^−1^ due to excessive consumption of active Si during rapid and uncontrolled formation of nickel silicide phases at high temperatures.^[S15]^ In contrast, the Si@CoSi_2_-Co/NGC@PDA-C anode outperformed both Fe- and Ni-based systems, retaining a high capacity of approximately 900 mA h g^−1^ after 600 cycles (Figure 1e). This superior performance is attributed to the optimal formation of CoSi_2_, which combines relatively low resistivity (10–25 µΩ cm), structural stability, and controllable reactivity with Si, ensuring preservation of active material and enhanced electrochemical kinetics.^[47]^ These comparative results highlight the critical role of transition metal selection in designing high-performance silicon-based anodes. Among the tested metals, Co was conclusively determined to be the most effective, providing the best combination of electrical conductivity, structural integrity, and electrochemical performance.


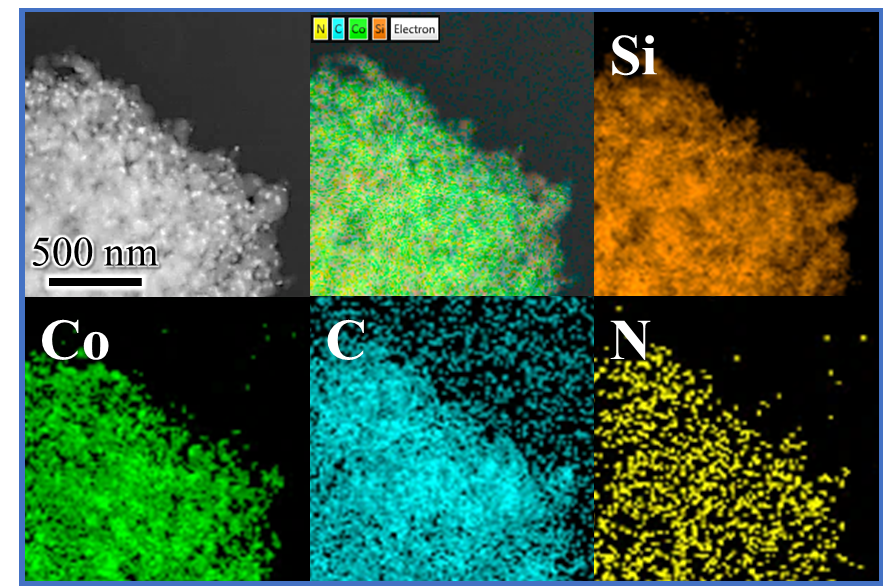


**Figure S6.** High-magnification STEM-elemental mapping images of Si@CoSi_2_-Co/NGC@PDA-C microspheres.

**Table S2.** Elemental analysis (EA) results of Si@CoSi_2_-Co/NGC@PDA-C, Si@CoSi_2_-Co/NGC, and Si/N-C@PDA-C microspheres.

| Sample | Carbon (wt%) | Nitrogen (wt%) |
| --- | --- | --- |
| Si@CoSi_2_-Co/NGC@PDA-C | 14.3 | 1.3 |
| Si@CoSi_2_-Co/NGC | 0.5 | 0.3 |
| Si/N-C@PDA-C | 9.8 | 2.0 |


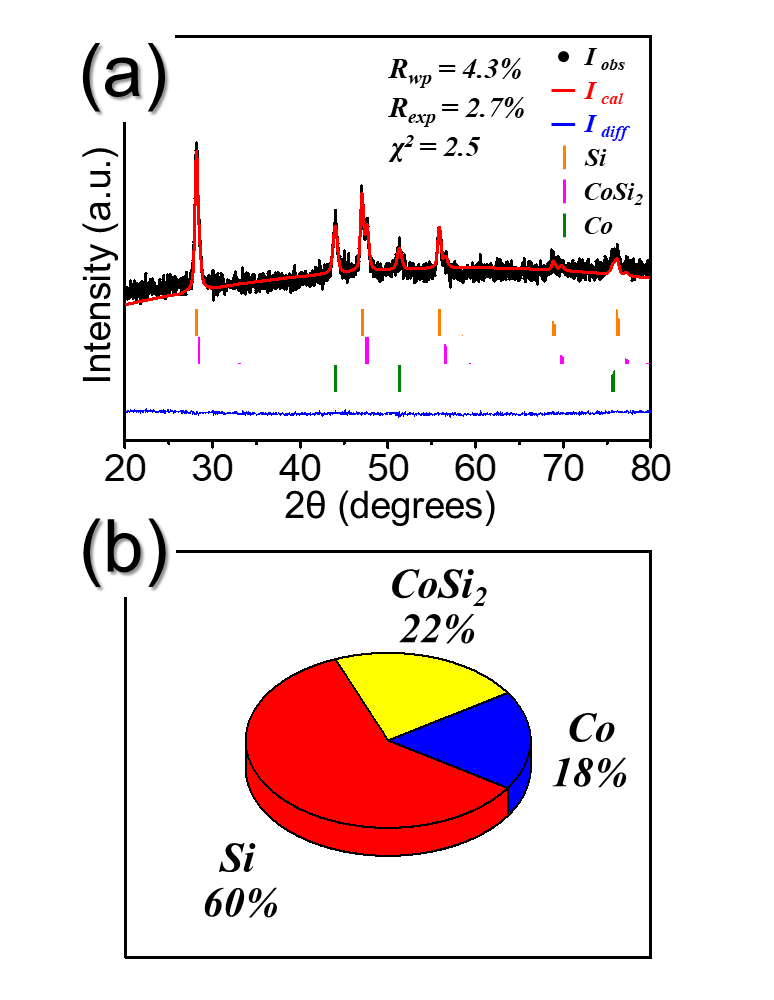


**Figure S7.** a) Rietveld refinement of Si@CoSi_2_-Co/NGC@PDA-C composite microspheres, and b) Pie-chart showing the mole fractions of Si, CoSi_2_, and metallic-Co phase.

**
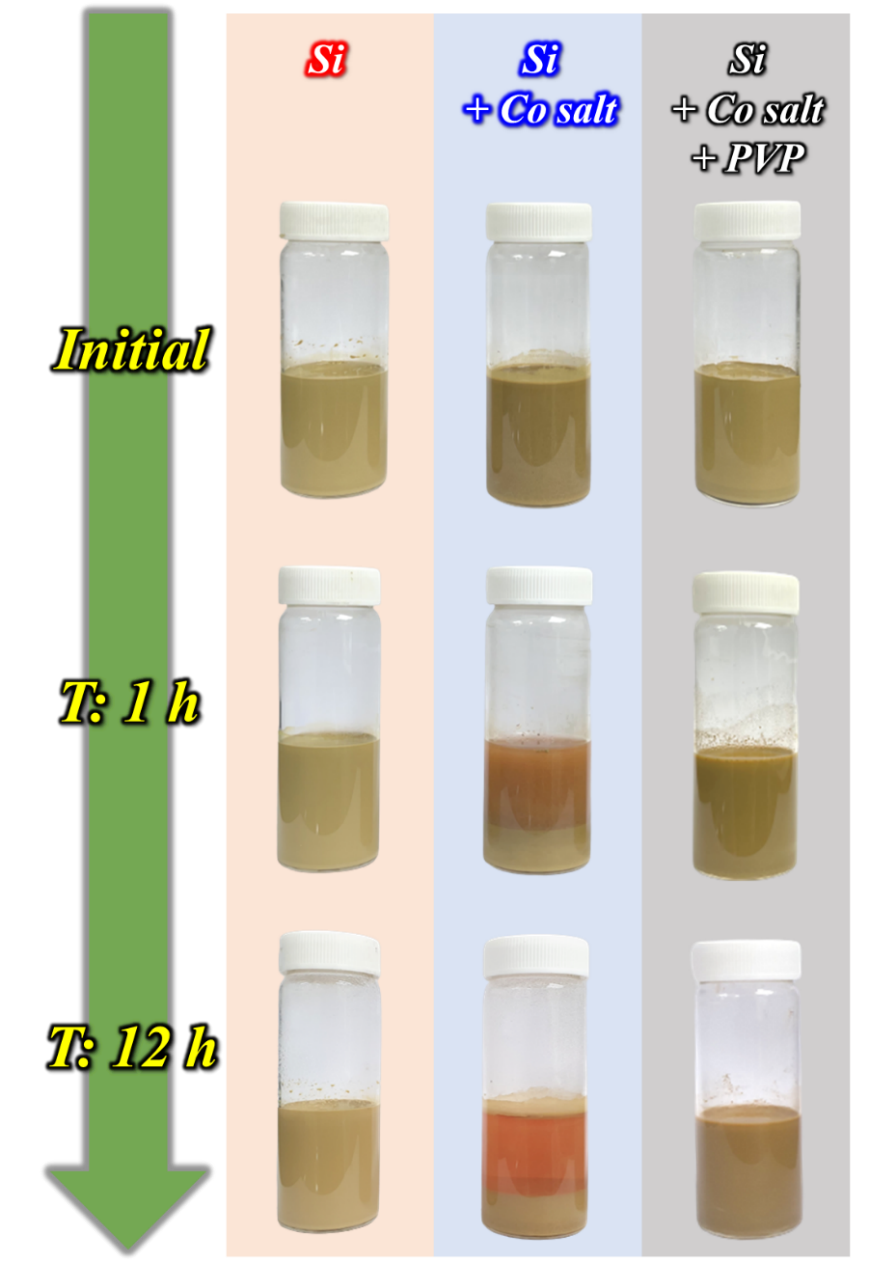
**

**Figure S8.** Digital images of the dispersion process of various precursors inside the spray solution at different time intervals.

**
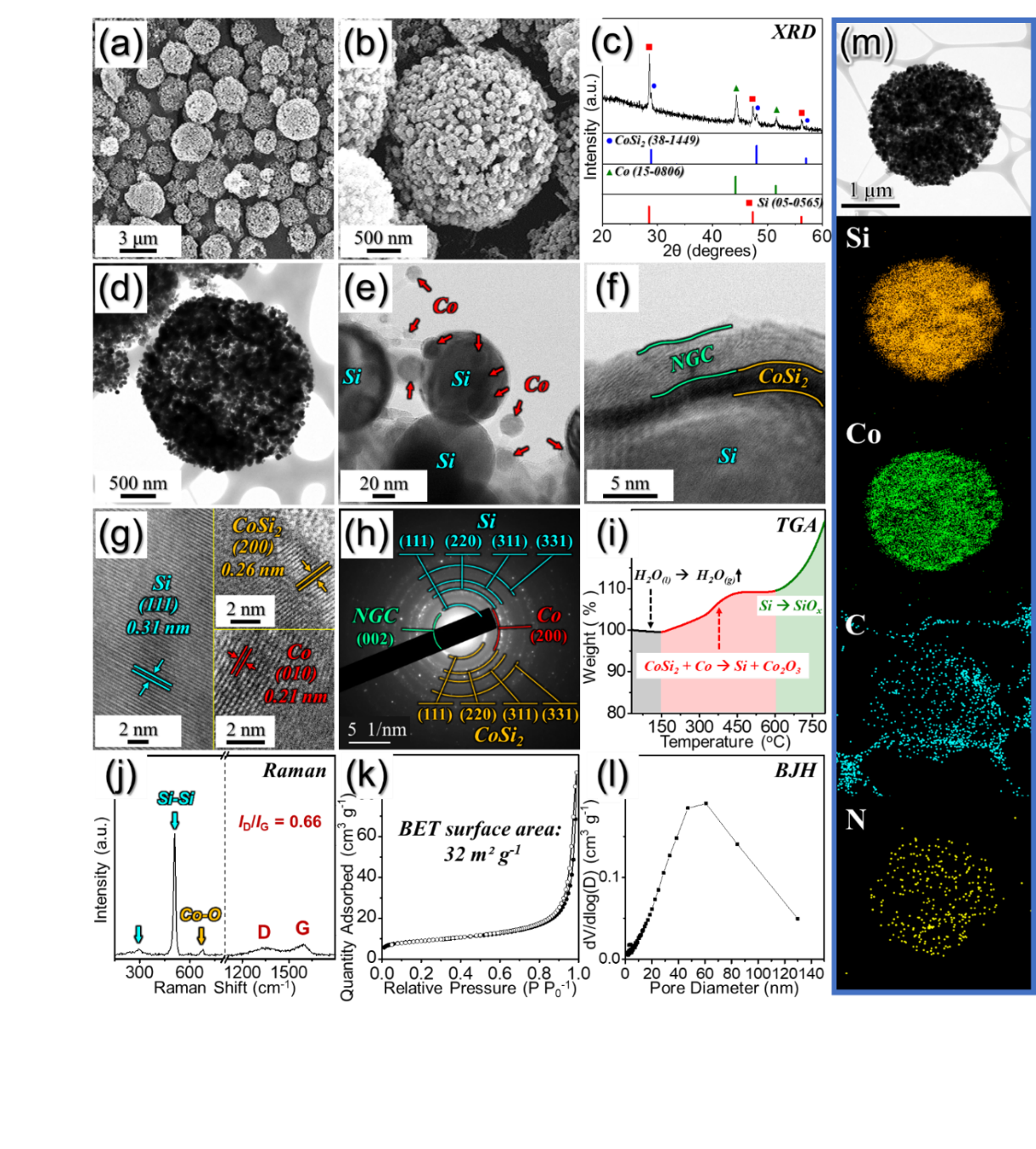
**

**Figure S9.** a,b)FE-SEM images, c) XRD pattern, d–f) TEM images, g) HR-TEM images, h) SAED pattern, i) TGA curve, j) Raman spectrum, k) N_2_ adsorption-desorption isotherms, l) BJH desorption pore-size distribution curve, and m) elemental mapping images of Si@CoSi_2_-Co/NGC microsphere.

The physical characterization results of the Si@CoSi_2_-Co/NGC microspheres without the PDA-C coating shell prepared through the carbonization at 800 ℃ of the as-sprayed Si@CoO/NGC microspheres are presented in Figure S9. The FE-SEM image in Figure S9a illustrates the formation of a homogeneously distributed micron-sized (*ϕ* = 2.5 µm) spherical microspheres. A closer examination of Figure S9b reveals that the microspheres consist of sparsely connected nanocrystals, primarily Si nanoparticles. The morphology appears somewhat less compact than that shown in Figure 2b, mainly due to the absence of a PDA-derived carbonaceous shell, which behaves as an adhesive to hold the constituent units together. Additionally, the sparsely connected nanocrystals within the nanostructure resulted in void formation, contributing to the porosity of the sample. The phase analysis information was obtained from the XRD in Figure S9c, which reveals mixed-phase formation with sharp and high-intensity peaks indexed to the Si, CoSi_2_, and metallic-Co phases. The TEM results in Figure S9d support the FE-SEM findings, confirming the formation of spherically shaped microspheres with numerous pores due to the sparse distribution of constituent units. The HR-TEM image in Figure S9e indicates the presence of Si nanocrystals (*ϕ* = 50 nm) with metallic-Co nanoparticles (*ϕ* = 25 nm) firmly attached to the Si-surface. The magnified TEM image in Figure S9f reveals the presence of a ~2 nm thick CoSi_2_ around the Si. Moreover, a PVP-derived ~4 nm thick NGC shell is evident. The TEM results confirmed the formation of a nanostructure comprising a Si as the primary unit surrounded by metallic-Co nanoparticles with CoSi_2_ and NGC protective materials. Notably, no additional coating layers were observed, confirming the absence of a PDA-C shell. The HR-TEM images (Figure S9g) and SAED pattern (Figure S9h) also indicate lattice fringes and diffraction rings corresponding to Si, CoSi_2_, and metallic-Co. Furthermore, the TG curve shown in Figure S9i displays no weight loss due to the combustion of carbonaceous materials, indicating a minimal amount of carbon content (mainly NGC) in the prepared Si@CoSi_2_-Co/NGC microspheres. Notably, most of the PVP-derived amorphous carbon in the structure was mainly consumed in the carbothermic reduction of CoO species during the carbonization process. The low intensities of the D- and G-bands in the Raman spectrum (Figure S9j) further validate the above results. However, a low *I_D_/I_G_* value of 0.66 suggests that carbonaceous products are mainly graphitic nature. Moreover, while an N content of 0.3 wt% suggests N-doping within the carbonaceous species, this relatively low N content signifies low defects within the carbon shell, resulting in a low surface area. This observation is consistent with the BET curves for Si@CoSi_2_-Co/NGC, which reveal the lowest surface area of only 32 m^2^ g^−1^ (Figure S9k). This is primarily due to the absence of a PDA-derived carbon shell, which induced micropores in the nanostructure, as evident from the BJH pore size distribution (Figure S9l). In addition, the absence of Co-derived pores with a diameter ~7 nm is obvious owing to the absence of a carbon shell, leaving only void-induced macro-sized open pores. Finally, the elemental dot mapping images in Figure S9m provide clear evidence of the uniform dispersion of Si, Co, C, and N, corroborating the formation of an N-doped carbon comprising Si@CoSi_2_ and metallic-Co nanocrystals. However, the sparse distribution of the C element suggests a low amount of carbonaceous species. This observation aligns well with the EA analysis results (Table S2), which reveal a carbon content of only 0.5 wt%. Overall, these results indicate the formation of spherically shaped microspheres comprising sparsely connected Si@CoSi_2_ nanocrystals and metallic-Co nanoparticles coated with trace NGC.

**
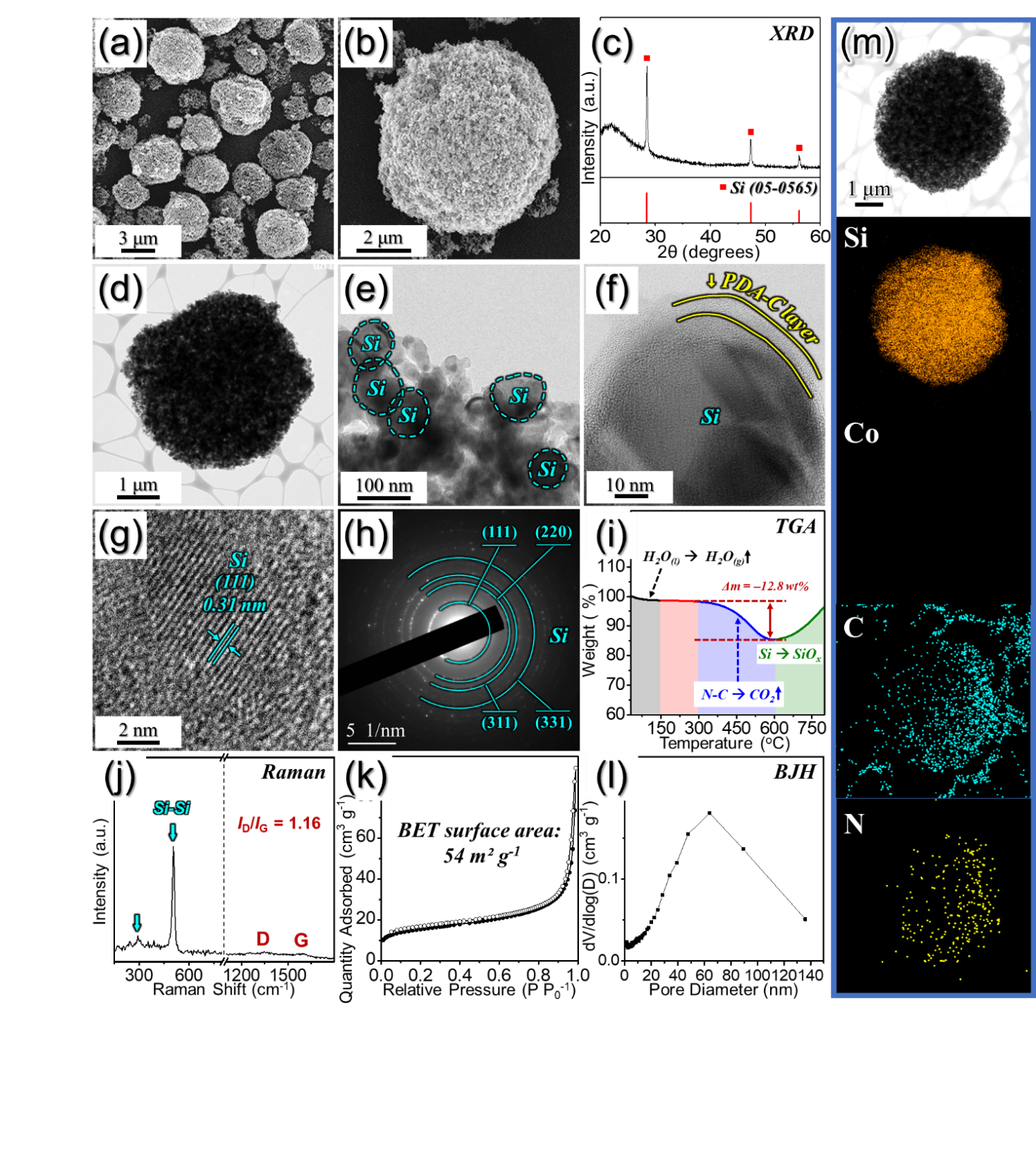
**

**Figure S10.** a,b) FE-SEM images, c) XRD pattern, d–f) TEM images, g) HR-TEM images, h) SAED pattern, i) TGA curve, j) Raman spectrum, k) N_2_ adsorption-desorption isotherms, l) BJH desorption pore-size distribution curve, and m) elemental mapping images of Si/N-C@PDA-C microspheres.

Another comparison sample without Co-salt was prepared using the spray drying process. The as-sprayed Si/PVP microspheres were subjected to the PDA-C coating process, followed by an additional heat-treatment in the N_2_ atmosphere and abbreviated as “Si/N-C@PDA-C” microspheres. The physical characterization results of the resulting Si/N-C@PDA-C microspheres are summarized in Figure S10. The FE-SEM micrograph in Figure S10a reveals the synthesis of microspheres with an average diameter of ~4 µm. In a higher magnification micrograph (Figure S10b), small-sized nanocrystals of Si are seen clustering within a carbon shell derived from PVP. The XRD pattern in Figure S10c reveals sharp, intense peaks indicative of the Si crystal phase. The TEM image (Figure S10d) complements the FE-SEM micrograph shown in Figure S10b, demonstrating the formation of a microsphere composed of Si nanocrystals bundled within a carbon shell, as is evident from the magnified TEM image in Figure S10e. The highly magnified TEM image in Figure S10f depicts the presence of a ~5 nm thick PDA-derived carbon shell enveloping the Si nanocrystal. The HR-TEM (Figure S10g) and SAED (Figure S10h) patterns solidify these observations, with clear lattice fringes and diffraction rings corresponding exclusively to the diffraction planes of Si. Moreover, the carbon and nitrogen content within the microsphere are estimated to be 9.8 wt% and 2.0 wt% by the EA results (Table S2), respectively. The TG curve in Figure S10i also predicts a carbon content of 12.8 wt%, which closely matches the EA results in Table S2. The Raman analysis in Figure S10j shows peaks located at 292 and 506 cm^−1^ corresponding to Si–Si bonds.^[40]^ Furthermore, the high *I*_D_/*I*_G_ value of 1.16 indicates that the carbonaceous shell is primarily amorphous nature due to the absence of Co-species serving as a graphitization catalyst. The BET curves in Figure S10k reveal a low surface area of 54 m^2^ g^−1^ compared to Si@CoSi_2_-Co/NGC@PDA-C mainly due to the absence of Co-derived pores, as indicated in the BJH pore size curve in Figure S8l. Additionally, the higher BET surface value compared to Si@CoSi_2_-Co/NGC (Figure S10l) is due to the relatively higher micropore volume induced by a PDA-derived N-doped carbon shell. The elemental dot mapping images in Figure S10m confirm a uniform dispersion of Si, C, and N elements within the nanostructure, affirming the successful synthesis of Si/N-C@PDA-C microspheres.


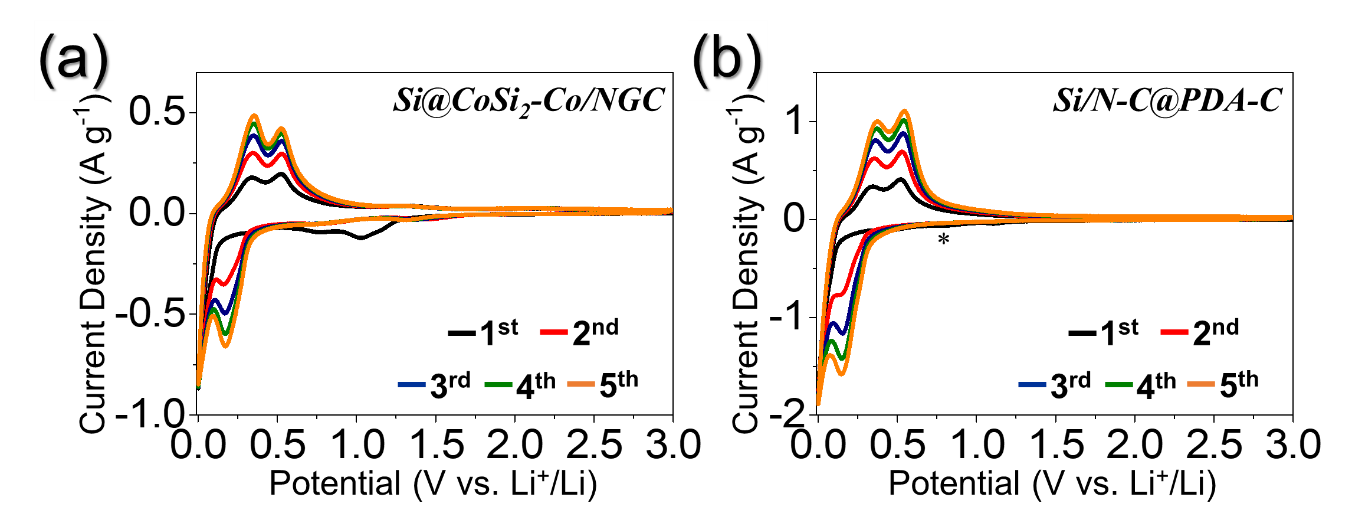


**Figure S11.** CV curves of (a) Si@CoSi_2_-Co/NGC and (b) Si/N-C@PDA-C microspheres.

**Table S3.** Comparison of the electrochemical performance of various Si/carbon and Si/Me*_x_*Si*_y_* (Me=Co, Ti, Fe, Cu) composite-based anode materials for Li-ion storage.

| Electrode Materials | Voltage  Range  (V) | Current  density  [A g^−1^] | Discharge  capacity  (CC / CC-CV)  [mA h g^−1^]  (Cycle No.) | Capacity retention  (CC / CC-CV)  [%] | Rate capacity  (CC / CC-CV)  [mA h g^−1^]  /Current density  [A g^−1^] | Ref. |
| --- | --- | --- | --- | --- | --- | --- |
| **Si@CoSi_2_-Co/NGC@PDA-C** | **0.001–3.0** | **1.0** | **900 (600)**  **1002 (150)** | **88**  **80** | **1299 / 1440 (0.1)**  **1200 / 1370 (0.2)**  **1021 / 1293 (0.5)**  **817 / 1204 (1.0)**  **611 / 1056 (2.0)**  **315 / 710 (5.0)**  **236 / 561 (7.0)**  **177 / 428 (10)** | **This work** |
| **< Si/carbon composite-based anode materials >** | | | | | | |
| Si@C@void@CNT | 0.001–2.0 | 0.1 | 913 (100) | - | 1713 (0.1)  1302 (0.2)  834 (0.5)  671 (1.0)  450 (2.0) | [S1] |
| Si/AC@GC composite microsphere | 0.001–1.2 | 1.0 | 803 (200) | 66 | 1412 (0.1)  1201 (0.5)  1023 (1.0)  809 (2.0)  663 (3.0)  622 (4.0)  589 (5.0) | [S2] |
| Si@N-doped C coupled with 2D MXene nanosheets | 0.01–3.0 | 1.0 | 953 (300) | 51 | 1834 (0.5)  1719 (1.0)  1708 (2.0)  1200 (5.0)  849 (10) | [S3] |
| Si@multichannel  carbon fibers | 0.01–2.0 | 0.1 | 771 (150) | 64 | 1342 (0.1)  1331 (0.2)  1215 (0.5)  1007 (1.0)  784 (1.5) | [S4] |
| Silicon/carbon with 3D hierarchical macro-/mesoporous silicon network | 0.005–3.0 | 0.2 | 710 (300) | 79 | 921 (0.1)  749 (0.2)  641 (0.5)  491 (1.0)  316 (2.0)  112 (5.0) | [S5] |
| **< Si/CoSi_2_ composite-based anode materials >** | | | | | | |
| Si/Co-CoSi_2_/rGO nanocomposite | 0.01–3.0 | 0.1 | 952 (80) | 80 | 1334 (0.1)  844 (0.5)  744 (1.0)  571 (2.0)  195.2 (5.0) | [S6] |
| porous Si-CoSi_2_-C | 0.001–2.0 | 0.1 | 1005 (100) | 63 | 1197 (0.1)  1133 (0.2)  1010 (0.5)  875 (1.0)  747 (2.0)  598 (5.0) | [S7] |
| Si@CoSi_2_/Co-N-doped porous carbon@CNTs | 0.001–3.0 | 0.5 | 1071 (200) | 73.3 | 1449 (0.5)  1391 (1.0)  1262 (2.0)  1110 (4.0)  930 (6.0) | [S8] |
| CoSi*_x_*/Si/C-composite | 0.02–1.5 | 0.5 | 672 (200) | 73 | 930 (0.1)  832 (0.3)  760 (0.5)  715 (0.7)  654 (1.0)  562 (1.5) | [S9] |
| **< Si/Me*_x_*Si*_y_* (Me=Ti, Ni, Fe, Cu) composite-based anode materials >** | | | | | | |
| Si/TiSi_2_ composites | 0.01–1.5 | 0.8 | 530 (200) | 96 | 759 (0.2)  628 (0.4)  517 (0.8)  456 (1.2)  422 (1.6) | [S10] |
| Si/NiSi_2_/C composite | 0.01–2.0 | 0.2 | 1828 (50) | 88 | 2226 (0.2)  1947 (0.5)  1682 (1.0)  1502 (2.0)  1364 (3.0) | [S11] |
| SiO*_x_*@Si-FeSi_2_ nanocomposite | 0.01–1.5 | 0.5 | 843 (200) | 80 | 1637 (0.25)  1231 (0.5)  1096 (1)  977 (1.5) | [S12] |
| Cu_3_Si-modified SiNPs encapsulated within SiO*_x_* and hollow carbon | 0.01–1.5 | 1.0 | 819 (200) | 86 | 1263 (0.42)  1156 (0.84)  1033 (1.26)  870 (2.1)  667 (4.2)  450 (8.4) | [S13] |


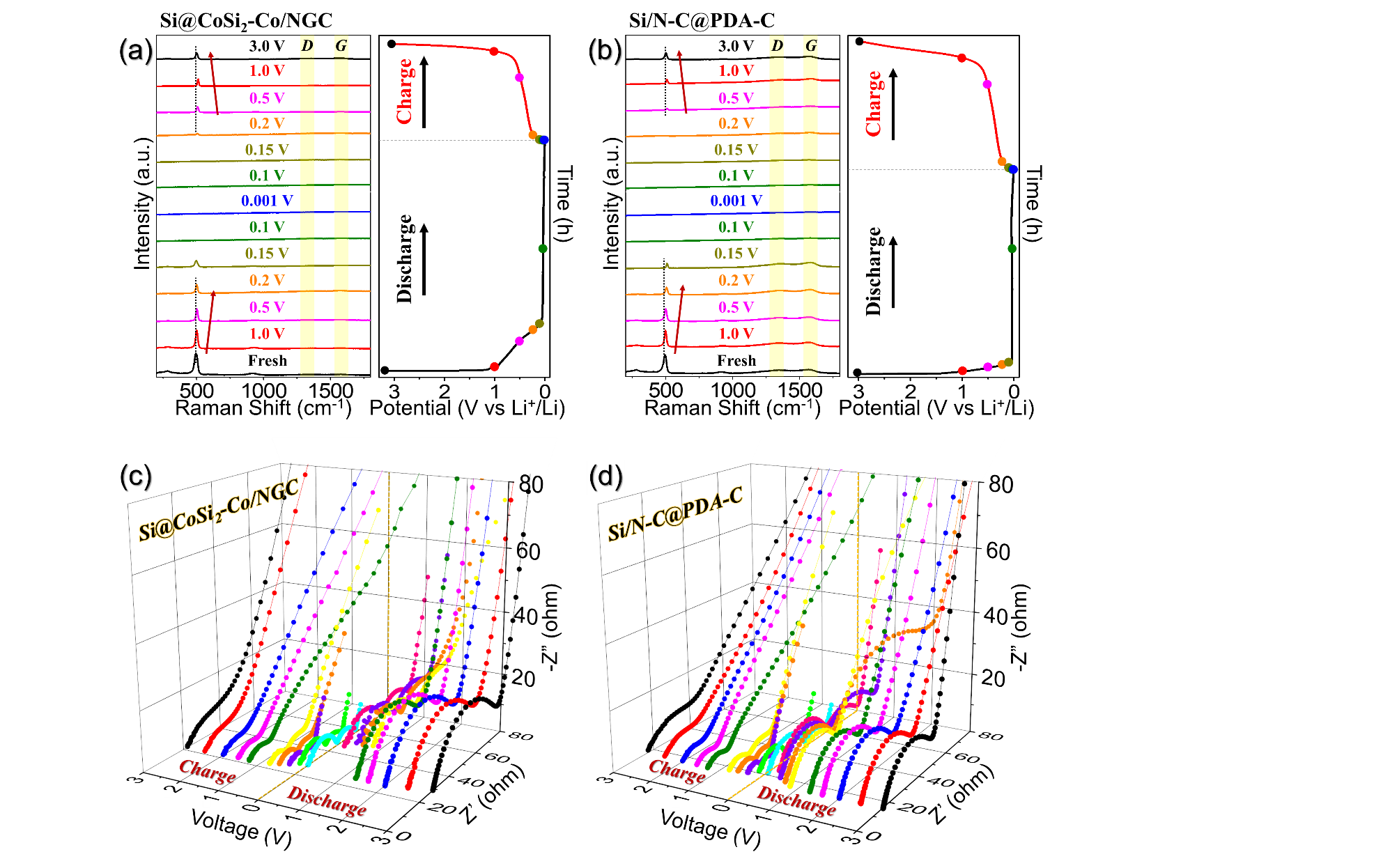


**Figure S12.** a,b) *Ex-situ* Raman spectra and c,d) *in-situ* EIS profiles of the a,c) Si@CoSi_2_-Co/NGC and b,d) Si/N–C@PDA-C anode collected at various charge/discharge states during the initial cycle at 0.1 A g^−1^.


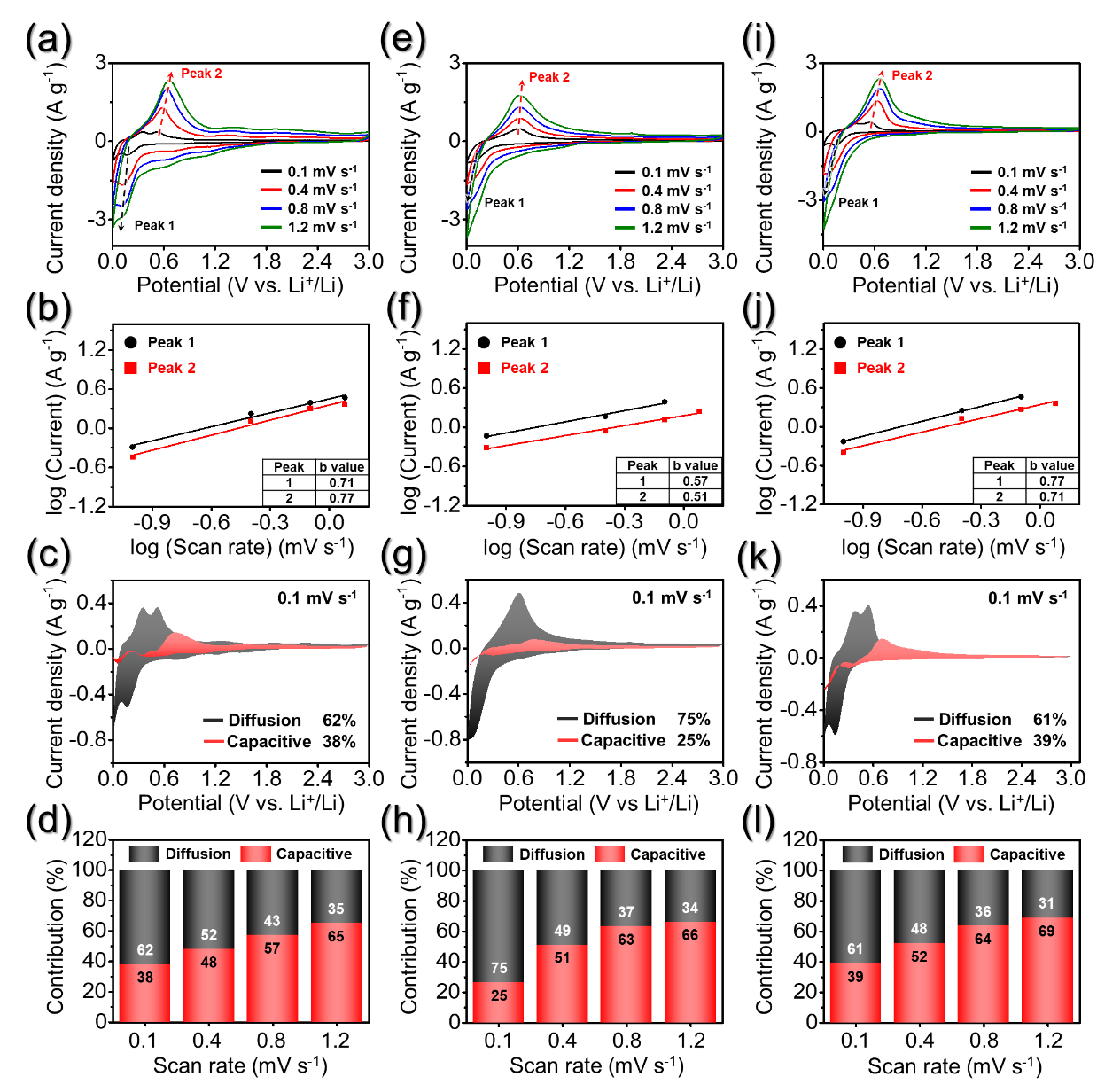


**Figure S13.** Electrochemical reaction dynamics analysis of a–d) Si@CoSi_2_-Co/NGC@PDA-C, e–h) Si@CoSi_2_-Co/NGC, and i–l) Si/N-C@PDA-C microspheres for Li-ion storage: a,e,i) CV curves obtained at various scan rates, b,f,j) current response (*i*) vs. scan rate (*n*) at each redox peak, c,g,k) CV curves with the capacitive fraction shown by the red region at a scan rate of 0.1 mV s^–1^ , and d,h,l) bar chart showing the percentage of the capacitive contribution at different scan rates.

**
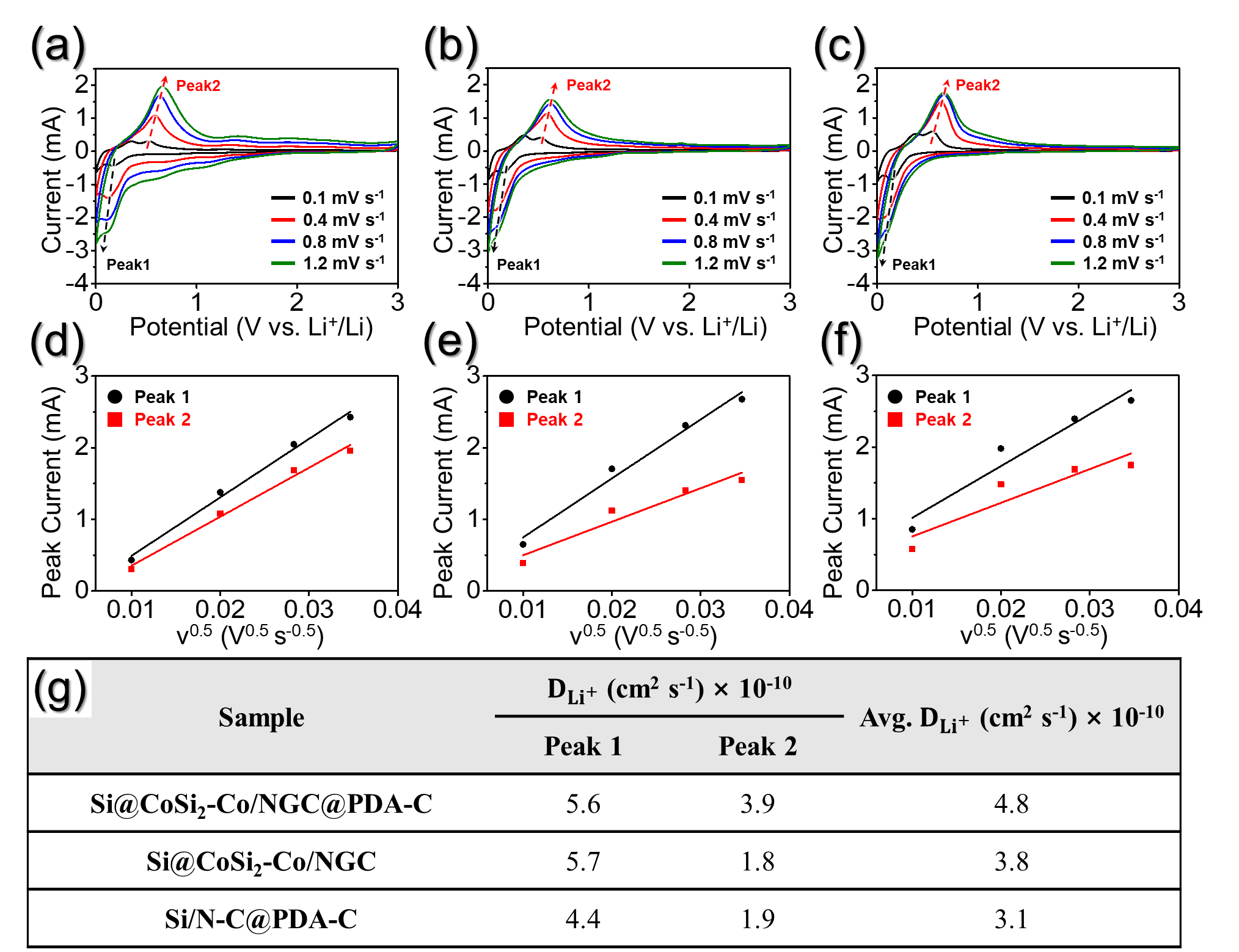
**

**Figure S14.** Li-ion diffusion coefficient ($D_{{Li}^{+}}$) for the LIB; a,d) Si@CoSi_2_-Co/NGC@PDA-C, b,e) Si@CoSi_2_-Co/NGC, c,f)**f** Si/N-C@PDA, and g) Li-ion diffusion coefficient comparison table.


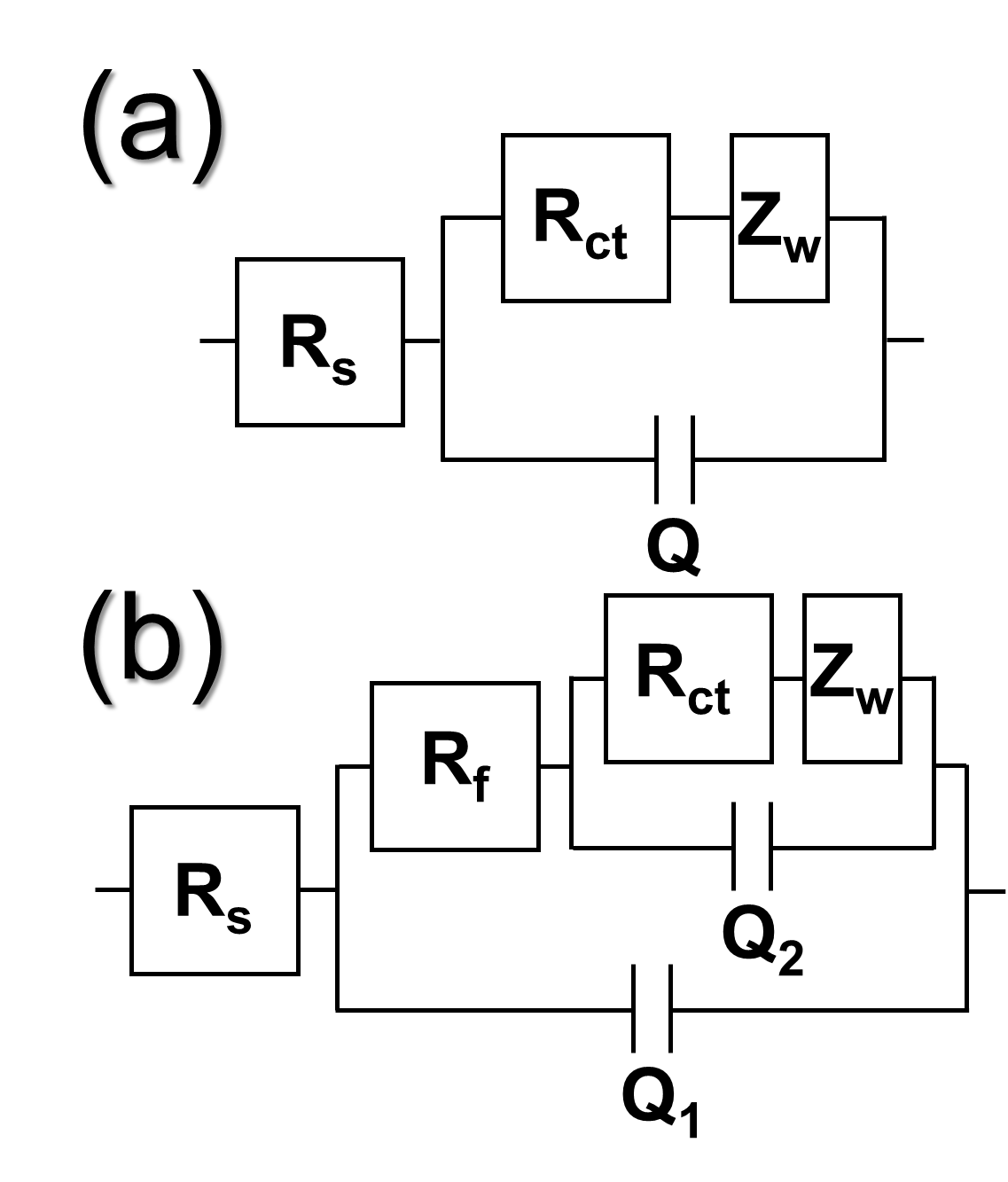


**Figure S15.** Equivalent circuit model used for AC impedance fitting; a) before cycling, and b) after cycling, *R_ct_* = charge-transfer resistance, *R_s_* = solution resistance, *R_f_* = SEI layer resistance, *Q_1_* = dielectric relaxation capacitance, *Q_2_* = associated double layer capacitance.

**Table S4.** EIS fitted parameters for each Li-ion cells utilizing three samples as anode materials before cycle, after 1^st^, and after 500^th^ cycle at 3.0 A g^–1^.

|  | **Fresh cell** | | **After 1^st^ cycle** | | **After 500^th^ cycle** | |
| --- | --- | --- | --- | --- | --- | --- |
|  | **R_s_ (Ω)** | **R_ct_ (Ω)** | **R_s_ (Ω)** | **R_ct_ (Ω)** | **R_s_ (Ω)** | **R_ct_ (Ω)** |
| Si@CoSi_2_-Co/NGC@PDA-C | 18 | 137 | 17 | 30 | 15 | 24 |
| Si@CoSi_2_-Co/NGC | 17 | 240 | 17 | 64 | 19 | 38 |
| Si/N-C@PDA-C | 15 | 243 | 16 | 45 | 13 | 44 |


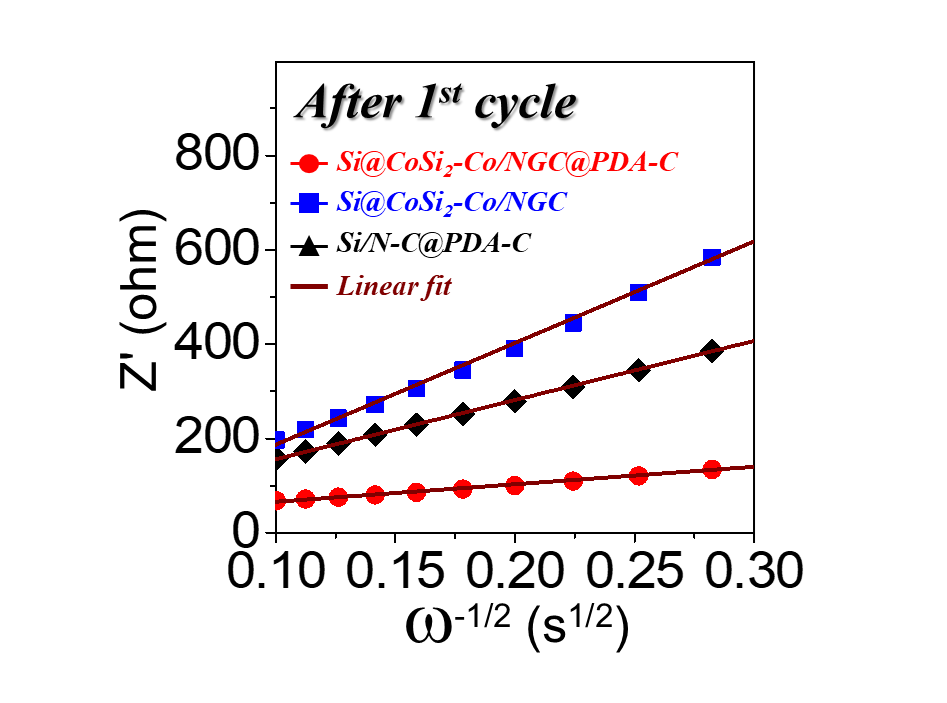


**Figure S16.** Relationships between the real part of the impedance (*Z*_re_) and *ω*^−1/2^ of the samples after 1^st^ cycle.

**
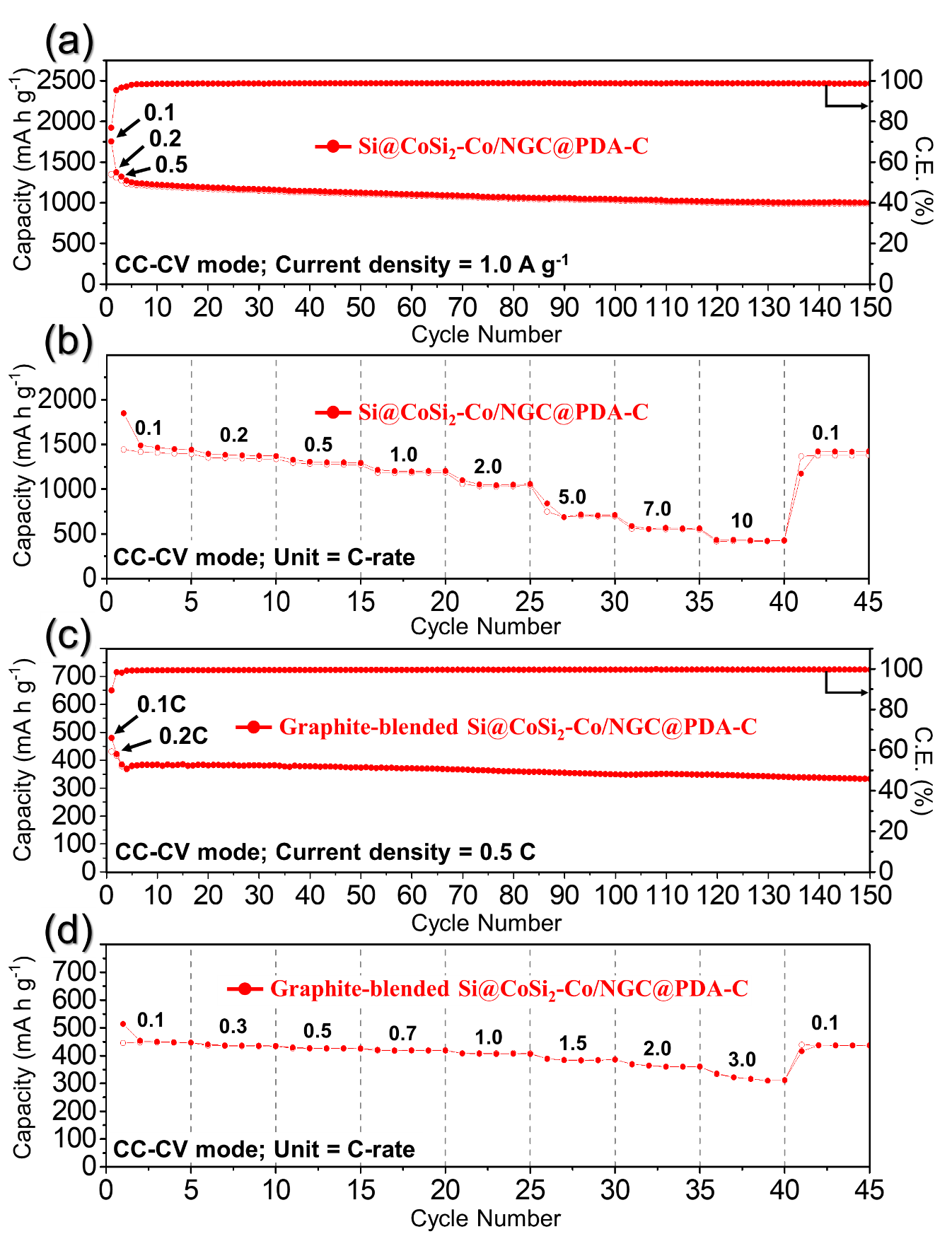
**

**Figure S17.** Electrochemical properties of Si@CoSi_2_-Co/NGC@PDA-C anode under constant current-constant voltage (CC-CV) mode: a) cycling performance at current densities of 1.0 A g^−1^, b) rate performance, c) cycling performance of graphite-blended Si@CoSi_2_-Co/NGC@PDA-C anode at 0.5 C (1.0 C = 480 mA g^−1^), and d) rate performance of graphite-blended Si@CoSi_2_-Co/NGC@PDA-C anode.


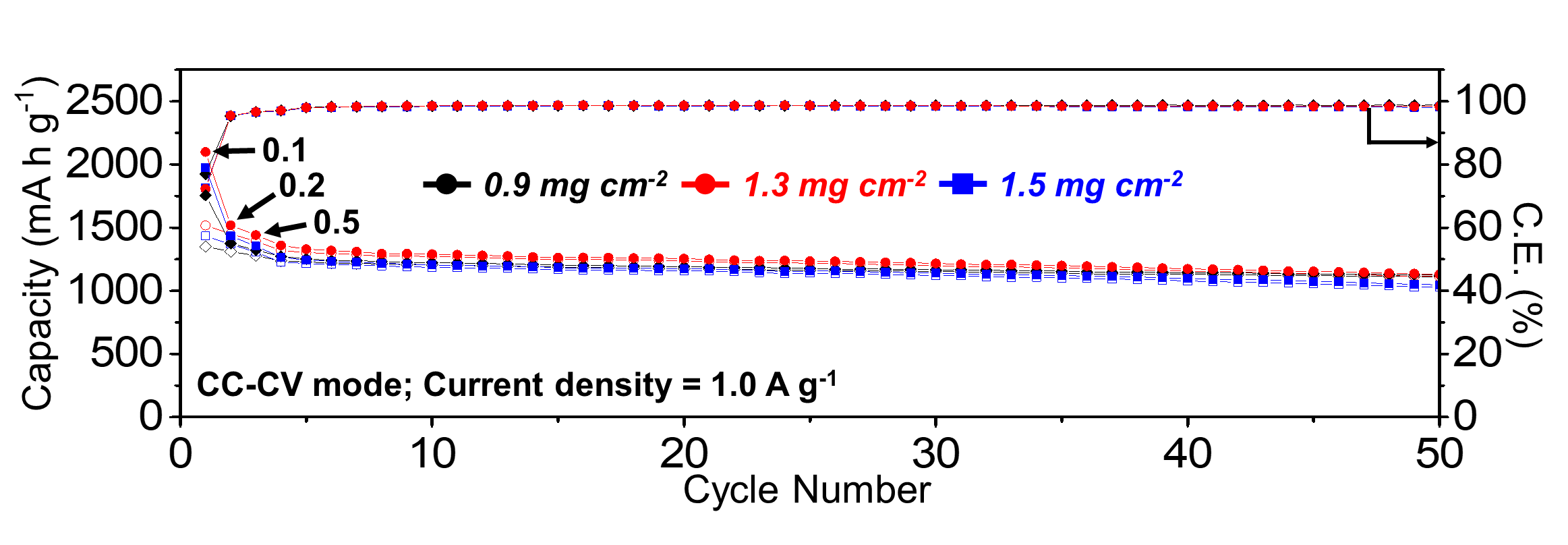


**Figure S18.** Cycling performance of the Si@CoSi_2_-Co/NGC@PDA-C anodes with different areal active material loadings of 0.9, 1.3 and 1.5 mg cm^−2^ at a current density of 1.0 A g^−1^.


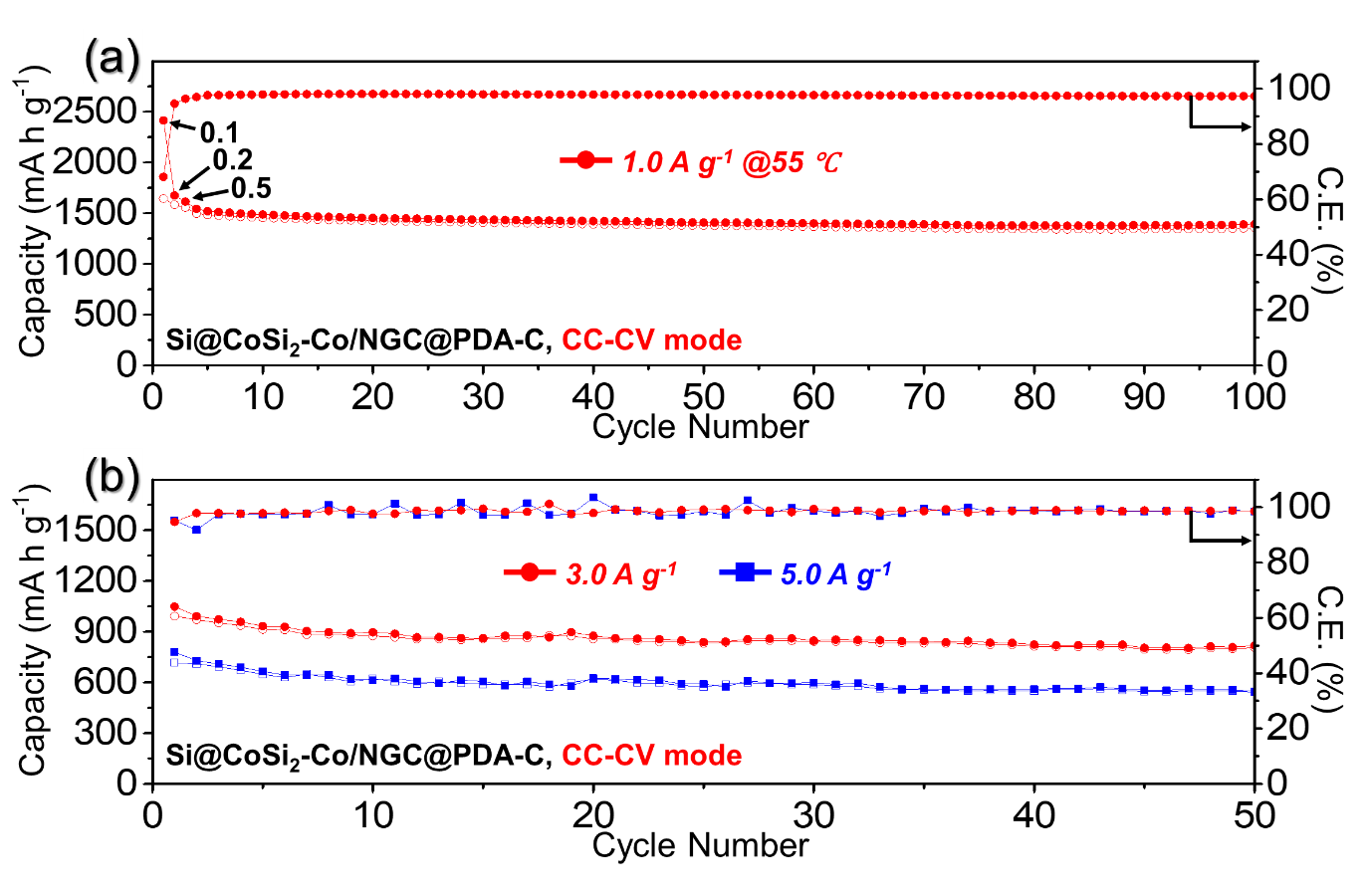


**Figure S19.** Cycling performance of the Si@CoSi_2_-Co/NGC@PDA-C anodes under a) high-temperature condition of 55 ℃ at 1.0 A g^−1^, and b) high-rate conditions at 3.0 and 5.0 A g^−1^, tested under CC-CV mode.

**
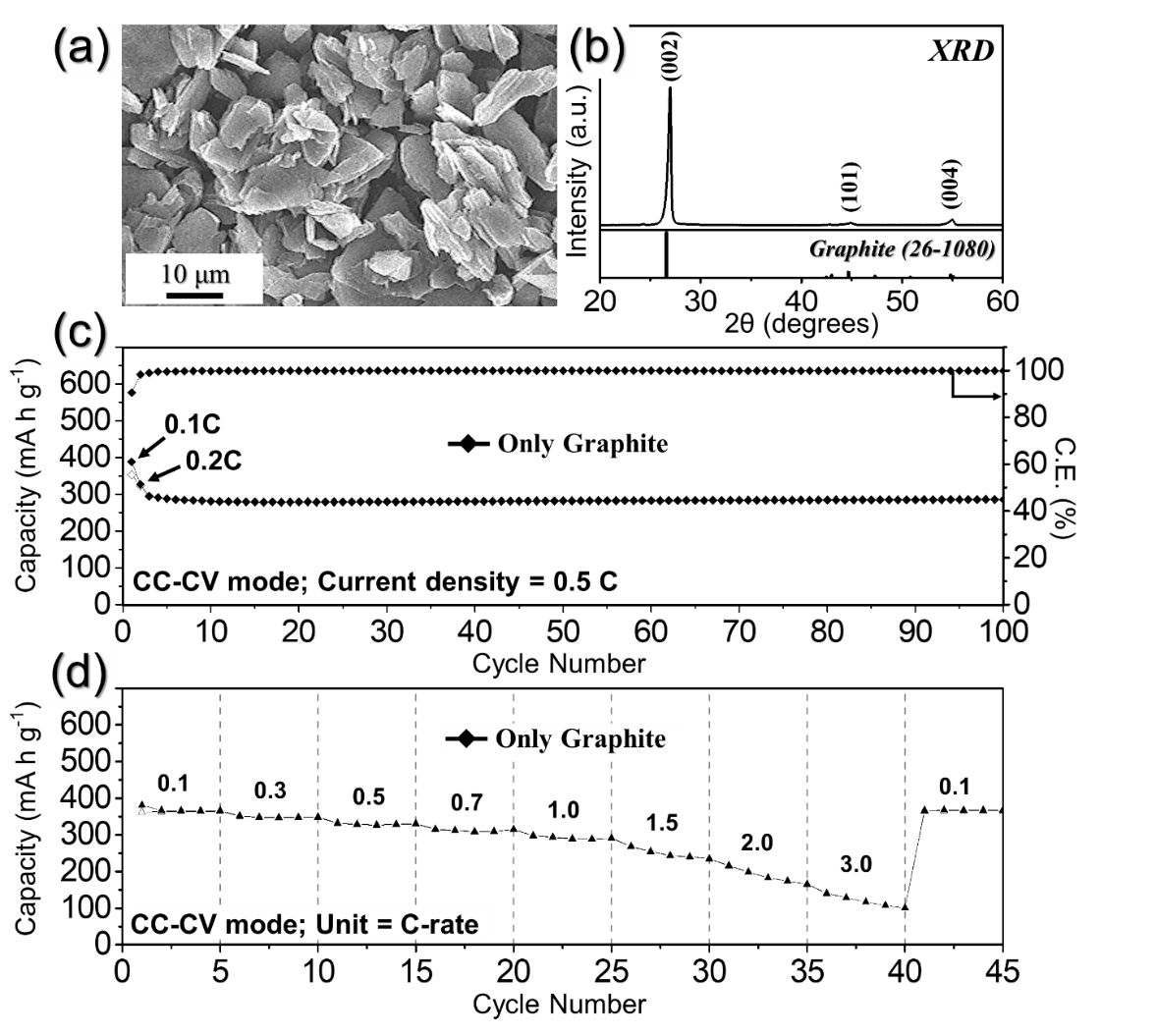
**

**Figure S20.** a) FE-SEM image, b) XRD pattern, c) cycling performance at 0.5 C (1.0 C = 372 mA g^−1^), and d) rate performance of commercial graphite for blending tests.

The FE-SEM and XRD analysis, along with the cycling and rate performance results of the commercial graphite used for the blending tests, are summarized in Figure S20. The FE-SEM images and XRD patterns confirm the flake morphology of commercial graphite, with particle sizes exceeding 10 µm and a well-defined crystalline phase corresponding to graphite (Figure S20a and b). The cycling performance of graphite evaluated under CC-CV conditions at a current density of 0.5 C (1.0 C = 372 mA g^−1^) demonstrates a reversible capacity of 287 mA h g^–1^ after 150 cycles (Figure S20c). In the rate performance, graphite exhibits a discharge capacity of 366 mA h g^−1^ at a low current density of 0.1 C, nearly approaching its theoretical capacity (Figure S20d). However, the rate capability declined at current densities above 1.0 C, attributed to the intrinsically low Li-ion diffusion properties of graphite.

**
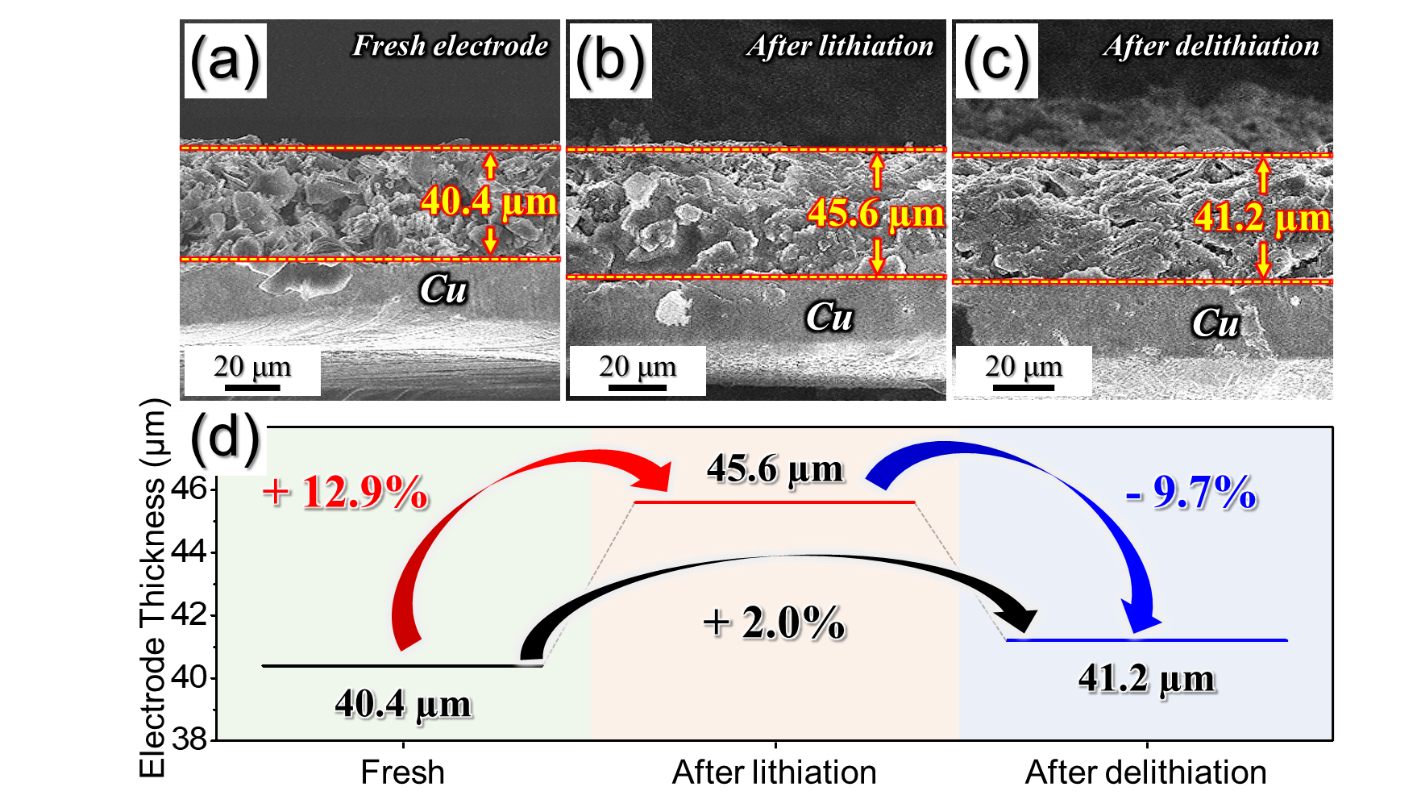
**

**Figure S21.** Cross-sectional FE-SEM images of the graphite-blended Si@CoSi_2_-Co/NGC@PDA-C anode: a) Fresh electrode, b) after lithiation, c) after delithiation process at a current density of 0.1 C (1.0 C = 480 mA g^−1^); and d) electrode swelling rate during the initial lithiation/delithiation process for graphite-blended Si@CoSi_2_-Co/NGC@PDA-C anode.

**
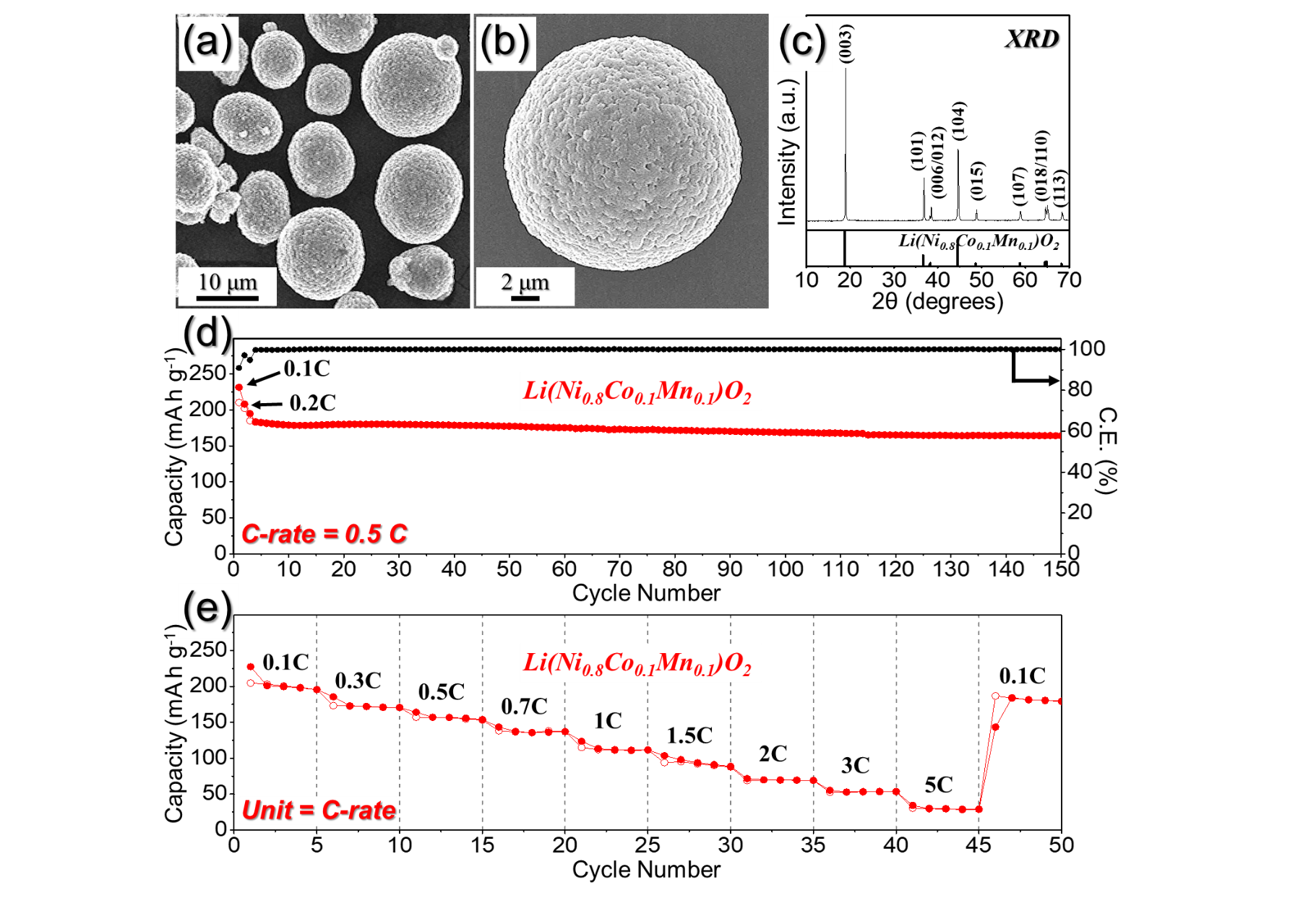
**

**Figure S22.** a,b) FE-SEM images, c) XRD pattern, d) cycling performance at 0.5 C of commercial Li(Ni_0.8_Co_0.1_Mn_0.1_)O_2_ cathode (1.0 C = 180 mA g^−1^), and e) rate capability results.

**Table S5.** The gravimetric energy density of Li-ion full-cell under various mass loading.

| **Loading (P/N)**  **[mg cm^−2^]** | **Discharge capacity/C-rate**  **[mA h g^−1^]** | **Gravimetric energy density**  **[Wh kg^−1^]** |
| --- | --- | --- |
| 4.52/2.10 | 169/0.5 | 406 |
| 20.9/6.55 | 176/0.1 | 469 |

**Gravimetric energy density** = $\frac{Discharge capacity (A h) \times Voltage (3.5 V)}{Material weight (kg)}$

**Voltage = 3.5 V**

*Worked example for gravimetric energy density for* ***typical*** *Li-ion full-cell :*

wt. of NCM811 = 7.0 mg = 0.007 g

wt. of Graphite-Si@CoSi_2_-Co/NGC@PDA-C = 3.2 mg = 0.0032 g

**🡪 wt. of (NCM811 + Graphite-Si@CoSi_2_-Co/NGC@PDA-C) = 10.2 mg = 0.0102 g**

Specific discharge capacity = **169 mAh g^−1^** at 100^th^ cycle for 0.5 C

Energy of Li-ion full-cell = 169 × 3.5 × 0.007 = 4.14 mWh

**Gravimetric energy density** = 4.14 / 0.0102 = **406 Wh kg^−1^**

*Worked example for gravimetric energy density for* ***high loading*** *Li-ion full-cell :*

*At active material level:*

wt. of NCM811 = 32.2 mg = 0.0322 g

wt. of Graphite-Si@CoSi_2_-Co/NGC@PDA-C = 10.1 mg = 0.0101 g

**🡪 wt. of (NCM811 + Graphite-Si@CoSi_2_-Co/NGC@PDA-C) = 42.3 mg = 0.0423 g**

Specific discharge capacity = **176 mAh g^−1^** at 45^th^ cycle for 0.1 C

Energy of Li-ion full-cell = 176 × 3.5 × 0.0322 = 19.84 mWh

**Gravimetric energy density** = 19.84 / 0.0423 = **469 Wh kg^−1^**


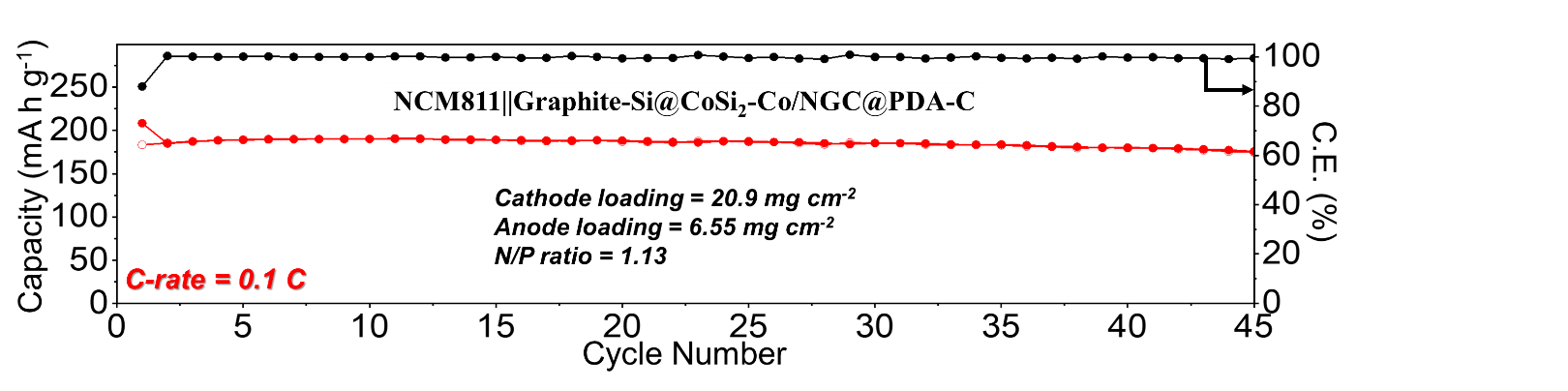


**Figure S23**. Cycling performance of the NCM811||graphite-blended Si@CoSi_2_-Co/NGC@PDA-C full-cell under a high areal mass loading condition (20.9 mg cm^−2^ for the NCM811 cathode and 6.55 mg cm^−2^ for the anode) at 0.1 C.

**
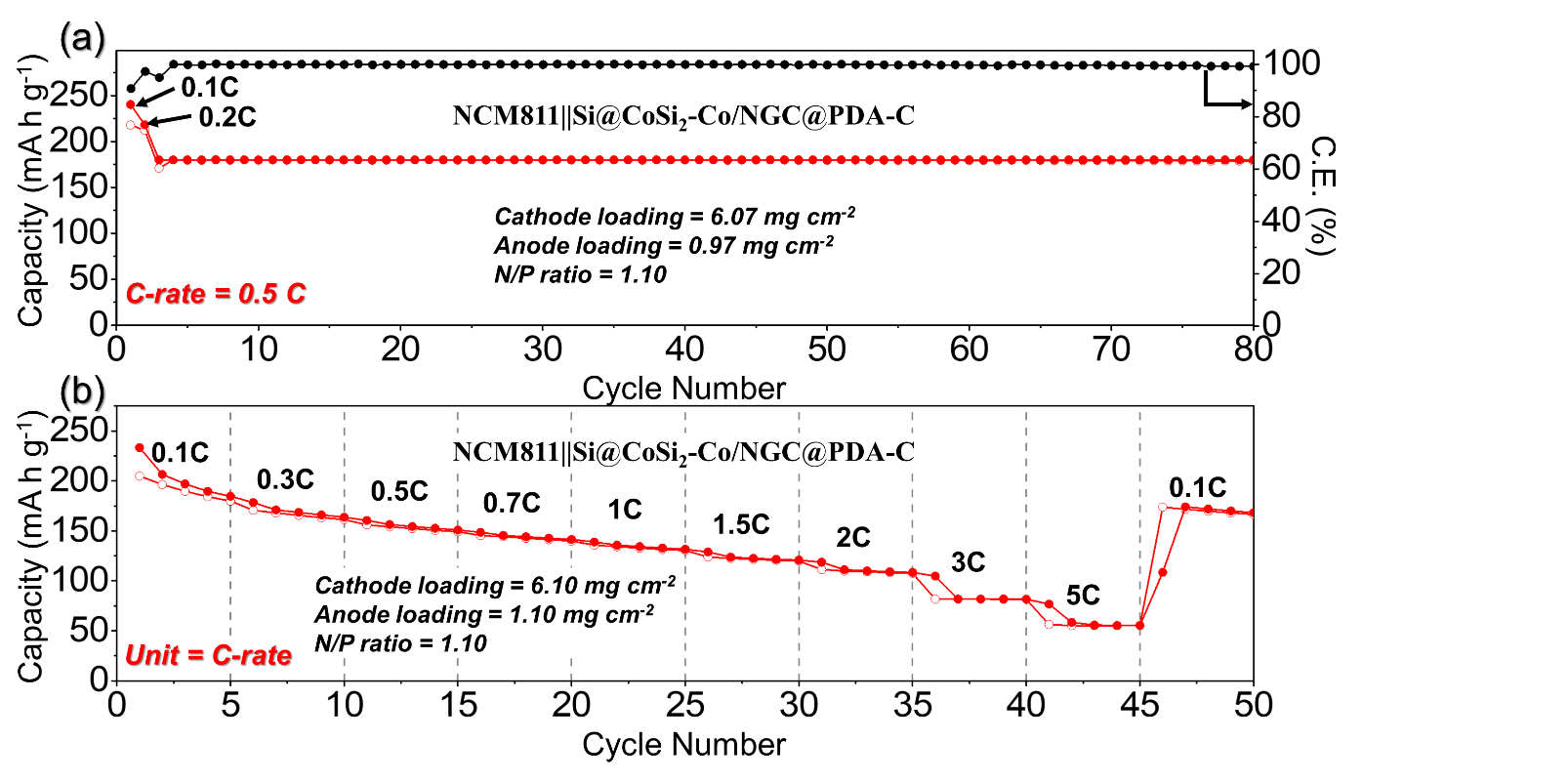
**

**Figure S24.** Full-cell electrochemical performance of Li(Ni_0.8_Co_0.1_Mn_0.1_)O_2_ (NCM811) cathode and Si@CoSi_2_-Co/NGC@PDA-C anode without graphite-blend: a) cycling stability at 0.5 C and b) rate performance evaluated at various C-rates from 0.1 to 5.0 C.

**References**

[S1] Yin, L., Park, M., Jeon, I., et al., Silicon nanoparticle self-incorporated in hollow nitrogen-doped carbon microspheres for lithium-ion battery anodes, 2021, Electrochim. Acta, 368, 137630, https://doi.org/10.1016/j.electacta.2020.137630

[S2] Yang, S. H., Kim, J. K., Jung, D.-S., & Kang, Y. C., Facile fabrication of Si-embedded amorphous carbon@graphitic carbon composite microspheres via spray drying as high-performance lithium-ion battery anodes, 2022, Appl. Surf. Sci., 606, 154799, https://doi.org/10.1016/j.apsusc.2022.154799

[S3] Jo, D. Y., Kim, J. K., Oh, H. G., Kang, Y. C., & Park, S.-K., Chemically Integrating MXene Nanosheets with N-Doped C-Coated Si Nanoparticles for Enhanced Li Storage Performance, 2021, Scr. Mater., 199, 113840, https://doi.org/10.1016/j.scriptamat.2021.113840

[S4] Chen, X., Hu, P., Xiang, J., Zhang, R., & Huang, Y., Confining Silicon Nanoparticles within Freestanding Multichannel Carbon Fibers for High-Performance Li-Ion Batteries, 2019, ACS Appl. Energy Mater., 2, 5214, https://doi.org/10.1021/acsaem.9b00898

[S5] Zuo, X., Wang, X., Xia, Y., Yin, S., Ji, Q., Yang, Z., Wang, M., Zheng, X., Qiu, B., Liu, Z., & Zhu, J., Silicon/carbon lithium-ion battery anode with 3D hierarchical macro-/mesoporous silicon network: Self-templating synthesis via magnesiothermic reduction of silica/carbon composite, 2019, J. Power Sources, 412, 93, https://doi.org/10.1016/j.jpowsour.2018.11.039

[S6] Park, A. R., Nam, M. G., Kim, A. Y., et al., Si/Co-CoSi_2_/reduced graphene oxide ternary nanocomposite anodes for Li-Ion batteries with enhanced capacity and cycling stability, 2017, J. Alloys Compd., 724, 1134, https://doi.org/10.1016/j.jallcom.2017.07.119

[S7] Seo, H., Yang, H. -R., Yang, Y., Kim, K., Kim S. H., Lee, H., & Kim, J. H., Scalable synthesis and electrochemical properties of porous Si-CoSi_2_-C composites as an anode for Li-ion batteries, 2021, Materials, 14, 5397, https://doi.org/10.3390/ma14185397

[S8] Qiu, Y., Zhang, C., Zhang, C., et al., CNTs-intertwined and N-doped porous carbon wrapped silicon anode for high performance lithium-ion batteries, 2021, J. Alloys Compd., 877, 160240, https://doi.org/10.1016/j.jallcom.2021.160240

[S9] Ruttert, M., Siozios, V., Winter, M., & Placke, T., Synthesis and comparative investigation of silicon transition metal silicide composite anodes for lithium ion batteries, 2019, *Z.* Anorg. Allg. Chem., 645, 248, https://doi.org/10.1002/zaac.201800436

[S10] Zhang, Y., Chen, M., Chen, Z., et al., Constructing cycle-stable Si/TiSi_2_ composites as anode materials for lithium ion batteries through direct utilization of low-purity Si and Ti-bearing blast furnace slag, 2021, J. Alloys Compd., 876, 160125, https://doi.org/10.1016/j.jallcom.2021.160125

[S11] Zhou, Y., Su, M., Duo, A., & Liu, Y., Facile synthesis of Si/NiSi_2_/C composite derived from metal-organic frameworks for high-performance lithium-ion battery anode, 2020, J. Electroanal. Chem., 873, 114398, https://doi.org/10.1016/j.jelechem.2020.114398

[S12] Reddyprakash, M., Loka, C., Lee, R. K., & Lee, K. -S., High-performance core-shell structured SiO*_x_*@Si-silicide nanocomposite anode material for lithium-ion rechargeable batteries, 2021, J. Electrochem. Soc., 168, 090558, https://doi.org/10.1149/1945-7111/ac2761

[S13] Chen, X., Zhang, X., Cui, J., et al., Cu_3_Si-modified silicon nanoparticles encapsulated within SiO*_x_* and hollow carbon for lithium-ion battery anodes, 2022, ACS Appl. Nano Mater., 5, 14275, https://doi.org/10.1021/acsanm.2c02167

[S14] Forbes, A. W., Dulal, R. P., Bhattarai, N., Pegg, I. L., & Philip, J., Experimental realization and magnetotransport properties of half-metallic Fe_2_Si, 2019, J. Appl. Phys., 125, 243902, https://doi.org/10.1063/1.5096862

[S15] Song, Y., Schmitt, A. L., & Jin, S., Ultralong Single-Crystal Metallic Ni_2_Si Nanowires with Low Resistivity, 2007, Nano Lett., 7, 965, https://doi.org/10.1021/nl0630687
